# Visible Light-Mediated Direct Decarboxylative Giese Amidations without a Photocatalyst—Batch and Flow

**DOI:** 10.1021/acs.joc.6c00672

**Published:** 2026-08-13

**Authors:** Samuel L. Nickels, David M. Kitcatt, Filipe Vilela, Ai-Lan Lee

**Affiliations:** † Institute of Chemical Sciences, 3120 Heriot-Watt University, Edinburgh EH14 4AS, U.K.; ‡ EaStCHEM School of Chemistry, University of Edinburgh, David Brewster Road, Edinburgh EH9 3FJ, U.K.

## Abstract

Previous light-mediated approaches to the direct decarboxylative Giese amidation reaction relied on the use of costly, nonsustainable photocatalysts and faced challenges in efficient scale-up. We have successfully developed a photocatalyst-free, light-mediated methodology via a pathway that is thought to involve *in situ* EDA formation. The reaction is applicable in batch as well as under flow conditions, including efficient scale-up to gram scale in flow.

## Graphical abstract

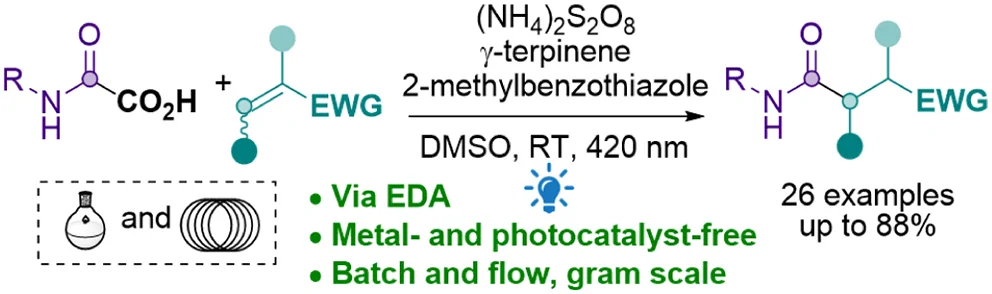

## Introduction

The Giese radical conjugate addition reaction has recently reemerged as a powerful method for constructing C–C bonds that are otherwise not accessible via conventional nucleophilic chemistry.^1^ This current popularity is in large part due to the recent development of mild photocatalytic methodologies.^1^ Giese alkylations and Giese acylation reactions are well established and have been used for various applications such as chemoselective bioconjugation of peptides,^2^ polymerizations,^3^ natural product,^4^ drug molecule,^5^ and unnatural amino acid^6^ syntheses. The development of Giese amidation reactions, in contrast, is less mature, with seminal work reported in 2020 by Konev and Wangelin^7^ and Melchiorre,^8^ using Hantzsch ester derivatives^7,9,10^ and carbamoyl chlorides,^8^ respectively, as activated carbamoyl radical precursors under photocatalytic conditions.^7^ More recent employment of metal oxamates **1** as radical precursors by Kerr and coworkers addressed some of the substrate scope limitations of the earlier reports (Scheme 1A) but was limited to tertiary amides.^11^ We independently reported a direct decarboxylative approach from oxamic acids **4**,^12^ which have significant advantages over previously used radical precursors since oxamic acids **4** were bench-stable, nontoxic, user-friendly, and releases only traceless CO_2_ from the radical precursor.^13^ In order to cover a wide substrate scope under mild photocatalytic conditions, two complementary approaches were necessary: the reductive quenching method (Conditions X, Scheme 1B) was best for installing tertiary amides, while an alternative oxidative method (Conditions Y, Scheme 1B) was generally found to give higher yields for primary and secondary amides. Direct decarboxylative Giese amidation reactions using **4** have also since been elegantly applied to the site-selective construction of *N*-linked glycopeptides.^14^

**Scheme 1:**
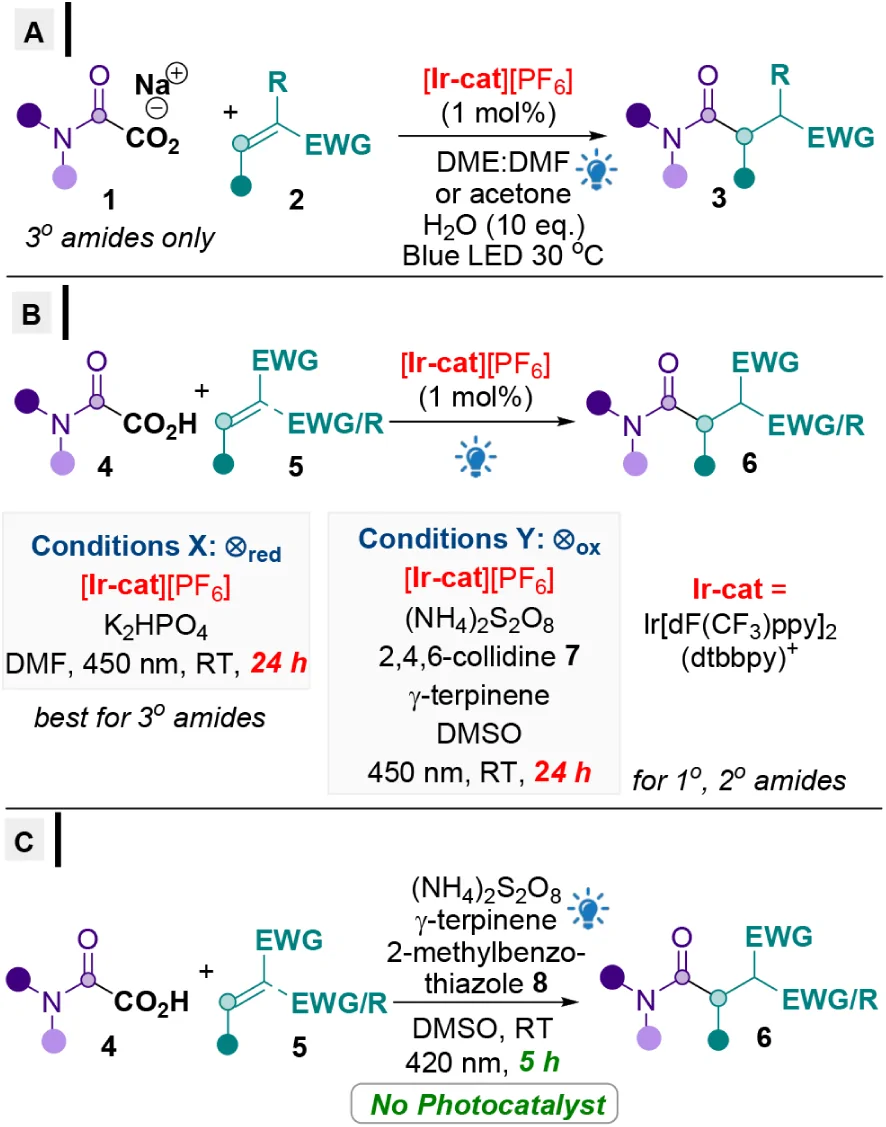
Direct, Decarboxylative Giese Amidations: (A), Previous Work, From Metal Oxamates (Kerr); (B), Previous Work, From Oxamic Acids (Lee); (C), This Work, No Photocatalyst, via EDA

One current limitation is that all these previous visible-light-mediated approaches to direct decarboxylative Giese amidation reactions rely on the use of a costly, potentially toxic, and nonsustainable^15^ Ir-based photocatalyst.^11,12,14^ Another major limitation is that previous attempts at scaling up Giese amidation reactions have resulted in reduced yields upon scale-up.^9,10,12,16^ In order to address both these limitations, we herein report the development of a direct decarboxylative Giese amidation reaction that instead exploits an *in situ*-formed electron donor–acceptor (EDA)^17^ complex, thereby negating the need for an exogenous photocatalyst. We also report an efficient scale-up of a Giese amidation reaction to gram scale by using a flow setup.

During the development of the previous work shown in Scheme 1B, we were convinced that a photocatalyst was necessary for the reaction, since a previous control reaction for Conditions Y (Scheme 1B) showed that the removal of the Ir-based photocatalyst was detrimental to the yield (17% vs 92% yield without and with the Ir-based photocatalyst).^12,18^ However, while developing Minisci amidations of 1,3-azoles,^19^ we serendipitously discovered that EDA formation between 1,3-azoles and persulfate (**EDA I**) can potentially allow for the breakdown of persulfate without the need for a photocatalyst in the light-mediated reaction (Scheme 2).^20^ We thus posited that the photochemical Giese amidation reaction under oxidative conditions (Conditions Y, Scheme 1B) can be rendered photocatalyst-free if the conditions can be altered to exploit an *in situ*-formed EDA between a suitable *N*-heterocyclic base and persulfate. In Scheme 1B, under Conditions Y, 2,4,6-collidine **7** was used as a base, so the plan was to alter the quantity and/or identity of the N-heterocyclic base as well as to ensure that the wavelength of irradiation is suitable for the EDA formed *in situ*.

**Scheme 2:**
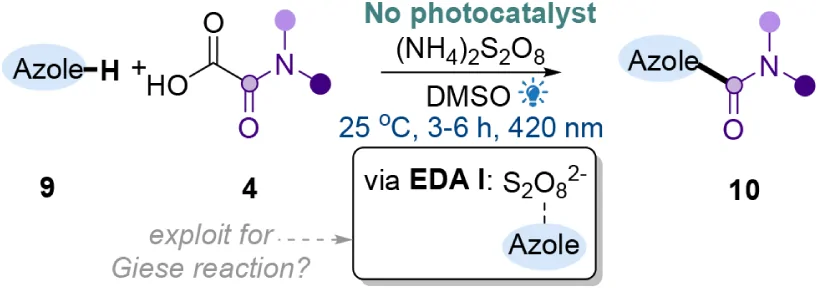
Suggested EDA Formation in Previous Minisci Amidations of 1,3-Azoles^20^

## Results and Discussion

We initiated our studies on the model system **4a** + **5a** → **6a**, using (NH_4_)_2_S_2_O_8_ as the oxidant,^21^ 2,4,6-collidine **7** as the base, and γ-terpinene as a hydrogen atom transfer (HAT) source (Table 1, see Supporting Information for full studies). First, as expected, simply removing the iridium photocatalyst under Conditions Y resulted in a poor 26% yield of **6a** (Entry 1). Since the *N*-heterocyclic base participates in any EDA complex formed, the amount of 2,4,6-collidine (**7**) was increased from 1 to 2 equiv, leading to a significant improvement in yield (Entry 2). Comparable yields were obtained under both 450 and 420 nm irradiation (Entries 2–3). Since 1,3-azoles were known to form suitable EDAs with persulfate (Scheme 2),^20^ 2-methylbenzothiazole **8** was next evaluated as a base.^22^ To our delight, this switch of base (from **7** to **8**) provided the optimal yield (Entry 5, Conditions A), although it should be noted that base **8** was more sensitive to the light source compared to **7**, and performed significantly better with 420 nm (Entry 5) than with 450 nm (Entry 4). Once again, reducing the equivalent of base **8** to 1 equiv was detrimental to the reaction (Entry 6). Time monitoring confirmed that 5 h were required for full conversion (Entries 7–9 vs Entry 5). Next, a solvent screen was carried out. While the reaction gave a decent 66% yield in CH_2_Cl_2_ (Entry 10), yields were noticeably poorer in MeCN, DMF, water, and acetone (Entries 11–14). Due to the toxicity of CH_2_Cl_2_, we opted to retain DMSO as the optimal solvent, as the use of DMSO also resulted in better yields (up to 88%).

**Table 1:**
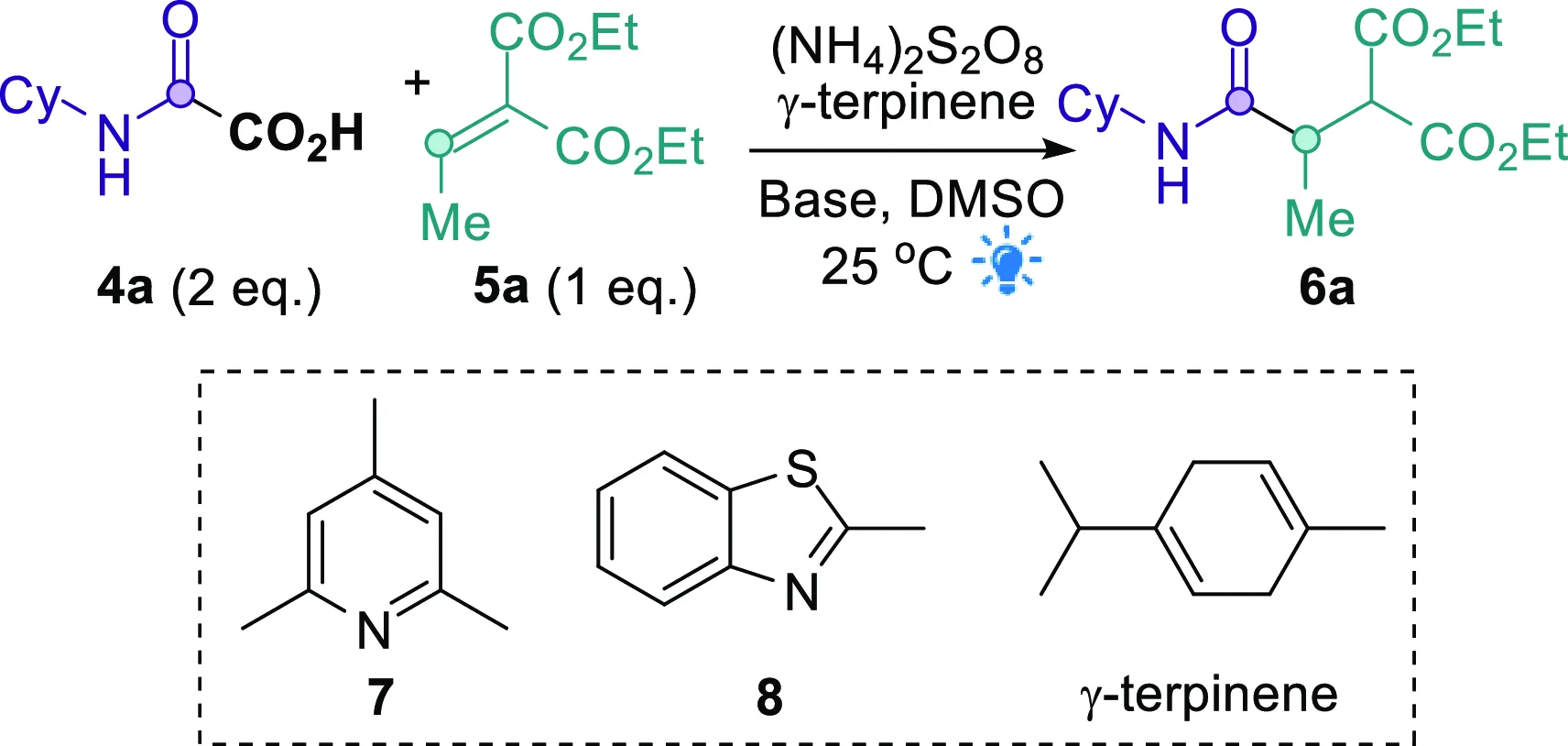

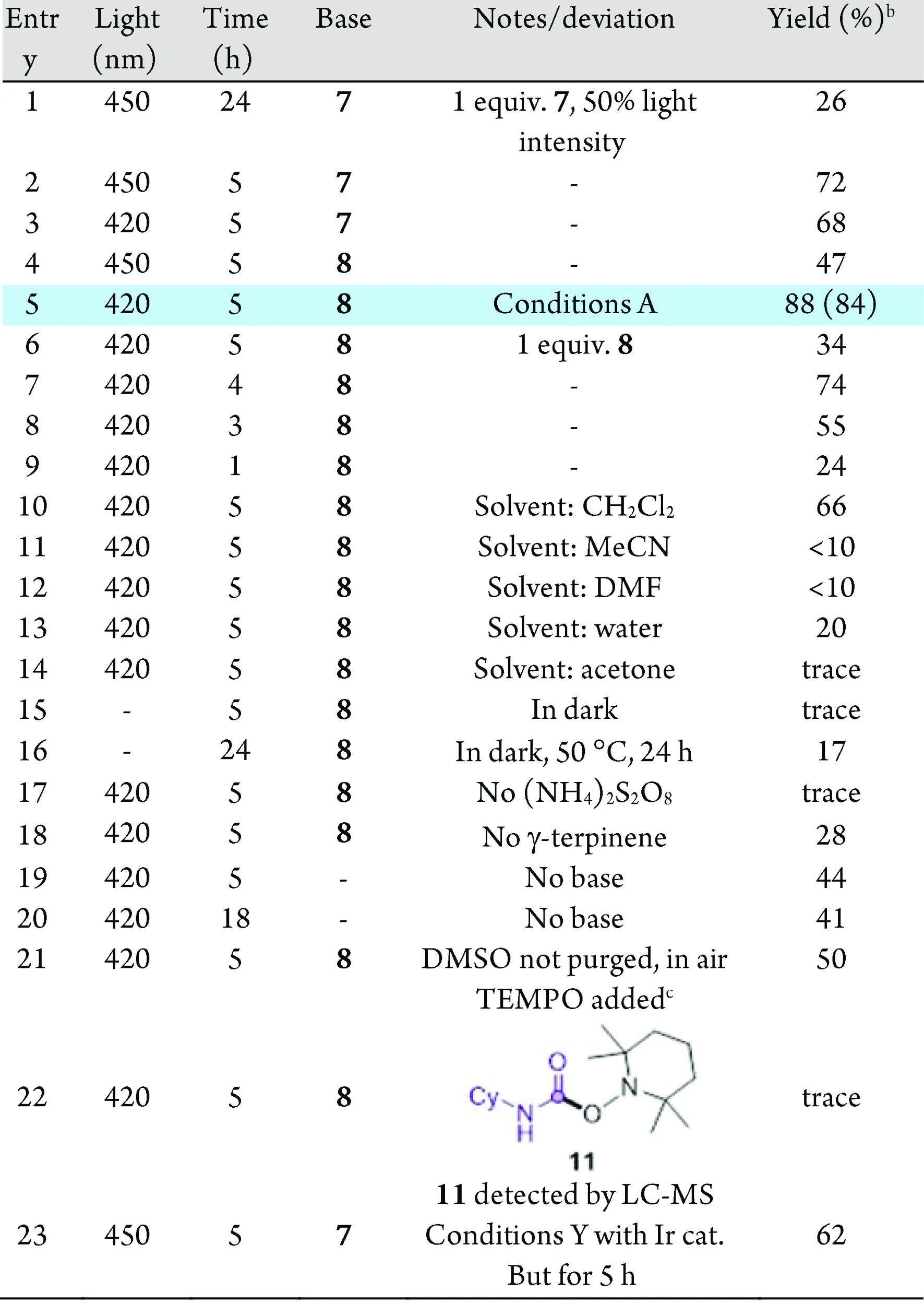
Optimization and Control Experiments^a^

a (NH_4_)_2_S_2_O_8_ (3 equiv), γ-terpinene (2 equiv), Base (2 equiv). Nonanhydrous DMSO purged with Ar or N_2_ prior to reaction. Penn PhD M2 Photoreactor, 100% light intensity.

b Determined by ^1^H NMR analysis of the crude mixture using CH_2_Br_2_ as an internal standard. Isolated yields are given in parentheses.

c 3 equiv.

Control reactions showed that light and persulfate are both necessary for reactivity (Entries 15–17) and yields are significantly poorer in the absence of the H atom source γ-terpinene (Entry 18) or base (Entries 19–20). Entry 21 confirmed that air was detrimental to the reaction yield. Finally, the addition of a radical scavenger, TEMPO (2,2,6,6-tetramethylpiperidine-1-oxyl), shuts down the Giese reaction (Entry 22), indicating a radical mechanism. Furthermore, the TEMPO-trapped carbamoyl intermediate **11** is detected by LC-MS. Although we previously showed that the reaction can be thermally mediated at 50 °C using base **7**,^12^ it should be noted that the result in Entry 15 indicates that the reaction is not thermally mediated under these light-mediated conditions reported here, which are run at a milder 25 °C. Entry 16 further shows that the reaction does not proceed well under thermal mediation when base **8** is used. The advantage of having a milder, complementary light-mediated vs thermally mediated reaction is that the former can provide better selectivity when sensitive substrates are used.^20^

Since the previous photocatalytic Conditions Y had a 24 h reaction time (Scheme 1B), the reaction was repeated for 5 h for a direct comparison of the efficiency of Conditions Y (with an Ir-based photocatalyst) vs A (without a photocatalyst). The photocatalytic reaction gave only 62% of **6a** vs 88% under Conditions A (Entry 23 vs Entry 5), confirming that our new photocatalyst-free protocol was more efficient, at least for this model reaction.

Therefore, a change in the *N*-heterocyclic base (from **7** to **8**), the equivalents of base (from 1 to 2 equiv), and the light irradiation wavelength (from 450 to 420 nm) to optimize conditions for potential EDA formation allows for photocatalyst-free conditions, removing the need for the costly and nonsustainable Ir-based photocatalyst.

With optimal conditions in hand (Conditions A), the oxamic acid **4** scope was explored and the yields were compared with the previously reported photocatalytic Conditions Y (Scheme 3). To our delight, the yields for installing secondary amides (**6a**–**i**) using photocatalyst-free Conditions A were generally competitive with the photocatalytic Conditions Y. Replacing the Cy ring in **6a** with cyclopentyl (**6b**, 78%), cycloheptyl (**6c**, 78%), or 1-Ad (**6d**, 72%) gives good yields of the expected products. Similarly, secondary amides with acyclic *N*-substituents produced good yields of the desired products (**6e** 73%, **6f** 83%). *N*-Aryl substituents were tolerated (**6g**–**i**), with electron-rich aryl-substituted oxamic acid **4** reacting more efficiently (**6h**, 55%) than their electron-poor aryl counterpart (**6i**, 43%, ∼85% purity), reflecting the reactivity of the respective nucleophilic radicals. A primary amide was also successfully added to give **6j** in 62% yield. Since it was known that the formation of tertiary amidated products is poor under oxidative conditions due to the oxidative formation of dealkylation products (e.g., **12**),^12^ it came as no surprise that the reaction was also poor yielding for the desired compound **6k** (11%) under Conditions A. Thus, as expected, the photocatalyst-free Conditions A are the best for installing primary and secondary amides, while the complementary photocatalytic reductive quenching Conditions (X, Scheme 1B) are still best for tertiary amide installation.^12^

**Scheme 3:**
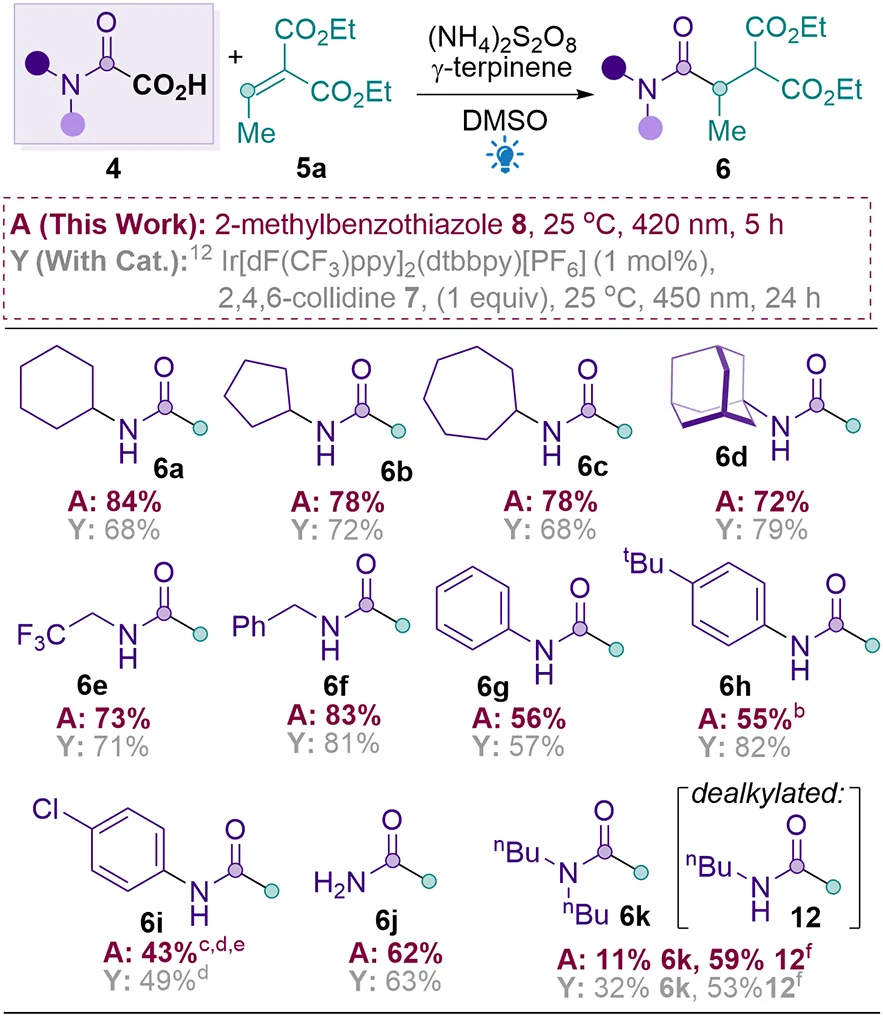
Oxamic Acid Scope Acid Scope^a^

a Isolated yields reported unless otherwise stated. ∼0.12 mmol **5**, (NH_4_)_2_S_2_O_8_ (∼3 equiv), γ-terpinene (∼2 equiv). For A: **8** (∼2 equiv). ^b^By ^1^H NMR using CH_2_Br_2_ as an internal standard due to coelution with the homocoupled product ArNHCOCONHAr. ^c^ Lower yields due to lower conversions. ^d^Fan speed at the lowest setting (2800 rpm). ^e^Approximately 85% purity. ^f^By ^1^H NMR using CH_2_Br_2_ as an internal standard.

The Michael acceptor **5** scope was investigated next (Scheme 4). Various acyclic acceptors reacted smoothly, such as diethyl maleate and diethyl fumarate, producing product **6l** in 65% and 79% yields, respectively. The electron-withdrawing groups (EWGs) need not be carbonyls; vinyl sulfone and diethyl vinylphosphonate were also good substrates, furnishing **6m** and **6n** in 76% and 74% yields, respectively. A variety of cyclic acceptors with different ring sizes and EWGs such as cyclohexanone (**6o**, 70%), cyclopentenone (**6p**, 69%), cycloheptanone (**6q**, 76%), butenolide (**6r**, 74%), pentenolide (**6s**, 46%, ∼90% purity), and α,β-unsaturated amide (**6t**, 52%) were also suitable substrates.

**Scheme 4:**
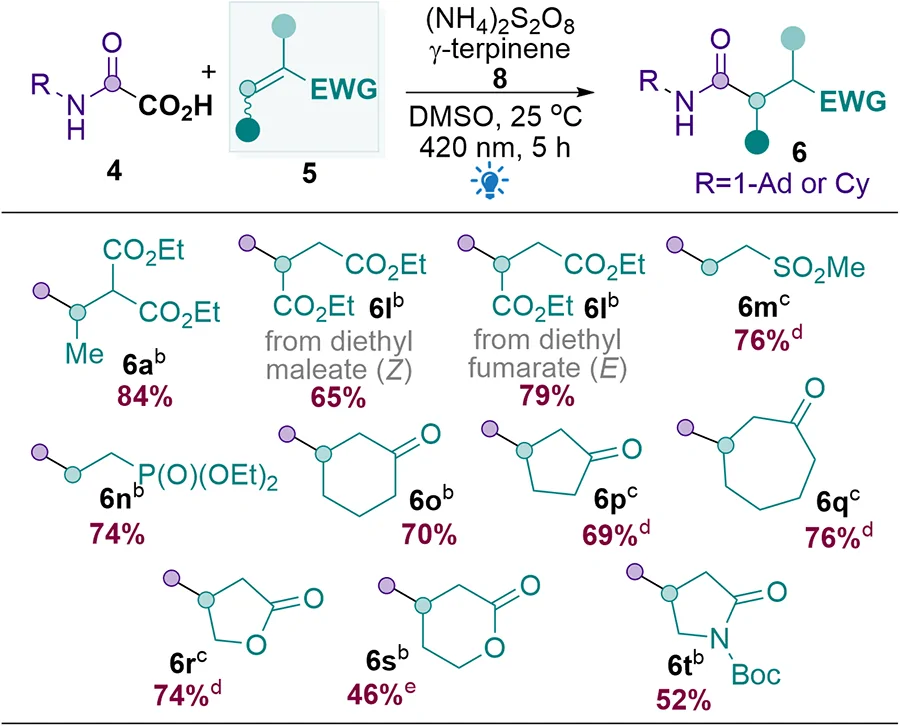
Michael Acceptor Scope^a^

a Isolated yields unless otherwise stated. ∼0.12 mmol **5**, ∼0.24 mmol **4**, (NH_4_)_2_S_2_O_8_ (∼3 equiv), γ-terpinene (∼2 equiv), 2-methylbenzothiazole **8** (∼2 equiv). ^b^R = Cy, ^c^R = 1-Ad. ^d^0.25 mmol **4**. ^e^Approximately 90% purity.

The scale-up of the reaction was investigated next, in both batch and flow (Scheme 5). As expected, according to the Beer–Lambert law, scaling up in batch causes the reaction to be less efficient due to the increased irradiation path length,^23^ with a drop in yield evident upon scaling up from 0.15 mmol (84%) to 3.91 mmol (72%) even upon extending the reaction time (24 h). For this reason, we investigated the reaction in flow, where small reactor channel dimensions reduce the path length of the light required to totally irradiate the reaction solution, thus typically providing more efficient scale-up for light-mediated reactions.^17c,23^ Initially, the scale-up to 0.48 and 1.18 mmol under the standard batch reaction time of 5 h gave a 72% yield of **6a** (Scheme 5). Time monitoring of the reaction in flow at a 1.44 mmol scale (see Supporting Information, Figure S8) showed that the reaction continued to react over time and that a 24 h reaction time was a more suitable duration to give a good 87% yield of **6a**. To our delight, the 3.97 mmol reaction in flow successfully gave 1.1 g (88%) of product **6a**, which was a marked improvement in efficiency over the batch reaction on the same time scale (0.88 g, 72%).

**Scheme 5:**
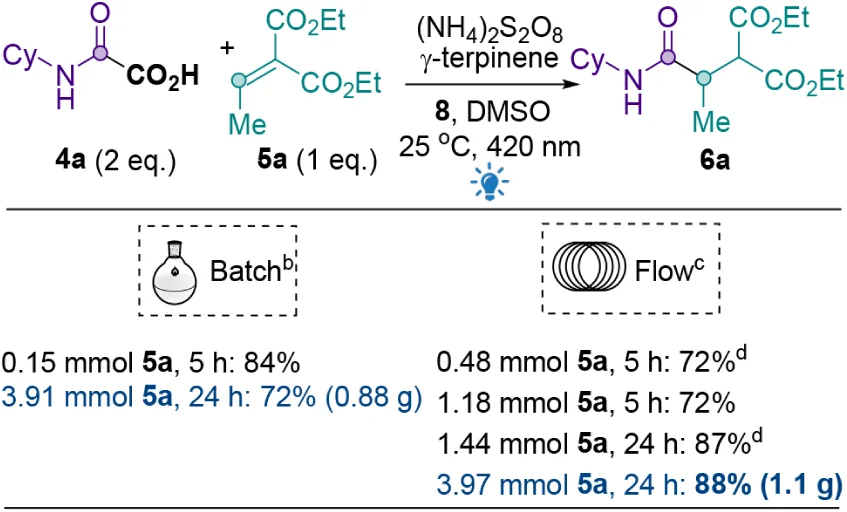
Scale-Up in Batch and Flow^a^

a Isolated yields of **6a** unless otherwise stated. (NH_4_)_2_S_2_O_8_ (∼3 equiv), γ-terpinene (∼2 equiv), **8** (∼2 equiv), ^b^Phen PhD M2 Photoreactor. ^c^Vaportec E-Series flow system, 10 mL reactor volume. See Supporting Information for flow rates and residency times. ^d^By ^1^H NMR using CH_2_Br_2_ as the internal standard.

We were particularly pleased with the successful and efficient scale-up of the reaction to gram scale under flow conditions, with no drop in yield upon scale-up. This is because all reported attempts at scaling up the Giese amidation reaction (even reactions that are not light-mediated)^12^ have so far resulted in lower yields, noting particular sensitivity to reaction scale.^9,10,12,16^ For example, a thermally mediated batch protocol exhibited a large drop in yield upon scale-up [81% (0.12 mmol) to 72% (1 mmol) and 55% (5.4 mmol)] for **6f**.^12^

Finally, we demonstrated that the flow procedure can be used on more complex molecules such as the oxamic acid **4** derived from the natural product leelamine, to give **6u** (1:1 dr) in 78% yield (Scheme 6). A common bicyclic chiral building block^24^ was also successfully used as a Michael acceptor to give **6v** in a 65% yield. In order to challenge the system further, we attempted to combine the complex amine with a complex Michael acceptor, and **6w** was successfully produced in a 65% yield.

**Scheme 6:**
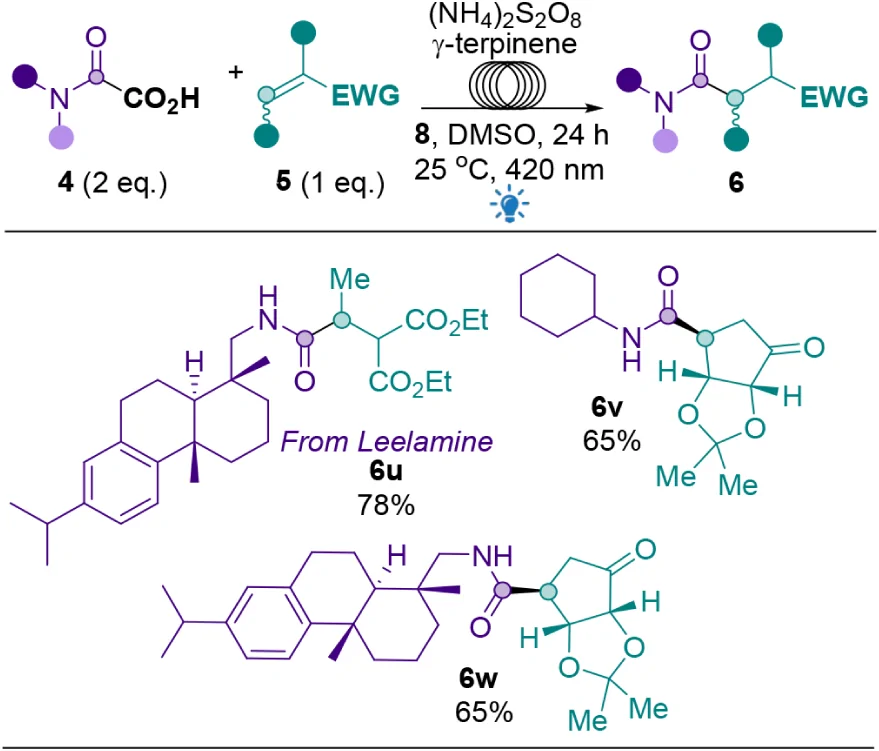
Application to Natural Products and Chiral Building Blocks in Flow^a^

a Isolated yields. 0.30 mmol scale of **5**, (NH4)_2_S_2_O_8_ (∼3 equiv), γ-terpinene (∼2 equiv), **8** (∼2 equiv). Vapourtec E-Series flow system. Flow rate: 1.1 mL/min, 10 mL reactor volume, residency time: 9.09 min.

Under photocatalytic Conditions Y, the Ir-based photocatalyst is thought to be instrumental in the breakdown of persulfate S_2_O_8_^2–^ to the active oxidant, radical anion SO_4_^–^ **˙ II**, as well as to catalyze the SET between **I** and **III** (Scheme 7A).^12^ For Conditions A, UV–vis absorption studies were carried out in order to ascertain the active visible light-absorbing species (Figure 1) in the absence of a photocatalyst. Neither (NH_4_)_2_S_2_O_8_ nor 2-methylbenzothiazole **8** absorbs at the reaction wavelength of 420 nm (Figure 1) and these species form colorless solutions (Figure 2). Mixing (NH_4_)_2_S_2_O_8_ with **8**, however, results in a color change from colorless to yellow (Figure 2), a bathochromic shift, and increased absorption, consistent with the formation of an EDA (**EDA II**, Scheme 7B).^17^ While we note that the combination of **4a** + **8** is also very weakly absorbing at 420 nm, this combination does not result in a color change or any appreciable bathochromic shift or increased absorption that would be indicative of EDA formation. Thus, since the combination of (NH_4_)_2_S_2_O_8_ with **8** is also the strongest absorbing mixture at 420 nm (Figure 1) and it is only when (NH_4_)_2_S_2_O_8_ and **8** are present together that any color change is observed (Figure 2 and Supporting Information), we expect this to be consistent with a mechanism via **EDA II** dominating in the presence of **8**.

**Scheme 7:**
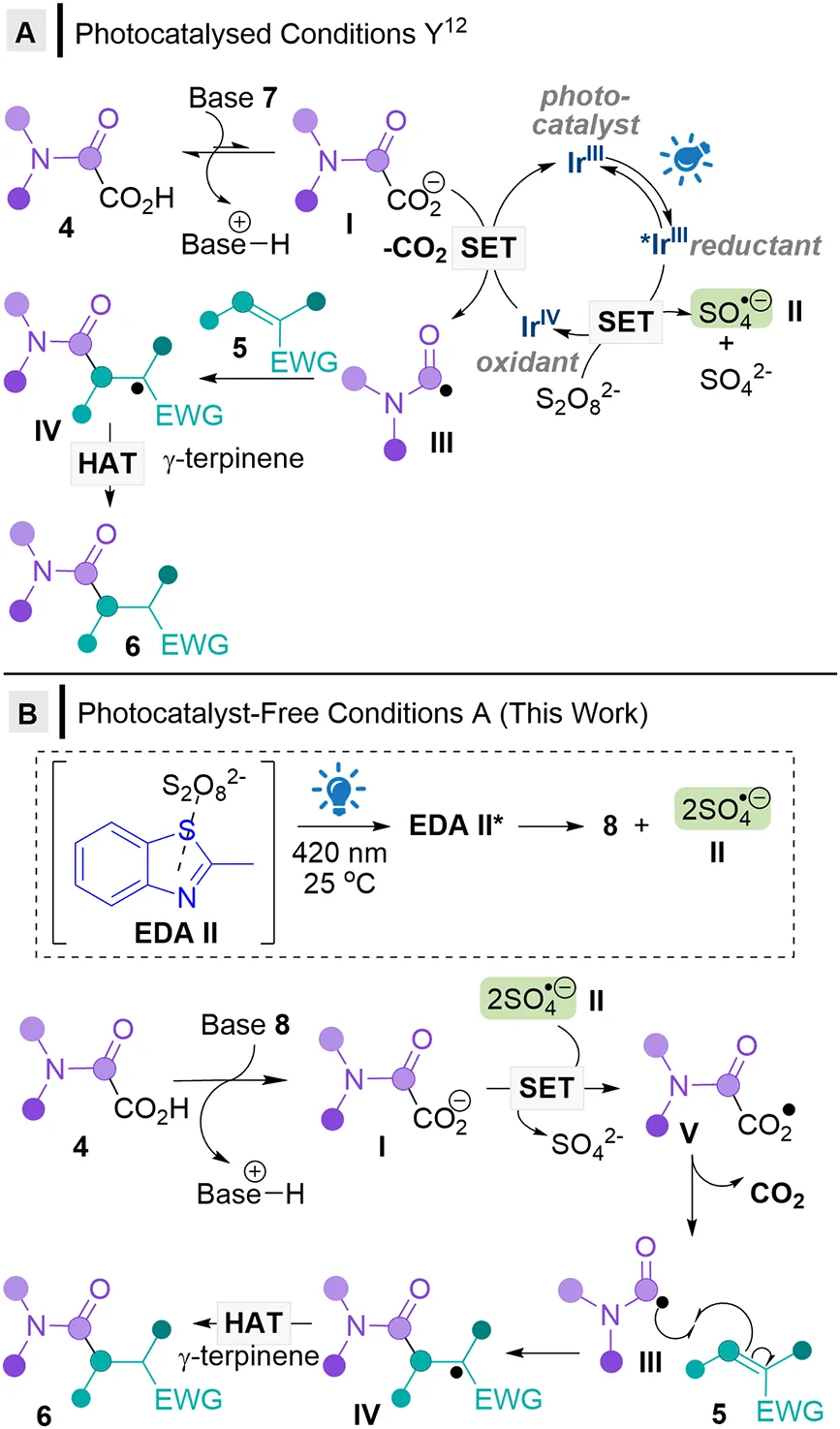
Plausible Mechanism^12^

**Figure 1:**
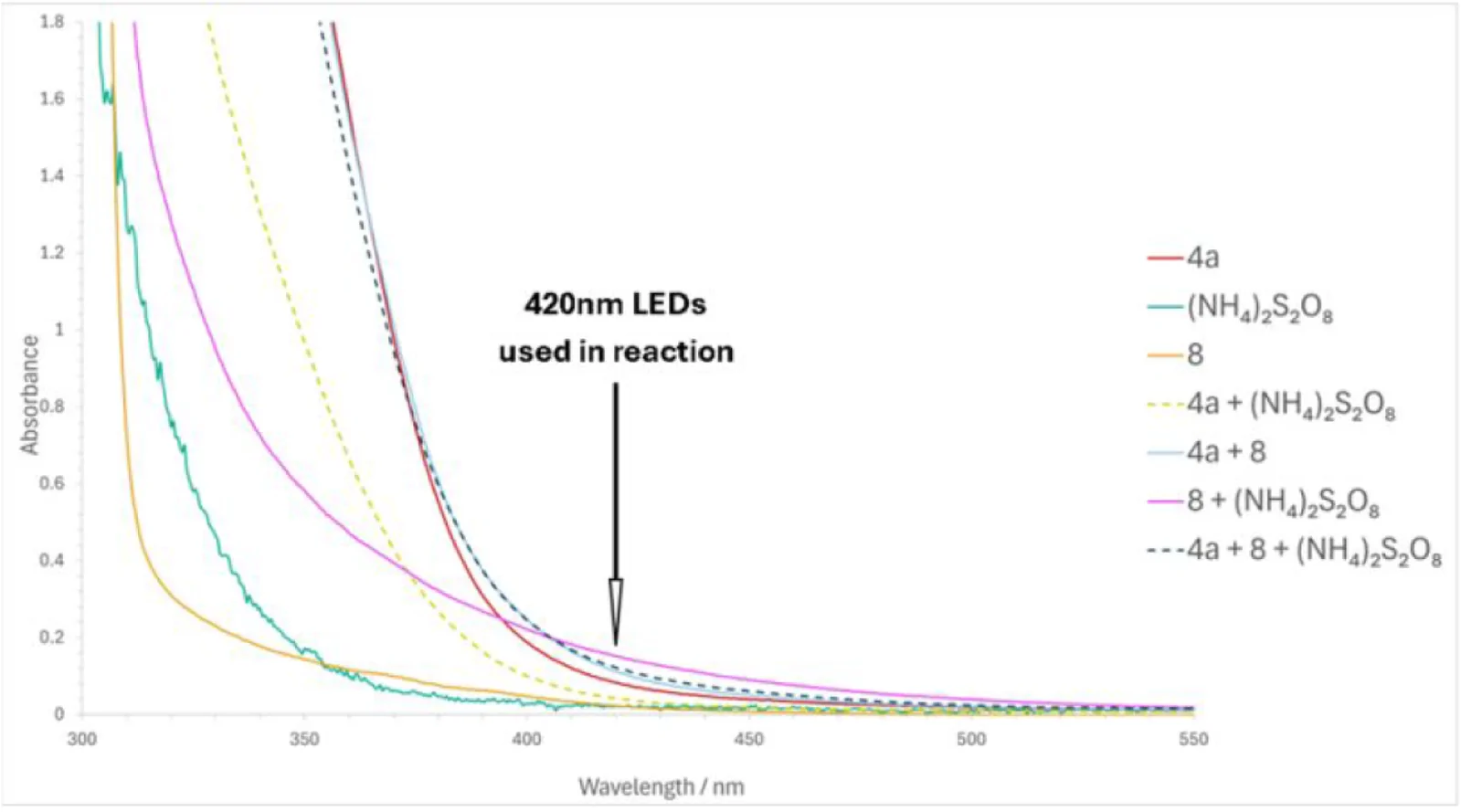
Selected UV–vis absorption studies.

**Figure 2:**
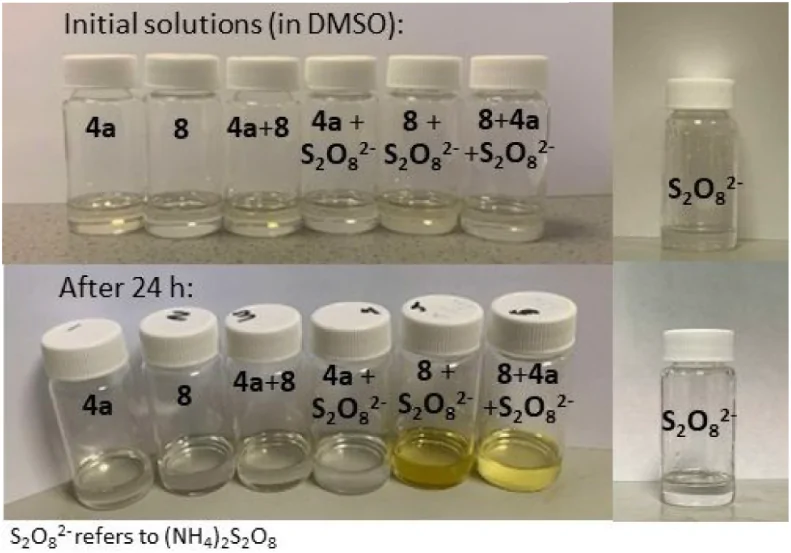
Observed color change.

Our proposed mechanism for Conditions A is shown in Scheme 7B. We previously showed that the Job’s plot analyses^25^ for combinations of various 1,3-azoles with persulfate gave maximum absorptions that were *not* at a 1:1 ratio, thus implying that the reaction did *not* proceed via a SET from donor to acceptor of the EDA.^20a^ While photodecomposition of S_2_O_8_^2–^ by itself will not occur under visible light (UV light required, Figure 1), we propose that excitation of *in situ*-formed **EDA II** can facilitate homolytic decomposition of S_2_O_8_^2–^ at 420 nm.^21d,26^ SET between **I** and **II** followed by decarboxylation^27^ gives the nucleophilic carbamoyl radical **III**,^13e,28^ which subsequently adds to Michael acceptor **5** to form **IV**. HAT between **IV** and γ-terpinene furnishes the desired Giese product **6**. The average quantum yield (Φ) is 0.0110 (std. dev. 0.00148), implying that a radical chain propagation mechanism does not occur.^29^

## Conclusions

In conclusion, a photocatalyst-free, light-mediated direct decarboxylative Giese amidation reaction has been successfully developed via a pathway that is thought to involve *in situ* EDA formation. Judicious choice of an N-heterocyclic base as well as irradiation wavelength was a key factor that allowed for an efficient reaction in the absence of a photocatalyst. This photocatalyst-free procedure removes the costly and nonsustainable Ir-based photocatalyst, as well as any toxicity issues associated with the use of metal-based photocatalysts to give a competitive and practically attractive alternative to the photocatalyzed counterparts. These oxidative conditions, as with their photocatalyzed counterpart (Cond. Y), are suitable for a range of cyclic and acyclic Michael acceptors, as well as for primary and secondary amidations. The reaction was also successfully scaled up to the gram scale, with flow conditions outperforming batch conditions for efficiency of scale-up. Our successful and efficient scale-up is noteworthy as previously reported attempts at scaling up Giese amidation reactions have noted particular sensitivity to reaction scale.

## Supplementary Material



## Data Availability

The data underlying this study are available in the published article, in its Supporting Information, and are openly available at doi.org/10.17861/f52bb21c-9082–4aba-9b24–3f6410e1030d.

## Notes

Optimization studies, all experimental details, characterization, and copies of NMR data (PDF)
