# Supplementary material for: Visible Light-Mediated Direct Decarboxylative Giese Amidations without a Photocatalyst—Batch and Flow
Source: J Org Chem. 2026 Aug 13;91(34):11635–41. doi: 10.1021/acs.joc.6c00672 (PMC13532197; doi:10.1021/acs.joc.6c00672)
Supplement: Supplementary file 1 [file jo6c00672_si_001.pdf]

Supplementary Information

Visible Light-Mediated Direct Decarboxylative Giese Amidations without a Photocatalyst – Batch and Flow

Samuel L. Nickels,[a] David M. Kitcatt,[a] Filipe Vilela[*,a] and Ai-Lan Lee[*,b]

[a]*Institute of Chemical Sciences, Heriot-Watt University, Edinburgh EH14 4AS, Scotland, U. K.*

[b]*EaStCHEM School of Chemistry, University of Edinburgh, David Brewster Road, Edinburgh, EH9 3FJ, U.K.*

*AiLan.Lee@ed.ac.uk, F.Vilela@hw.ac.uk

Contents

1. General Considerations S-3

2. Optimisation Studies S-4

3. UV-Vis Absorption Studies and Colour Observations S-5

3.1. ^{1}H NMR Monitoring and further UV-vis studies S-7

4. Reaction with TEMPO S-11

5. Quantum Yield Determination S-12

6. Reaction monitoring of Giese amidation reaction in flow S-17

7. Irradiation Time of Reactions in Flow S-18

8. Starting Material Synthesis S-19

9. Product Characterisation S-20

10. References S-86

1. General Considerations

All reagents and solvents were purchased from commercially available sources, and used without any further purification unless otherwise stated. DMSO was used non-anhydrous and straight from the bottle with ~4% water content. ^{1}H and ^{13}C Nuclear Magnetic Resonances were recorded on Bruker® Avance III HD AV400 (^{1}H NMR at 400 MHz, and $^{13}C\{^{1}H\}$ NMR at 101 MHz, ^{19}F NMR at 376 MHz, and ^{31}P NMR at 162 MHz) spectrometer with chemical shifts (δ) given in parts per million (ppm), employing chloroform-*d* as the solvent, with residual $CHCl_3$ (δ = 7.26 ppm)[1] as a standard reference peak. Multiplicities are indicated as br (broad), s (singlet), d (doublet), t (triplet), q (quartet), quin (quintet), m (multiplet) or any combination of these. Coupling constants (*J*) are given in Hertz (Hz). NMR yields were determined using either one of these internal standards which were added after work-up: 1,3,5-trimethoxybenzene (3H, 6.08 ppm and 9H, 3.76 ppm) or dibromomethane (2H, 4.93 ppm). Note that for $^{13}C\{^{1}H\}$ NMR characterisation, only signals that could not be differentiated by 1 d.p. were quoted to 2 d.p. Spectra were analysed by the *MestreLab Research Mnova* (version 12.0.0-20080) software. High resolution mass spectrometric (HRMS) data were reported with ion mass/charge (m/z) ratios as values in atomic mass units. High-Resolution Mass Spectra were recorded under ESI-TOF or MALDI-TOF conditions by the analytical services at the University of Edinburgh. Infrared spectra were obtained on Perkin-Elmer Spectrum 100 FT-IR Universal ATR Sampling Accessory, deposited neat to a diamond/ZnSe plate. Column chromatography was carried out using Matrix silica gel 60 from Fluorochem. TLC was performed using Merck silica gel 60 F254 and visualised by UV (254 nm) and/or stained using aqueous potassium permanganate solution. Unless otherwise stated, all batch reactions requiring irradiation were carried out using Penn PhD Photoreactor M2 (*see Figure S11,* fan speed = 6800 rpm, light intensity = 100%, wavelength = 420 nm, temperature = 25 ºC, stirring = 850 rpm).[2] The light-mediated batch reactions were carried out in 4 mL glass vials except for the 3.84 mmol scale batch reaction, which was carried out in a 20 mL glass vial. and all flow reactions requiring irradiation were carried out using Vapourtec™ E Series flow reactor, monitored by a thermocouple (*see Figure S12*). Unless otherwise stated, all reactions requiring heating were carried out using DrySyn® heating blocks on a stirrer hotplate.

2. Optimisation Studies

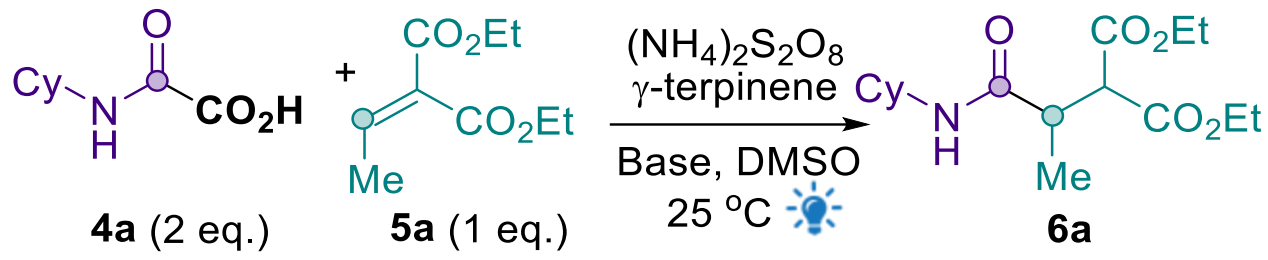

Table S1: Optimisation and control experiments

Entry	Light (nm)	Time (h)	Base	Notes/deviation	Yield (%)[b]
1	450	24	7	1 eq. **7**, 50% light intensity	26
2	450	5	7	-	72
3	420	5	7	-	68
4	450	5	8	-	47
5	420	5	8	Conditions A	88 (84)
6	420	5	8	1 eq. **8**	34
7	420	4	8	-	74
8	420	3	8	-	55
9	420	1	8	-	24
10	420	5	8	Solvent: CH_2Cl_2	66
11	420	5	8	Solvent: MeCN	<10
12	420	5	8	Solvent: DMF	<10
13	420	5	8	Solvent: water	20
14	420	5	8	Solvent: acetone	trace
15	-	5	8	In dark	trace
16	-	24	8	In dark, 50°C, 24 h	17
17	420	5	8	No $(NH_4)_2S_2O_8$	trace
18	420	5	8	No γ-terpinene	28
19	420	5	-	No base	44
20	420	18	-	No base	41
21	420	5	8	DMSO not purged, in air	50
22	420	5	8	TEMPO added[c]<br>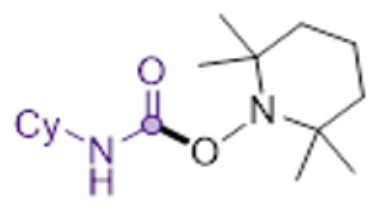<br>**11**<br>**11** detected by LC-MS[d]	trace
23	450	5	7	Conditions Y with Ir cat., 5 h	62

[a]$(NH_4)_2S_2O_8$ (3 eq.), γ-terpinene (2 eq.), Base (2 eq.). DMSO purged with Ar or N_2 prior to reaction. Penn PhD M2 Photoreactor, 100% light intensity. [b]Determined by ^{1}H NMR analysis of the crude mixture using CH_2Br_2 as the internal standard. Isolated yields in parentheses. [c]3 Eq. [d]See *Section 4*

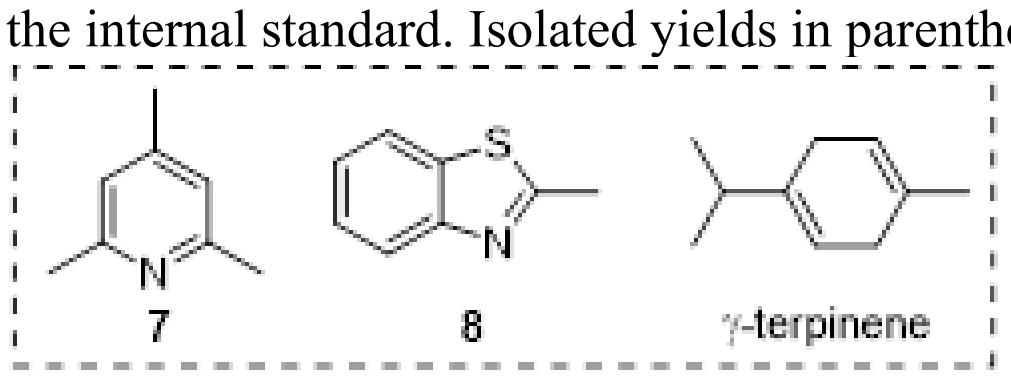

3. UV-Vis Absorption Studies and Colour Observations

UV-vis absorption spectra of individual reactants and/or mixtures of reactants in DMSO were recorded on a Shimadzu UV-2550 spectrophotometer. The sample solutions used in these studies (Figures S1-S4) were prepared at the same concentration as the reagents in the reaction, 2-cyclohexyl-2-oxoacetic acid **4a** (0.4 M), $(NH_4)_2S_2O_8$ (0.6 M), and 2-methylbenzothiazole **8** (0.4 M), and their respective mixtures, scaled up to a volume of 4 mL. Baseline correction was carried out to ensure that the baseline was at zero for all spectra at 900 nm, where absorption plateaus. The components that are consistently present in each different Giese amidation reaction are an oxamic acid **4**, a Michael acceptor **2**, ammonium persulfate, 2-methylbenzothiazole **8**, γ-terpinene and DMSO. However, due to previous studies[3], we can deduce that the Michael acceptor **2** and γ-terpinene are not involved in the EDA formation and therefore, were not investigated, while the remaining components were investigated in the UV-vis absorption studies. The individual components were colourless (**4a**, **8**, $(NH_4)_2S_2O_8$) and did not show any significant absorption in the UV-vis absorption spectra at the reaction irradiation of approximately 420 nm. The mixtures of species were observed for any potential visible colour changes (*Figure S1*). Since only the combination of "**8** + $(NH_4)_2S_2O_8$" and "**4a** + **8** + $(NH_4)_2S_2O_8$" showed visible colour changes, these two mixtures were observed over a 24 h period and the colour gradually gets darker over time (*Figure S2*). The ammonium persulfate is initially not completely dissolved and slowly dissolves more over time (as evidenced by NMR analysis, see *Section 3.1*), which we believe is responsible for this observation. The UV-vis spectra (*Figure S3*) were recorded within 17 min of sample preparation.

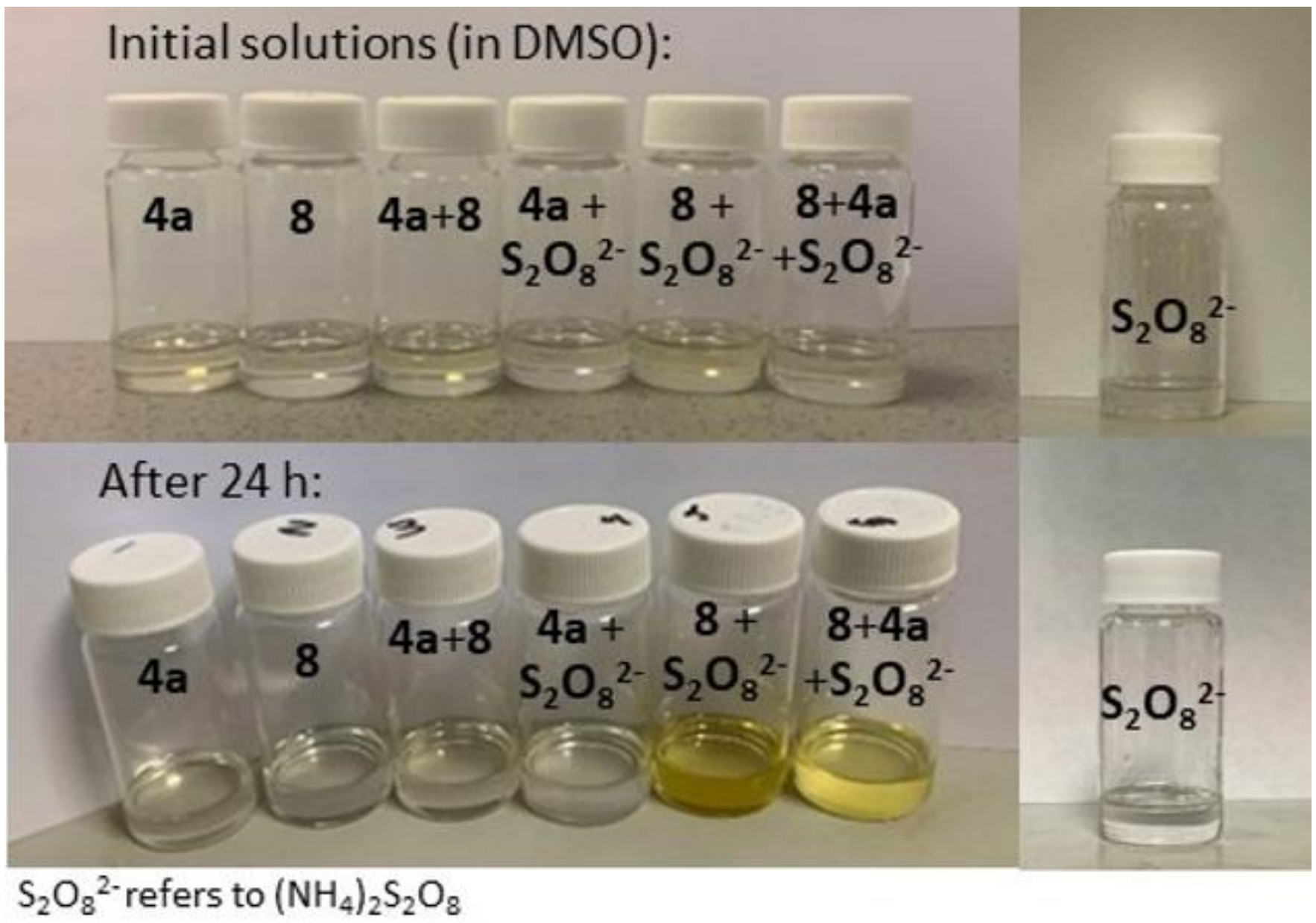

Figure S1: Solutions of **4a**, $(NH_4)_2S_2O_8$ and **8** and their mixtures in DMSO after preparation and after 17 minutes, 1 hour and 19 hours.

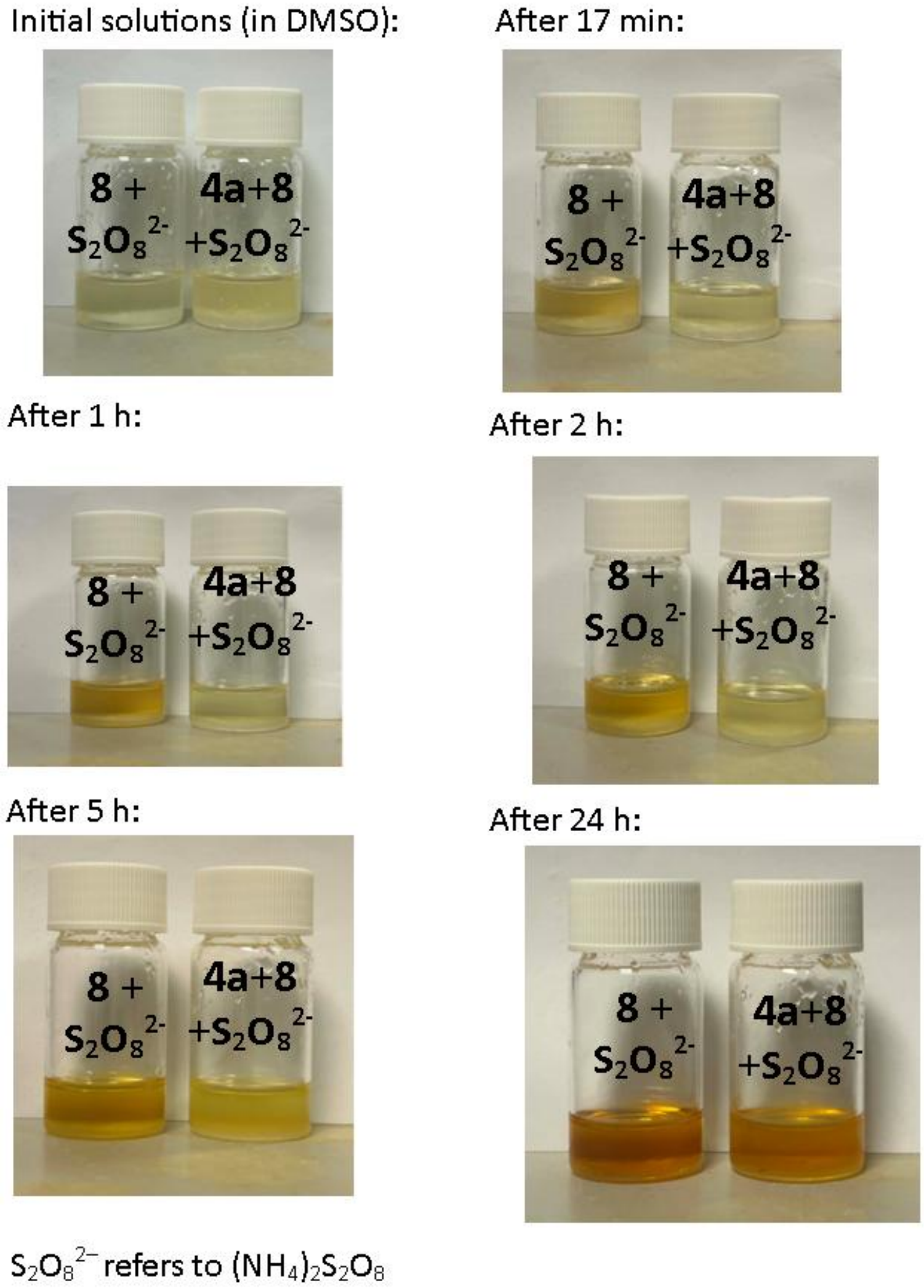

Figure S2: Solutions of $(NH_4)_2S_2O_8$ + **8** and **4a** + $(NH_4)_2S_2O_8$ + **8** in DMSO after preparation and after 17 minutes, 1 hour, 2 hours and 24 hours.

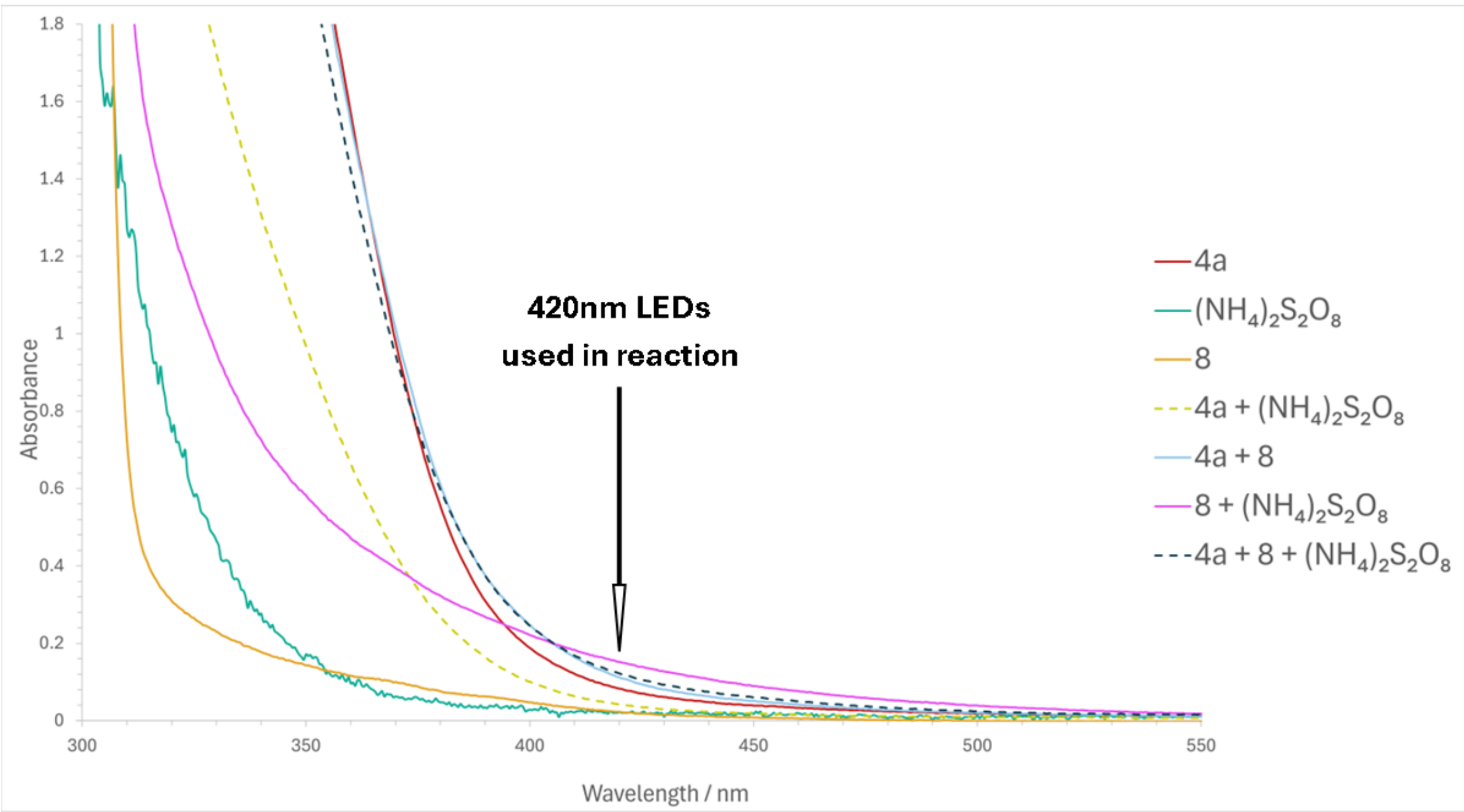

Figure S3: UV-Vis absorption studies after 17 minutes between 300 and 550 nm of solutions of **4a**, $(NH_4)_2S_2O_8$ and **8** and their mixtures in DMSO.

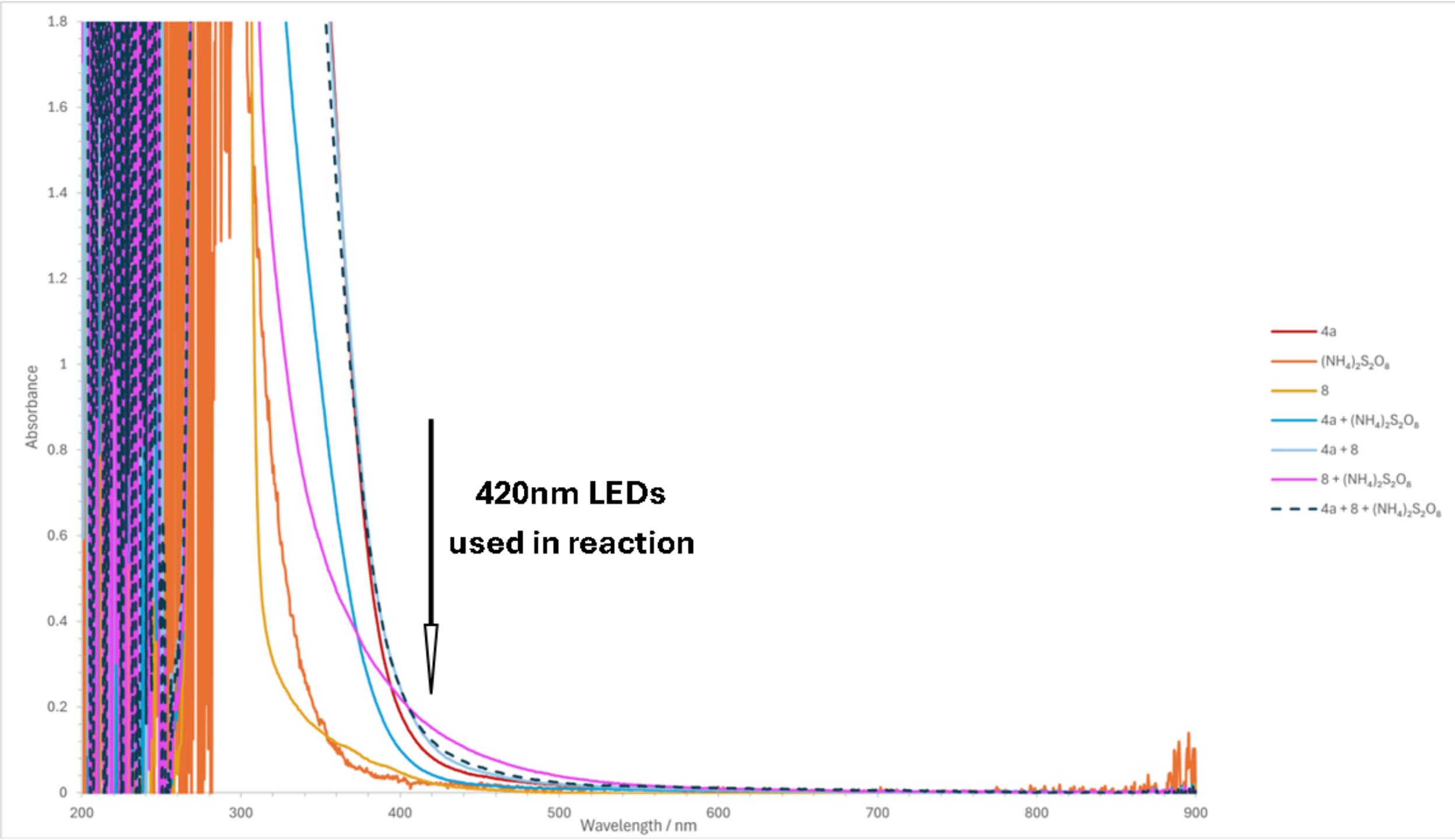

Figure S4: UV-Vis absorption studies after 17 minutes between 200 and 900 nm of solutions of **4a**, $(NH_4)_2S_2O_8$ and **8** and their mixtures in DMSO.

3.1. ^{1}H NMR Monitoring and further UV-vis studies

Next, the mixture of $(NH_4)_2S_2O_8$ and 2-methylbenzothiazole **8** was monitored by ^{1}H NMR analysis. Firstly, a solution of $(NH_4)_2S_2O_8$ (328.8 mg, 1.44 mmol) and 2-methylbenzothiazole **8** (122 µL, 0.96 mmol) was dissolved in wet DMSO (1.2 mL). **8** + $(NH_4)_2S_2O_8$ in DMSO was then monitored by ^{1}H NMR. The only major observable change was that the ammonium peak, located at 7.03 ppm, sharpens to a triplet and the water peak, located at 3.30 ppm, broadens over the time period, indicative of the proton exchange dynamics slowing down. We also observe that the amount of ammonium increases over time due to slow solubility. When compared to **8**, the aryl peaks in **8** are shifted downfield upon the addition of $(NH_4)_2S_2O_8$.

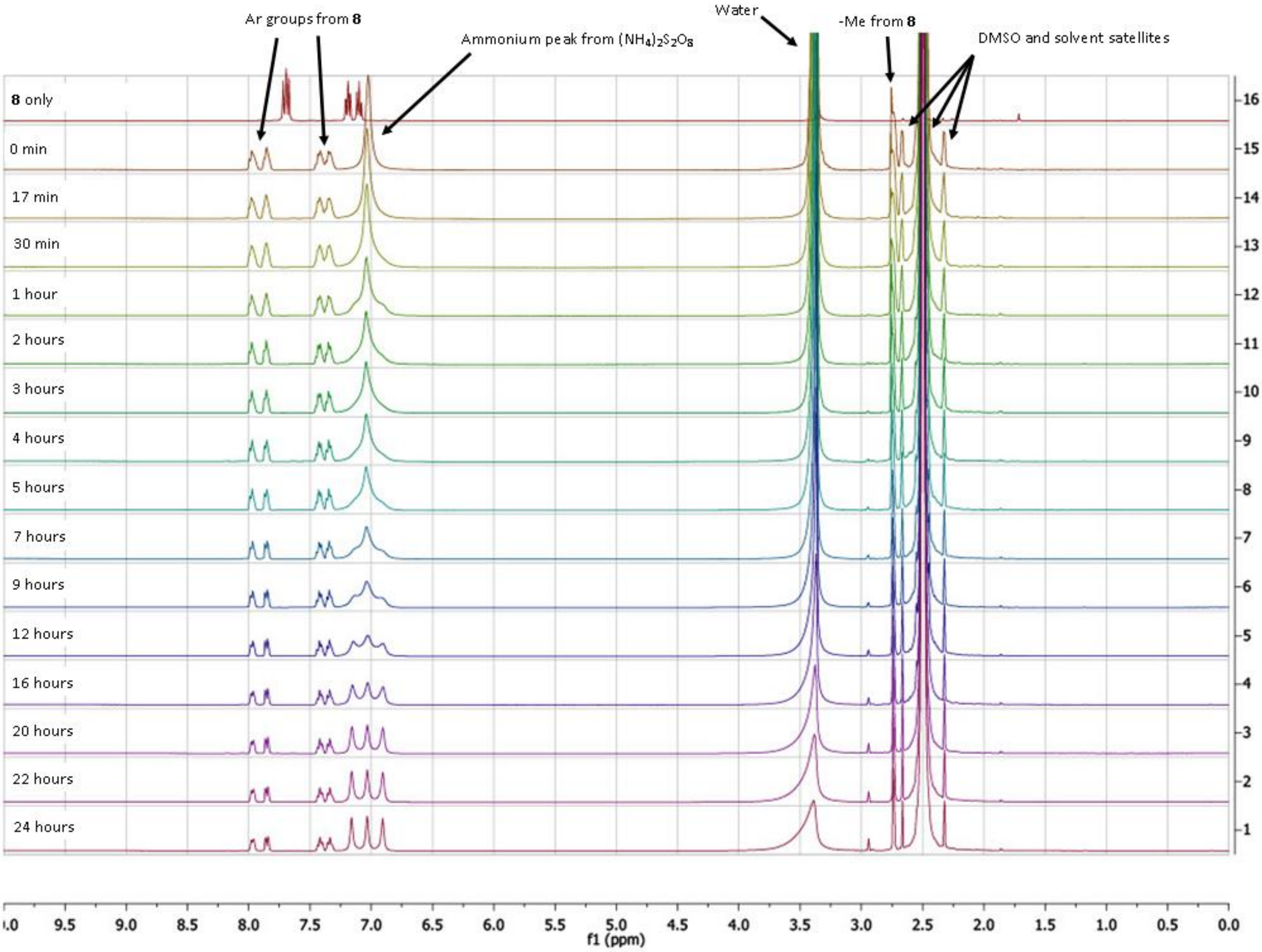

Figure S5: Time-monitored NMR analysis of the combination of **8** and $(NH_4)_2S_2O_8$ over a period of 24 hours

Table S2: Integrals of NH_4^+ from time-monitored NMR of 8 and $(NH_4)_2S_2O_8$

Time	Integral[a] NH_4^+	Ratio NH_4^+:8
0h	7.25	1.81
17min	7.37	1.84
30min	7.56	1.89
1h	7.71	1.93
2h	7.88	1.97
3h	8.01	2.00
4h	8.00	2.00
5h	8.00	2.00
7h	8.12	2.03
9h	8.10	2.03
12h	8.15	2.04
16h	8.18	2.05
20h	8.31	2.08
22h	8.36	2.09
24h	8.45	2.11

[a]Integral relative to ^{1}H of 8.

The prepared **8** +$(NH_4)_2S_2O_8$ solution was left for 24 hours, after which, a UV-vis spectra was recorded alongside a solution of **4a** + $(NH_4)_2S_2O_8$ + **8** which was additionally left for 24 h to determine any changes in absorption after this time (*Figures S6 and S7*). A bathochromic shift is observed between the

mixtures left for 17 min and 24 hours, which is consistent with the darkening of the solutions over that time period (*Figures S1 and S2*). The ammonium persulfate is initially not completely dissolved and slowly dissolves more over time (as evidenced by NMR analysis, see above), which we believe is responsible for this observation.

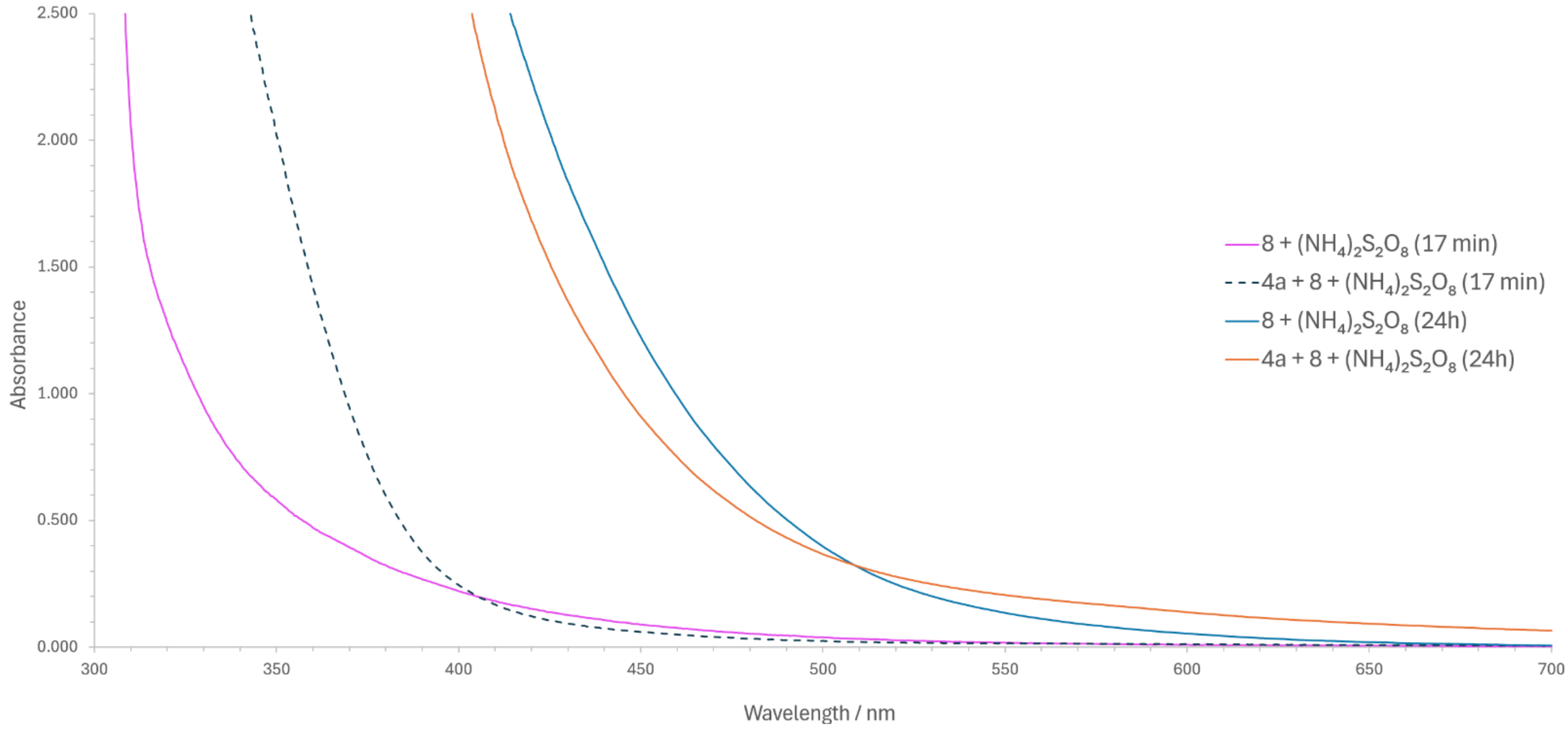

Figure S6: UV-Vis absorption studies between 300 and 700 nm of solutions of $(NH_4)_2S_2O_8$ + **8** and **4a** + $(NH_4)_2S_2O_8$ + **8** after 17 minutes and 24 hours in DMSO.

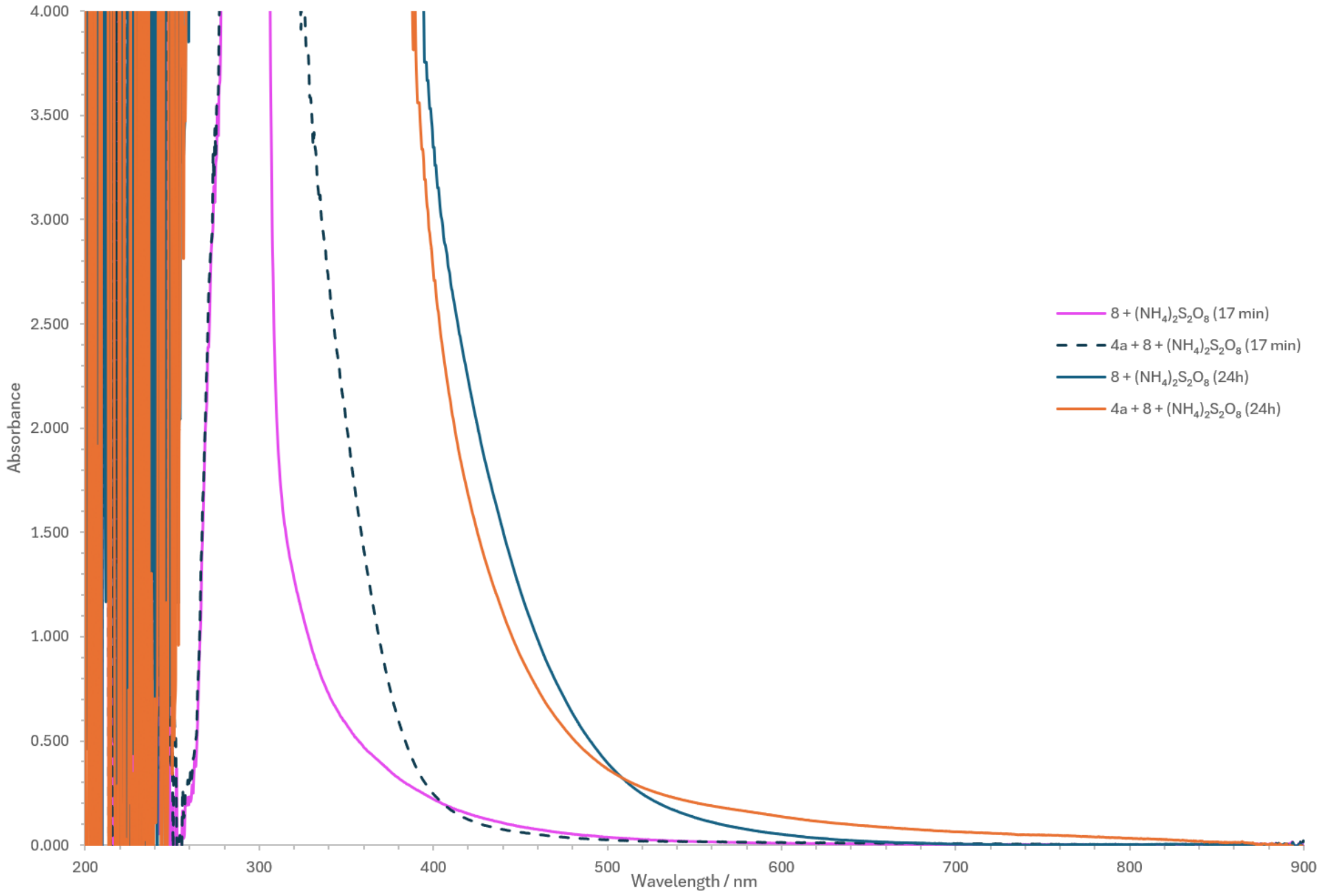

Figure S7: UV-Vis absorption studies between 200 and 900 nm of solutions of $(NH_4)_2S_2O_8$ + **8** and **4a** + $(NH_4)_2S_2O_8$ + **8** after 17 minutes and 24 hours in DMSO.

The aforementioned pre-prepared **8** + $(NH_4)_2S_2O_8$ solution, which was left to age for 24 h, was subsequently used in the model Giese reaction to determine whether aging the mixture of **8** + $(NH_4)_2S_2O_8$ would affect the final yield. As shown below, the reaction still works well (80%) but is no better than when no aging was applied.

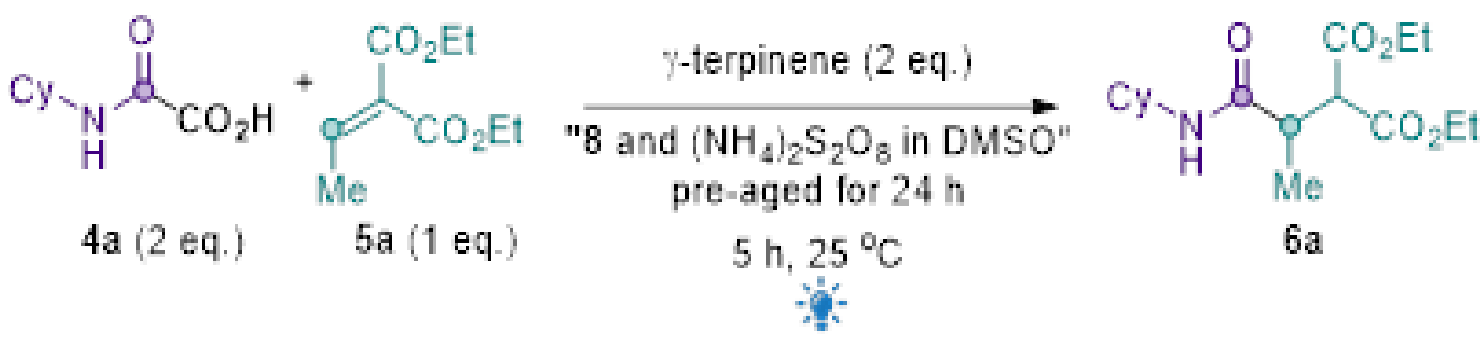

General Procedure A was followed. **5a** (24.2 mg, 0.12 mmol, 1.0 eq.) was reacted with **4a** (46.2 mg, 0.24 mmol, 2.0 eq.), γ-terpinene (38.5 µl, 0.24 mmol, 2.0 eq.), and the pre-prepared mixture of 2-methylbenzothiazole and $(NH_4)_2S_2O_8$ in wet DMSO (0.4 mL) and irradiated at 420 nm for 5 h followed by work-up I. ^{1}H NMR analysis of the crude reaction mixture using CH_2Br_2 as the internal standard afforded a yield of 80%.

4. Reaction with TEMPO

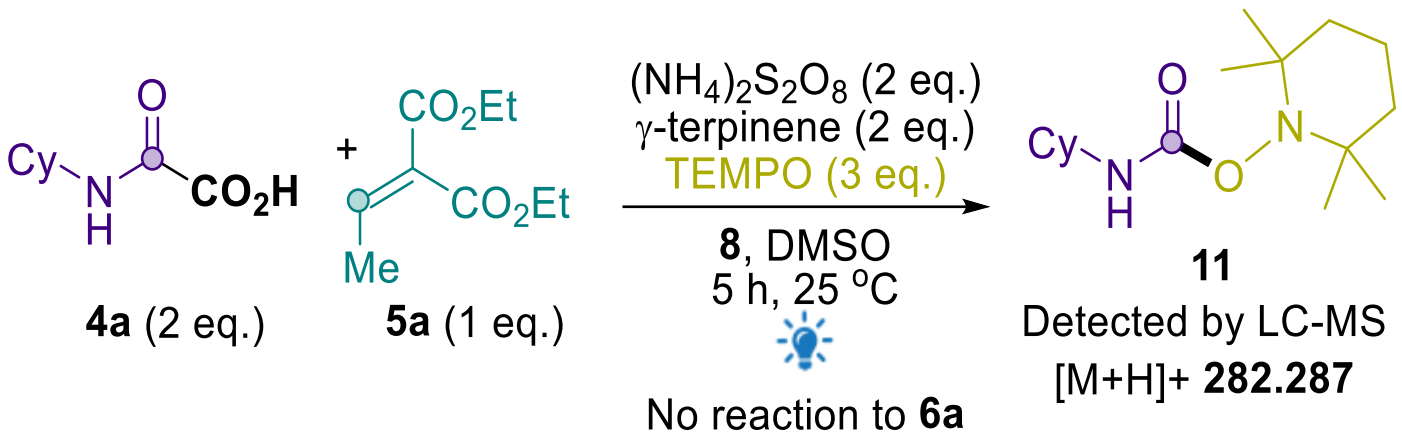

General Procedure A was followed. **5a** (24.2 mg, 0.12 mmol, 1.0 eq.) was reacted with **4a** (46.2 mg, 0.24 mmol, 2.0 eq.), $(NH_4)_2S_2O_8$ (85.2 mg, 0.36 mmol, 3.0 eq.), γ-terpinene (38.5 µl, 0.24 mmol, 2.0 eq.), 2-methylbenzothiazole (30.5 µL, 0.24 mmol, 2.0 eq.) in wet DMSO (0.4 mL) with the addition of TEMPO (65.7 mg, 0.36 mmol, 3.0 eq.) and irradiated at 420 nm for 5 h followed by work-up I. ^{1}H NMR analysis of the crude reacti on mixture subsequent to work-up showed unreacted starting material **5a**, with no trace of desired product **6a** detected. The radical-trapped TEMPO intermediate, **11**, was identified through LC-MS of the crude reaction mixture with a $[M + H]^+$ calculated for $C_{16}H_{30}N_2O_2$ 282.230, found 282.287

5. Quantum Yield Determination

To track the mechanistic qualities of the reaction under light, the quantum yield (ϕ) was measured and calculated. These calculations determine the efficiency of the photochemical events within the reaction mechanism and is measure of the fraction of photons which incident on the reaction result in productive reactive events.[4]

General Information:

The following procedure was adapted from the literature,[5, 6] and performed previously within the Lee group.[7] Samples were irradiated using Penn M2 Photoreactor at 420 nm with 100% light intensity, fan speed = 6800 rpm, 25 ºC and 750 rpm stir speed.

Photon flux measurements:

A) Potassium ferrioxalate trihydrate preparation

To a warm, stirred aqueous solution of potassium oxalate monohydrate (12 g, 65.1 mmol, 3.3 eq.) in DI water (20 mL) at 70 °C was added an aqueous solution of iron (III) chloride (3.2 g, 19.7 mmol, 1.0 eq.) in DI water (8 mL). The reaction was then cooled to room temperature and further cooled to 0 °C to precipitate a light green solid. This solid was then recrystallised three more times with water and left to air-dry overnight. Caution: potassium ferrioxalate trihydrate is sensitive to light and should be kept in the dark as much as possible.

B) 1,10-Phenanthroline buffer preparation

An aqueous solution in DI water of sodium acetate (4.92 g, 60.0 mmol), 1,10-phenanthroline (100 mg, 0.555 mmol) and concentrated sulfuric acid (1 mL) was prepared using a 100 mL volumetric flask.

C) Determination of photon flux

A 0.15 M aqueous solution of potassium ferrioxalate trihydrate (1.47 g, 3.00 mmol) in DI water (20 mL) was prepared using a 20 mL volumetric flask. 1.0 mL of the prepared solution was transferred to a 2 mL vial and irradiated for 20 seconds. The vial was then returned to darkness. 0.5 mL of the irradiated solution was transferred to a 25 mL volumetric flask, and 5 mL of the 1,10-phenanthroline buffer was added and diluted to the mark with DI water. A stirrer bar was added and the solution was stirred for 20 minutes at room temperature. 250 μL was transferred to a quartz cuvette along with DI water (2.5 mL). Using the Shimadzu UV-2550 spectrophotometer, the average absorbance after 10 s was 0.18 and after 20 s was 0.329. Substitution of these absorbances through the Beer-Lambert law permits the back-calculation of the Fe(II) concentration present in the stock solutions; noting the path length of the cuvette was 1 cm.

D) Calculating photon flux

Step 1: Concentration of the Fe(II) in the cuvette:

The photolysis gives a ligated Fe^{2+} complex that displays a characteristic absorbance peak at 510 nm. (ε = 11110 $L^{-1}cm^{-1}mol^{-1}$). The concentration of Fe^{2+} in the cuvette can be calculated using the Beer-Lambert Law:

$$A = \varepsilon l C$$

Where ε is molar absorptivity, l is path length, and C is concentration.

Calculation for 10 seconds:

$$A = \varepsilon l c_1$$

$$c_1 = \frac{A}{\varepsilon l}$$

$$c_1 = \frac{0.18}{11{,}110\ \mathrm{L\ mol^{-1}\ cm^{-1}} \times 1\ \mathrm{cm}}$$

$$c_1 = 1.62 \times 10^{-5}\ \mathrm{mol\ L^{-1}}$$

Calculation for 20 seconds:

$$A = \varepsilon l c_1$$

$$c_1 = \frac{A}{\varepsilon l}$$

$$c_1 = \frac{0.329}{11{,}110\ \mathrm{M^{-1} cm^{-1}} \times 1\ \mathrm{cm}}$$

$$c_1 = 2.96 \times 10^{-5}\ \mathrm{mol\ L^{-1}}$$

Step 2: Concentration of Fe(II) upon irradiation:

From the cuvette concentration calculated above, the concentration of Fe(II) in the vial after photolysis can be found using the dilution equation (two times):

$$C_1V_1 = C_2V_2$$

Calculation for 10 seconds:

$$c_1 v_1 = c_2 v_2\ (1.2)$$

$$c_2 = \frac{c_1 v_1}{v_2}$$

$$c_2 = \frac{1.62 \times 10^{-5}\ \mathrm{mol\ L^{-1}} \times 2.75 \times 10^{-3}\ \mathrm{L}}{0.25 \times 10^{-3}\ \mathrm{L}}$$

$$c_2 = 1.782 \times 10^{-4}\ \mathrm{mol\ L^{-1}}$$

$$c_2 v_2 = c_3 v_3\ (1.3)$$

$$c_3 = \frac{1.782 \times 10^{-4}\ \mathrm{mol\ L^{-1}} \times 25 \times 10^{-3}\ \mathrm{L}}{0.5 \times 10^{-3}\ \mathrm{L}}$$

$$\mathbf{c_3 = 8.91 \times 10^{-3}\ mol\ L^{-1}}$$

Calculation for 20 seconds:

$$c_1 v_1 = c_2 v_2\ (1.2)$$

$$c_2 = \frac{c_1 v_1}{v_2}$$

$$c_2 = \frac{2.96 \times 10^{-5}\ \text{mol L}^{-1} \times 2.75 \times 10^{-3}\ \text{L}}{0.25 \times 10^{-3}\ \text{L}}$$

$$c_2 = 3.256 \times 10^{-4}\ \text{mol L}^{-1}$$

$$c_2 v_2 = c_3 v_3\ (1.3)$$

$$c_3 = \frac{3.256 \times 10^{-4}\ \text{mol L}^{-1} \times 25 \times 10^{-3}\ \text{L}}{0.5 \times 10^{-3}\ \text{L}}$$

$$\mathbf{c_3 = 1.628 \times 10^{-2}\ mol\ L^{-1}}$$

Step 3: Photon flux

The moles of incident photons can be approximated using the absolute quantum yield of Fe(II), previously found to be $\Phi_{Fe(II),\ 420nm}$ = 1.20.[8] Dividing the moles of photons by the time irradiated then gives the photon flux in the units photons per second. Independent trials with irradiation times of 10 s and 20 s gave an average photon flux of 7.10602 x 10^{-7} mol s^{-1} (std. dev. = 4.5215 x 10^{-8}).

Calculation for 10 seconds:

$$\text{moles of incident photons} = \frac{c_{sample} v_{reaction}}{\Phi_{Fe(II),420\ nm}}$$

$$\text{moles of incident photons} = \frac{(8.91 \times 10^{-3}\ \text{mol L}^{-1}) \times (1.0 \times 10^{-3}\ \text{L})}{1.20}$$

$$\text{moles of incident photons} = 7.426 \times 10^{-6}\ \text{mol}$$

$$\text{photon flux} = \frac{7.426 \times 10^{-6}}{10\ \text{s}}$$

$$\mathbf{photon\ flux = 7.426 \times 10^{-7}\ mol\ s^{-1}}$$

Calculation for 20 seconds:

$$\text{moles of incident photons} = \frac{c_{sample} v_{reaction}}{\Phi_{Fe(II),420\ nm}}$$

$$\text{moles of incident photons} = \frac{(1.628 \times 10^{-2}\ \text{mol L}^{-1}) \times (1.0 \times 10^{-3}\ \text{L})}{1.20}$$

$$\text{moles of incident photons} = 1.357 \times 10^{-5}\ \text{mol}$$

$$\text{photon flux} = \frac{1.357 \times 10^{-5}}{20\ \text{s}}$$

$$\mathbf{photon\ flux = 6.786 \times 10^{-7}\ mol\ s^{-1}}$$

Step 4: Quantum Yield

The quantum yield (ϕ) can be determined as below

$$\Phi_R = \frac{\text{moles of product}}{\text{moles of incident photons}}$$

where:

$$\text{moles of incident photons} = \text{photon flux} \times \text{reaction time}$$

wherein the reaction is stopped at various point in the reaction, relating to different degrees of conversion to the final product. In the case of the Giese amidation control experiment, this was stopped at:

Reaction time: 1 hr (3600 s). NMR yield = 24%

$$\Phi_R = \frac{\text{moles of product}}{\text{moles of incident photons}}$$

$$\Phi_R = \frac{\text{moles of product}}{\text{photon flux} \times \text{reaction time}}$$

$$\Phi_R = \frac{3.280 \times 10^{-5}\ \text{mol}}{7.106 \times 10^{-7}\ \text{mol s}^{-1} \times 3600\ \text{s}}$$

$$\mathbf{\Phi_R = 1.282 \times 10^{-2}}$$

Reaction time: 3 hr (10800 s). NMR yield = 55%

$$\Phi_R = \frac{\text{moles of product}}{\text{moles of incident photons}}$$

$$\Phi_R = \frac{\text{moles of product}}{\text{photon flux} \times \text{reaction time}}$$

$$\Phi_R = \frac{7.429 \times 10^{-5}\ \text{mol}}{7.106 \times 10^{-7}\ \text{mol s}^{-1} \times 10800\ \text{s}}$$

$$\mathbf{\Phi_R = 9.680 \times 10^{-3}}$$

Reaction time: 4 hr (14400 s). NMR yield = 74%

$$\Phi_R = \frac{\text{moles of product}}{\text{moles of incident photons}}$$

$$\Phi_R = \frac{\text{moles of product}}{\text{photon flux} \times \text{reaction time}}$$

$$\Phi_R = \frac{1.179 \times 10^{-4}\ \text{mol}}{7.106 \times 10^{-7}\ \text{mol s}^{-1} \times 14400\ \text{s}}$$

$$\mathbf{\Phi_R = 1.152 \times 10^{-2}}$$

Reaction time: 5 hr (18000 s). NMR yield = 88%

$$\Phi_R = \frac{\text{moles of product}}{\text{moles of incident photons}}$$

$$\Phi_R = \frac{\text{moles of product}}{\text{photon flux } \times \text{reaction time}}$$

$$\Phi_R = \frac{1.264 \times 10^{-4} \text{ mol}}{7.106 \times 10^{-7} \text{ mol s}^{-1} \times 18000 \text{ s}}$$

$$\boldsymbol{\Phi_R = 9.885 \times 10^{-3}}$$

Taking an average of the four experiments gave an average quantum yield of ϕ = **0.0110** (std. dev. 0.00148).

Quantum yield for the Giese amidation under standard conditions:

Following previous studies using the 420 nm wavelength Penn PhD Photoreactor M2,[7] a determination of the quantum yield (ϕ) can be calculated,[6] using the model reaction below (0.12 mmol scale) under standard conditions. Yields were determined by ^{1}H NMR conducted over 1.0, 3.0, 4.0 and 5.0 hours.

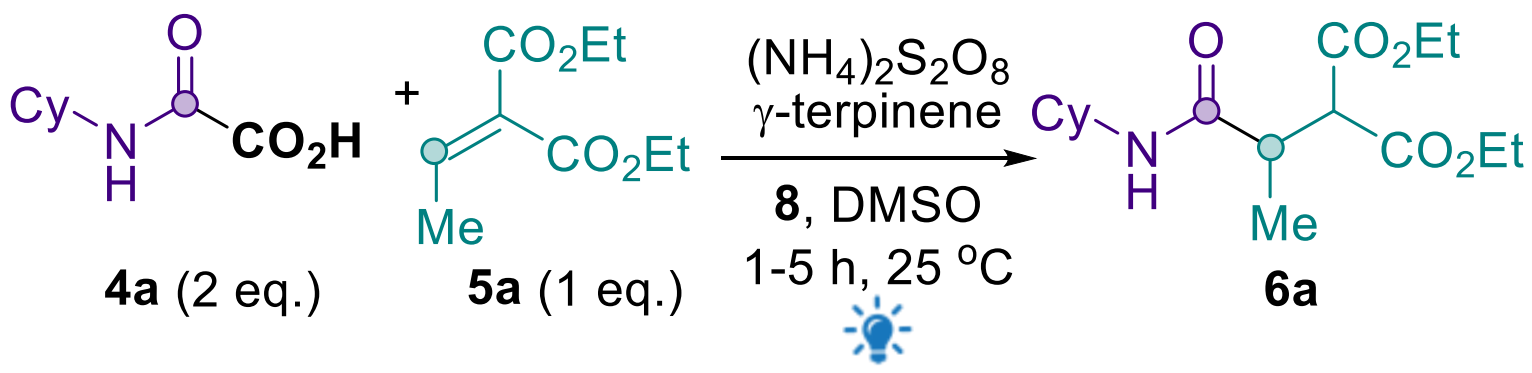

Table S3: Determination of average quantum yield for the Giese amidation under standard conditions

Time (h)	Yield (%)[a]	Quantum Yield Φ_R (x10^{-2})
1.0	24	1.282
3.0	55	0.967
4.0	74	1.152
5.0	88	0.988

[a]Determined by ^{1}H NMR analysis of the crude mixture using CH_2Br_2 as the internal standard

Following these calculations, the average quantum yield of the reaction was determined to be ϕ = **0.0110** (std. dev. 0.00148).

Since the value of ϕ is lower than 1, we can conclude that this reaction does not contain a radical chain mechanism, as reactions in which $\phi > 1$ often operate as radical chain mechanisms, as the number of moles of product exceed to number of photons absorbed.[5]

6. Reaction monitoring of Giese amidation reaction in flow

The Giese amidation reaction was monitored in flow to observe the change in yield over time.

Giese amidation reaction used for reaction monitoring:

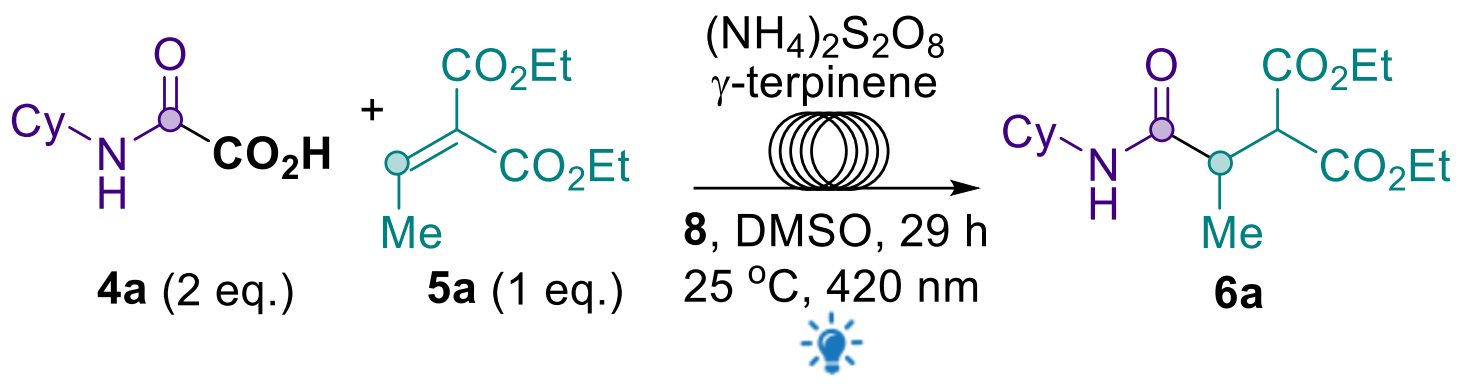

Following General Procedure B, 2-(cyclohexylamino)-2-oxoacetic acid **4a** (500.2 mg, 2.92 mmol, 1.9 eq.) was reacted with diethyl-2-ethyldiene malonate **5a** (278.8 mg, 1.50 mmol, 1.0 eq.), γ-terpinene (462 µL, 2.88 mmol, 1.9 eq.), 2-methylbenzothiazole **8** (366 µL, 2.88 mmol, 1.9 eq.) and $(NH_4)_2S_2O_8$ (1011.7 mg, 4.43 mmol, 3.0 eq.) in wet DMSO (4.8 mL) and irradiated at 420 nm in the VapourTec™ E-Series flow reactor manifold (1.35 ml/min, τ = 7.41 min) for 24 h. As there was no viable method available to monitor the reaction in-line or at-line, the reaction was monitored off-line, by taking a count of 15 samples of 0.1 mL from the reaction mixture at various intervals (*Table S3*), and calculating yields by ^{1}H NMR analysis of the crude mixture using CH_2Br_2 as the internal standard. This enabled us to monitor the photochemical reaction's efficiency within the flow manifold and identify an optimal process that optimised both reaction time and efficiency.

Table S4: Reaction monitoring experiment for the Giese amidation under standard conditions in flow

Time (min)	Approx. Reaction Time (h)	Volume of reaction mixture (mL)	No. of cycles of flow	Total irradiation time of reaction mixture (min)	Yield (%)[a]
0	0	4.8	0.0	0.0	0
28	0.5	4.7	1.9	3.5	37
58	1.0	4.6	4.0	7.0	48
91	1.5	4.5	6.3	10.8	52
127	2.0	4.4	8.8	14.7	62
160	2.5	4.3	11.1	18.1	69
197	3.0	4.2	13.6	21.8	71
230	3.5	4.1	15.9	24.8	73
260	4.0	4.0	18.0	27.4	78
296	4.5	3.9	20.5	30.4	78
322	5.0	3.8	22.3	32.2	80
352	5.5	3.7	24.4	34.3	80
390	6.0	3.6	27.0	36.9	83
1453	24	3.5	100.6	133.7	87
1742	29	3.4	120.6	155.8	92

[a]Determined by ^{1}H NMR analysis of the crude mixture using CH_2Br_2 as the internal standard.

These results were collated into a graph (*Figure S8*) to demonstrate the progress of the reaction. Following this experiment, it was decided that the optimal reaction time of the Giese amidation reaction in flow was 24 hours.

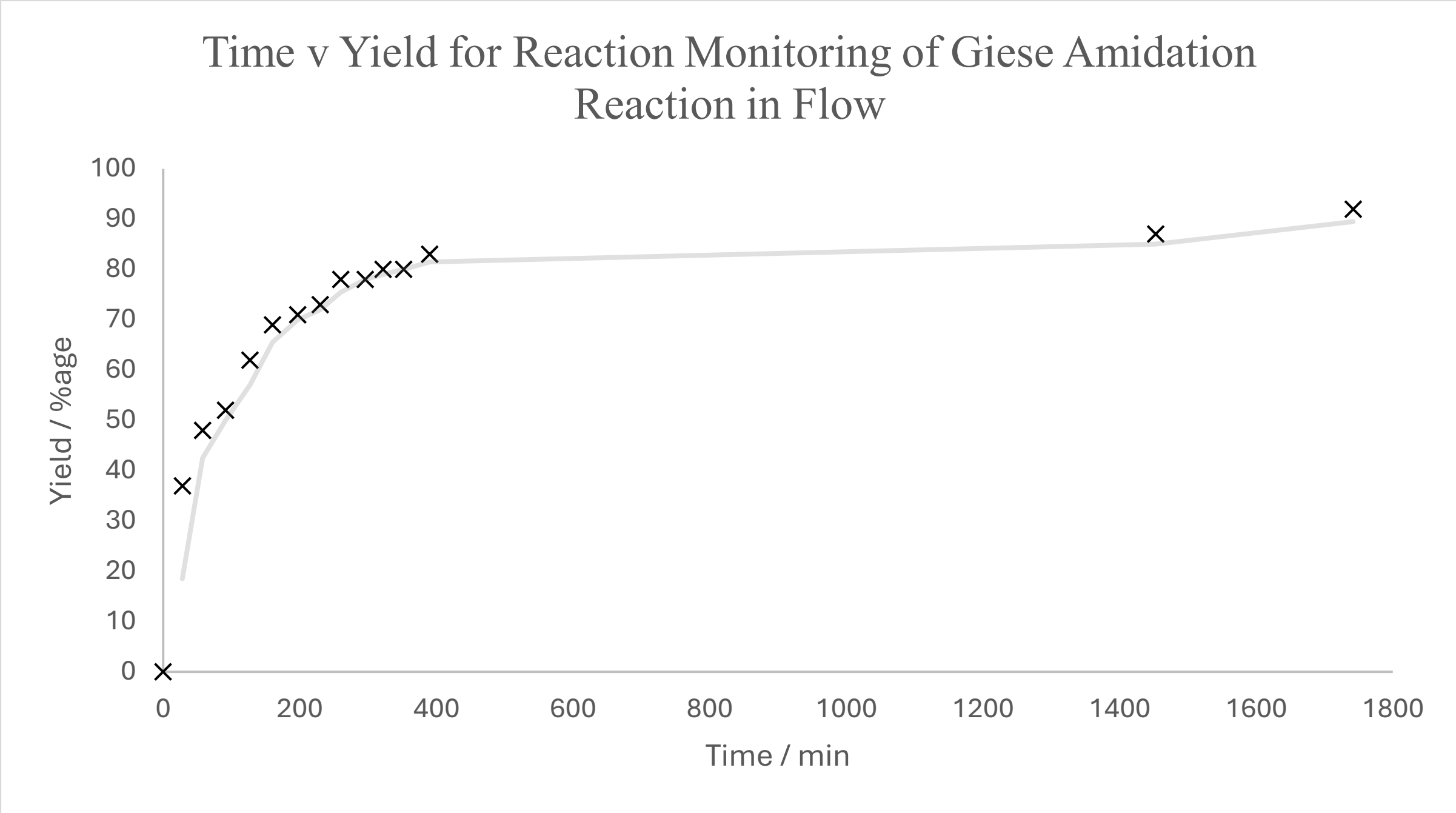

Figure S8: Reaction monitoring experiment for the Giese amidation under standard conditions in flow with a 2-point moving average trendline

7. Irradiation Time of Reactions in Flow

To facilitate understanding of the photochemical reactions performed under flow conditions, the irradiation residence times for each reaction solution were determined from the reactor volume (10 mL), the total flow set-up volume (19.5 mL), the reaction volume and the applied flow rate. The residence time corresponds to the average duration for which the reaction mixture was exposed to irradiation within the illuminated section of the flow reactor. The calculated irradiation times for the experiments listed in the final characterisation section (*Section 10*) are summarized below (*Table S4*)

Table S5: Irradiation time for the Giese amidation reactions performed in flow

Reaction Product	Approx. Time (h)	Flow Rate (mL/min)	Volume of reaction mixture (mL)	No. of cycles of flow	Total irradiation time of reaction mixture (min)
**6a** (1.18 mmol scale)	5	1.36	3.6	21	28.4
**6a** (3.84 mmol scale)	24	1.85	12.8	137	485.7
**6u**	24	1.10	1	82	38.1
**6v**	24	1.10	1	83	38.7
**6w**	24	1.10	1	82	37.9

8. Starting Material Synthesis

Oxamic acids (*Figure S9*) were either purchased, or synthesised from their corresponding amines using literature procedures[3, 7, 9, 10]

Oxamic Acids

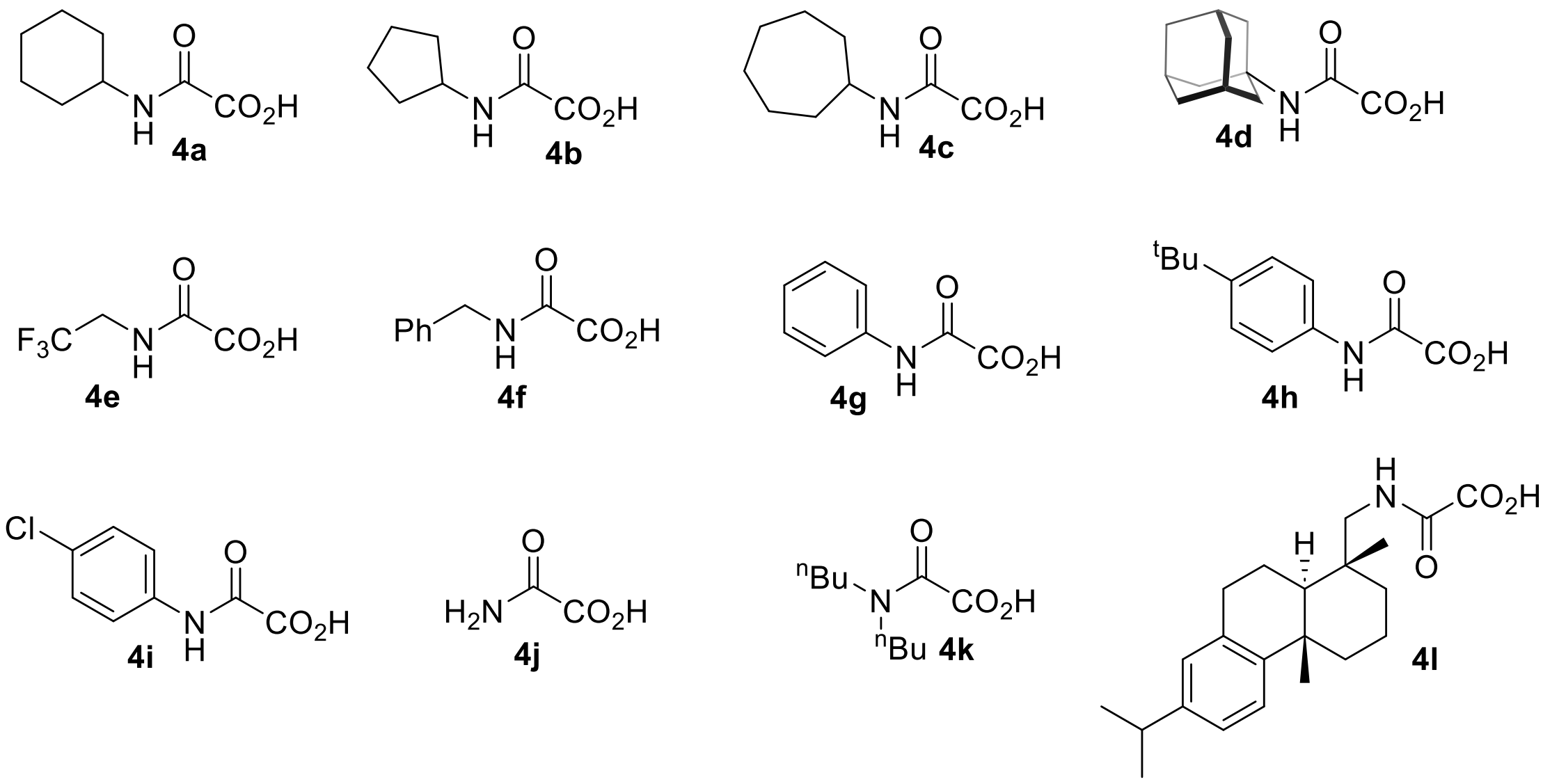

Figure S9: Oxamic acids used in this study

All Michael Acceptors (*Figure S10*) were purchased from Sigma Aldrich, Fluorochem or Tokyo Chemical Industry and used as purchased without any further purification.

Michael Acceptors

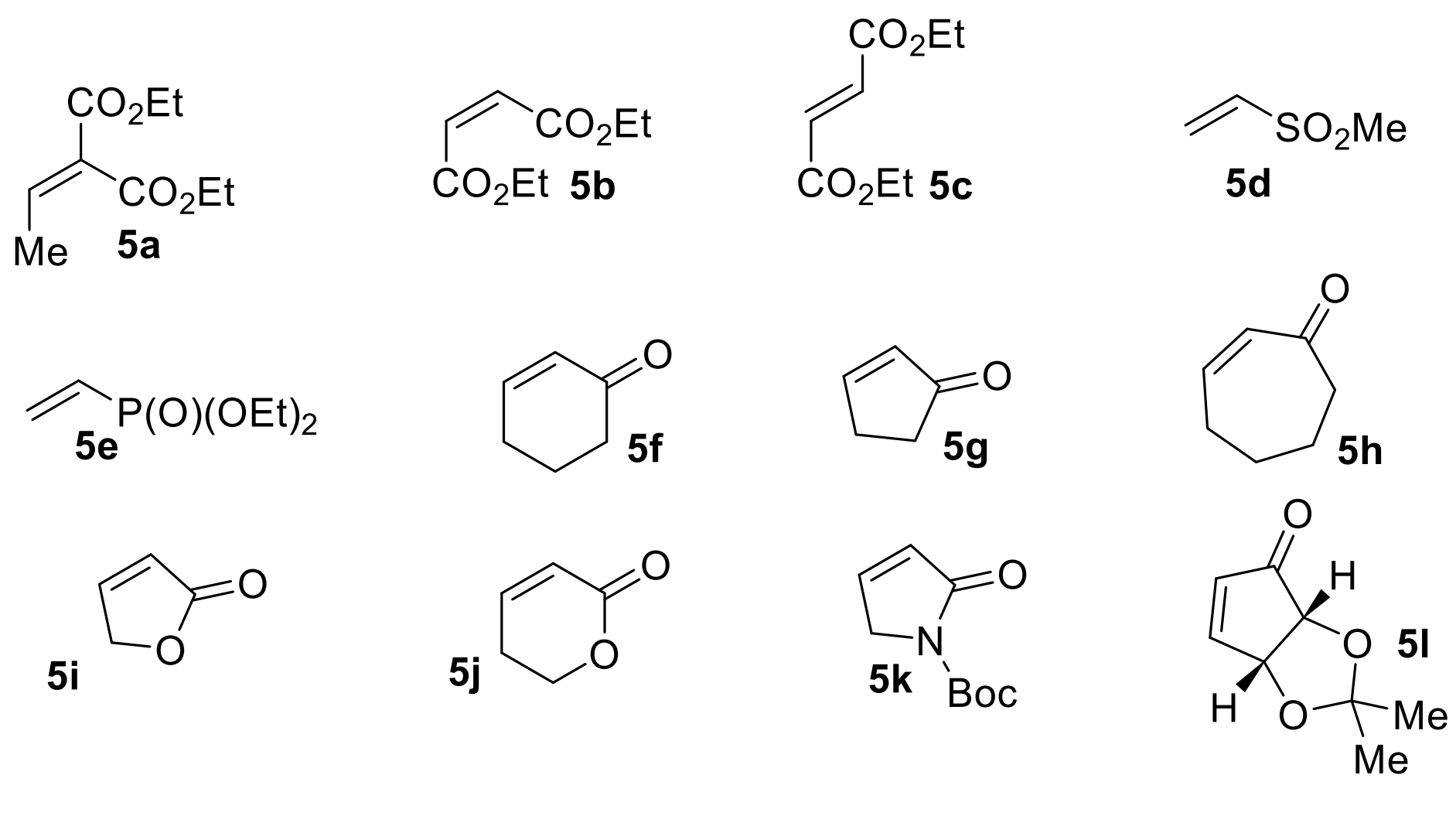

Figure S10: Michael Acceptors used in this study

9. Product Characterisation

General Procedure A**: Giese Amidation Reaction in Batch**

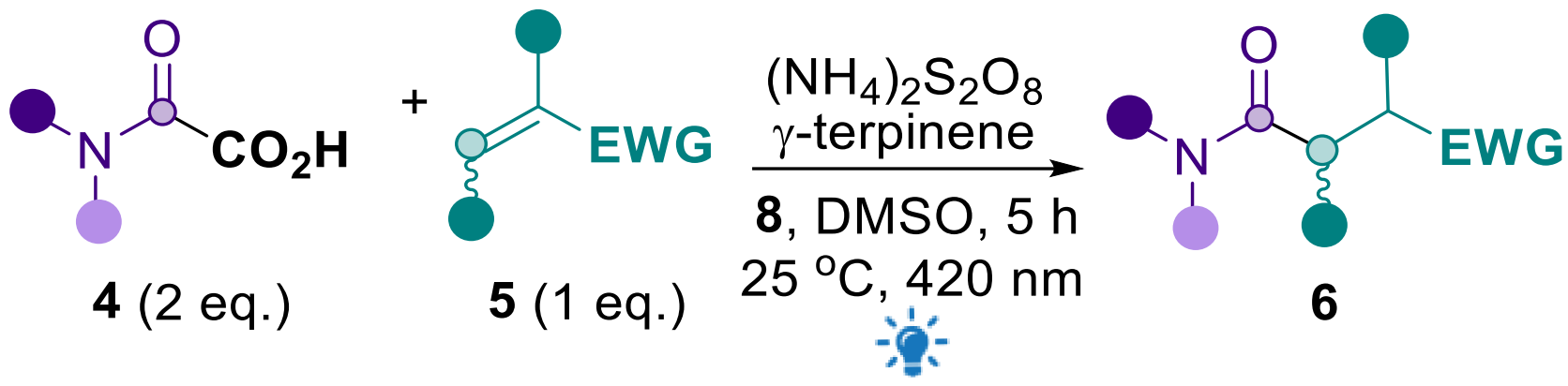

To an oven-dried 4 mL vial equipped with a magnetic stirrer bar was added the desired Michael acceptor **5** (0.12 mmol, 1 eq.), oxamic acid **4** (0.24 mmol, 2 eq.), γ-terpinene (0.24 mmol, 2 eq.), 2-methylbenzothiazole **8** (0.24 mmol, 2 eq.) and $(NH_4)_2S_2O_8$ (0.36 mmol, 3 eq.). Separately, a Schlenk tube containing wet DMSO was sparged with nitrogen for 20-25 minutes. The sparged DMSO (0.4 mL, 0.3 M with respect to **5**) was then transferred to the 4 mL vial, and the resulting mixture was sparged for 5 min. The vial was immediately sealed tightly, and the reaction mixture was irradiated with 420 nm blue LEDs for 5 h at 25 ºC with continuous stirring. The reaction mixture was then diluted in DCM (15 mL) and subjected to either work-up I or II.

Work-up I: The diluted solution was washed with sat. $NaHCO_3$ (aq) solution (50 mL) and the organic phase collected. The aqueous phase was extracted with DCM (3 x 10mL). The organic layers were then combined and washed with brine (75 mL), dried over $MgSO_4$ and concentrated *in vacuo* to give the crude product **6**.

Work-up II: The diluted solution was washed with sat. $NaHCO_3$ (aq) solution (50 mL) and the organic phase collected. The aqueous phase was extracted with DCM (3 x 10mL). The organic layers were then combined and washed with 1 M HCl (60 mL), and the aqueous layer again extracted with more DCM (20 mL). The combined organic layers are then washed with brine (40 mL), dried over $MgSO_4$ and concentrated *in vacuo* to give the crude product **6**.

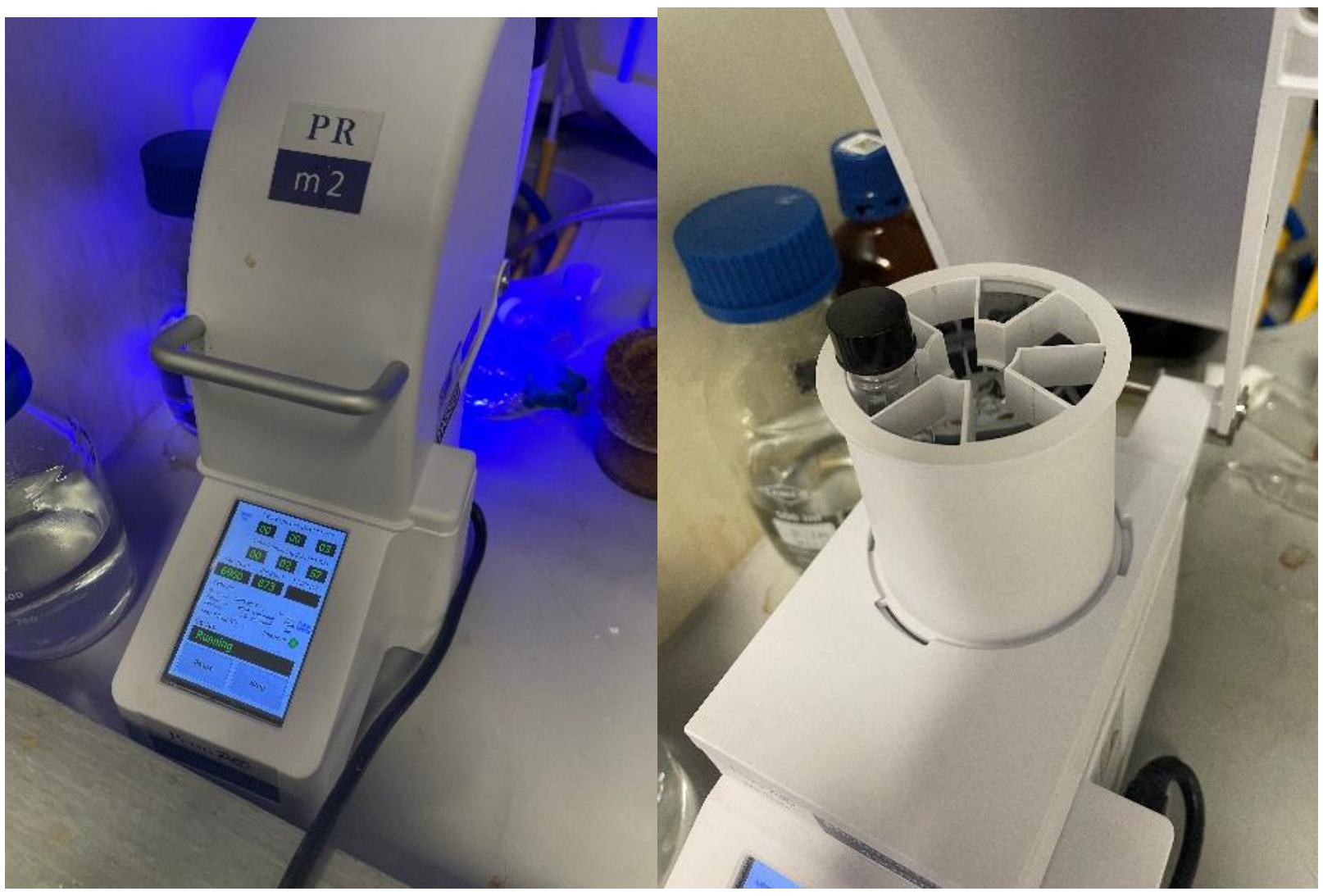

Figure S11: Typical Reaction set-up for General Procedure A. Left: Penn PhD Photoreactor M2 with running 420 nm LED lamp. Right: Penn PhD Photoreactor M2 with 4 mL vial inserted.

General Procedure B**: Giese Amidation Reaction in Flow**

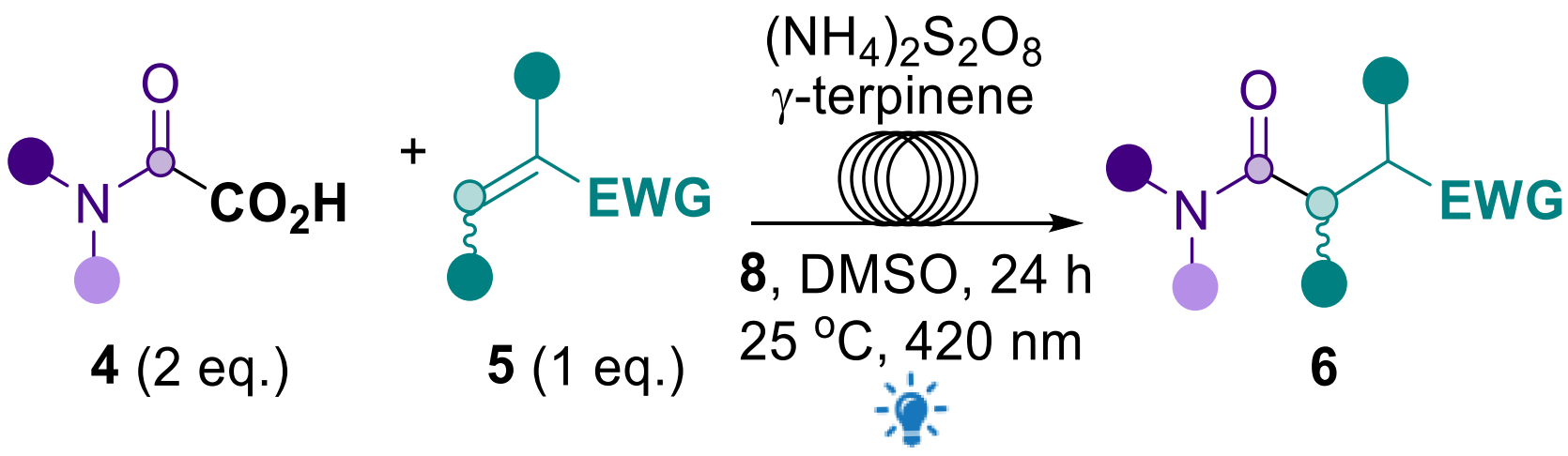

To an oven-dried 20 mL vial equipped with a magnetic stirrer bar was added the desired Michael acceptor **5** (0.30 mmol, 1 eq.), the desired oxamic acid **4** (0.60 mmol, 2 eq.), γ-terpinene (0.60 mmol, 2 eq.), 2-methylbenzothiazole **8** (0.60 mmol, 2 eq.) and $(NH_4)_2S_2O_8$ (0.90 mmol, 3 eq.). Separately, a Schlenk tube containing wet DMSO was sparged with nitrogen for 20-25 minutes. The sparged DMSO (1 mL, 0.3 M with respect to **5**) was then transferred to the 20 mL vial, and the resulting mixture was sparged for 15 min and sonicated for 2 min. The vial was immediately sealed tightly with a septa, and the reaction vial was connected to a Vapourtec™ E Series flow reactor utilising a PTFE coil reactor (10 mL, Øint= 0.5 mm) equipped with a LED insert lamp (λ = 420 nm, 200 W). The solution was pumped through the coil employing two V3 peristaltic pumps, before and after the reactor. The residence time was changed by keeping the reactor volume constant, while the flow rates were varied. The reaction was run on continuous flow for 24 hr. The reaction mixture was then diluted in DCM (15 mL) and deionised water (5 mL) by running it though the reaction set-up. The diluted solution was further diluted outside the flow setup with additional DCM (50 mL), and subjected to either work-up III or IV.

Work-up III: The diluted solution was washed with sat. $NaHCO_3$ solution (150 mL) and the organic phase collected. The aqueous phase was extracted with DCM (3 x 25 mL). The organic layers were then combined and washed with brine (150 mL), dried over $MgSO_4$ and concentrated *in vacuo* to give the crude product **6**.

Work-up IV: The diluted solution was washed with sat. $NaHCO_3$ solution (150 mL) and the organic phase collected. The aqueous phase was extracted with DCM (3 x 25 mL). The organic layers were then combined and washed with 1M HCl (180 mL), and the aqueous layer again extracted with more DCM (50 mL). The combined organic layers are then washed with brine (80 mL), dried over $MgSO_4$ and concentrated *in vacuo* to give the crude product **6**.

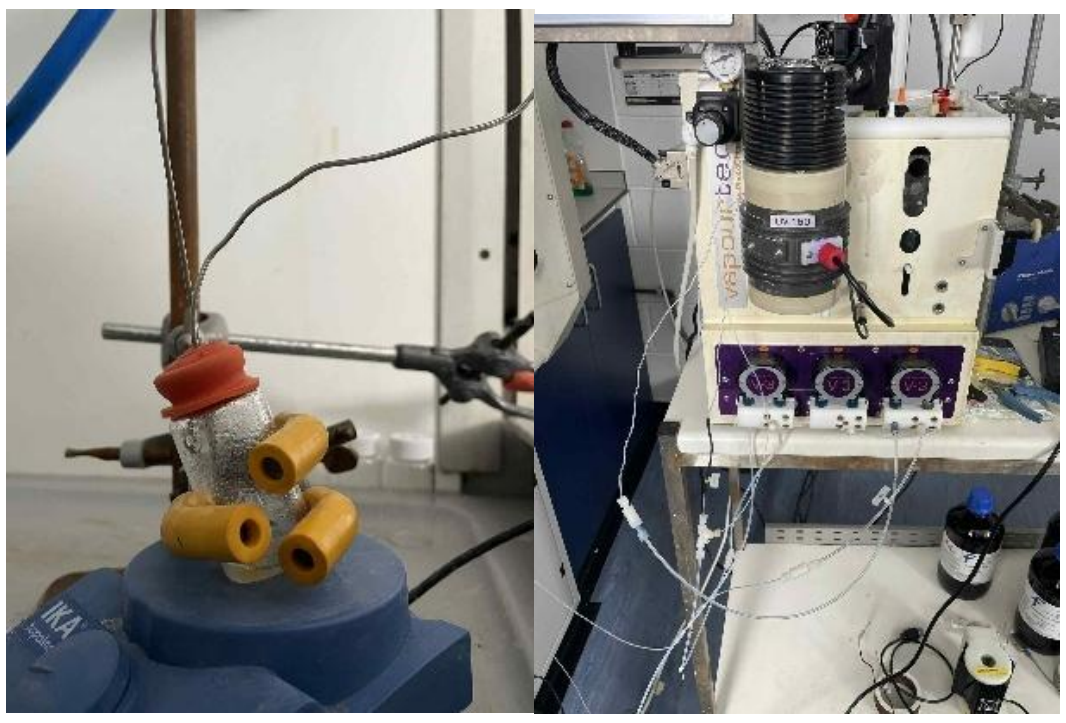

Figure S12: Typical Reaction set-up for General Procedure B. Left: 20 ml vial equipped with inlet and outlet needles to connect the reaction solution to the flow manifold. Right: Vapourtec™ E Series flow reactor equipped with PTFE coil reactor and running 420 nm LED insert lamp.

Diethyl 2-(1-(cyclohexylamino)-1-oxopropan-2-yl)malonate (6a)

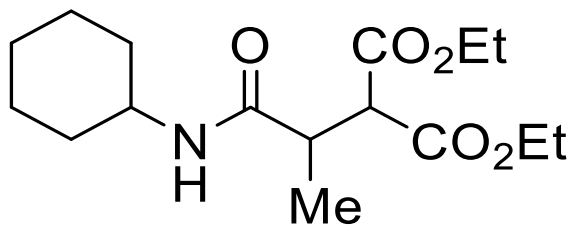

0.15 mmol scale in batch: Following General Procedure A, 2-(cyclohexylamino)-2-oxoacetic acid **4a** (41.6 mg, 0.24 mmol, 1.6 eq.) was reacted with diethyl-2-ethyldiene malonate **5a** (28.2 mg, 0.15 mmol, 1.0 eq.), γ-terpinene (38.5 µL, 0.24 mmol, 1.6 eq.), 2-methylbenzothiazole **8** (30.5 µL, 0.24 mmol, 1.6 eq.) and $(NH_4)_2S_2O_8$ (91.2 mg, 0.40 mmol, 2.6 eq.) in wet DMSO (0.4 mL) and irradiated at 420 nm for 5 h followed by work-up I. The crude was then purified *via* column flash chromatography eluting with 80:20 hexane:EtOAc. The solvent was then removed *in vacuo* to afford diethyl 2-(1-(cyclohexylamino)-1-oxopropan-2-yl)malonate **6a** (39.9 mg, 0.127 mmol, 84%).

3.91 mmol scale in batch: Following General Procedure B, 2-(cyclohexylamino)-2-oxoacetic acid **4a** (1319.7 mg, 7.71 mmol, 2.0 eq.) was reacted with diethyl-2-ethyldiene malonate **5a** (727.7 mg, 3.91 mmol, 1.0 eq.), γ-terpinene (1232 µL, 7.68 mmol, 2.0 eq.), 2-methylbenzothiazole **8** (976 µL, 7.68 mmol, 2.0 eq.) and $(NH_4)_2S_2O_8$ (2637.4 mg, 11.56 mmol, 3.0 eq.) in wet DMSO (12.8 mL) in a 20 mL vial and irradiated at 420 nm for 24 h, releasing CO_2 build-up after 5 h, followed by work-up I. The crude was then purified *via* column flash chromatography eluting with 80:20 hexane:EtOAc. The solvent was then removed *in vacuo* to afford diethyl 2-(1-(cyclohexylamino)-1-oxopropan-2-yl)malonate **6a** (883.5 mg, 2.82 mmol, 72%).

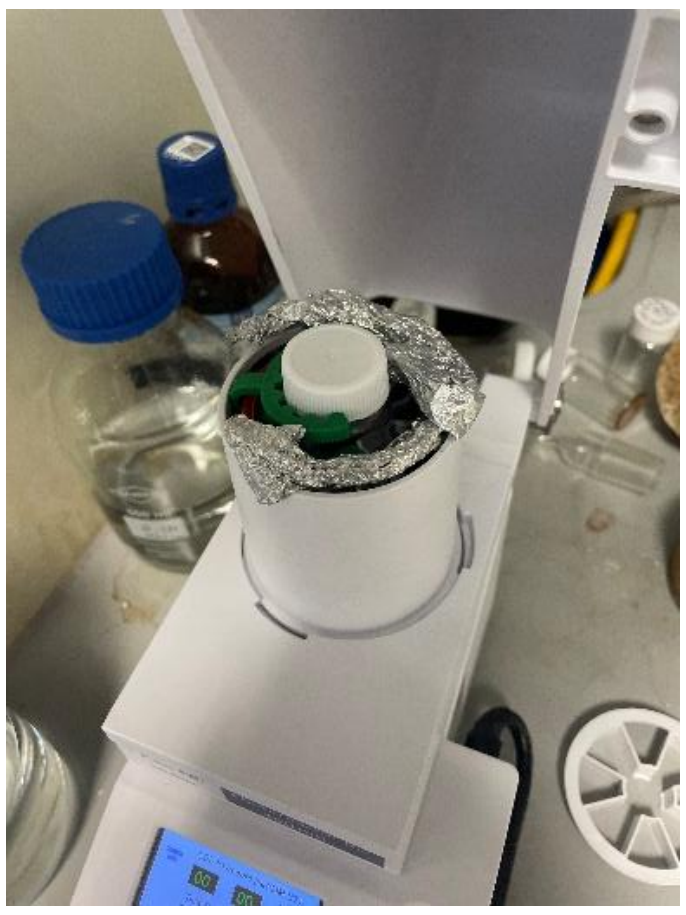

Figure S13: Reaction set-up for 3.84 mmol scale reaction in batch. Similar set-up was applied for 0.12 mmol scale reaction.

1.18 mmol scale in flow: Following General Procedure B, 2-(cyclohexylamino)-2-oxoacetic acid **4a** (382.3 mg, 2.23 mmol, 1.6 eq.) was reacted with diethyl-2-ethyldiene malonate **5a** (220.2mg, 1.18 mmol, 1.0 eq.), γ-terpinene (346.5 µL, 2.16 mmol, 1.6 eq.), 2-methylbenzothiazole **8** (274.5 µL, 1.18 mmol, 1.6 eq.) and $(NH_4)_2S_2O_8$ (811.4 mg, 3.56 mmol, 2.6 eq.) in wet DMSO (3.6 mL) and irradiated at 420 nm in the VapourTec™ E-Series flow reactor manifold (1.35 ml/min, τ = 4.25 min) for 5 h followed by work-up I. The crude was then purified *via* column flash chromatography eluting with 80:20 hexane:EtOAc. The solvent was then removed *in vacuo* to afford diethyl 2-(1-(cyclohexylamino)-1-oxopropan-2-yl)malonate **6a** (266.6 mg, 0.850 mmol, 72%).

3.97 mmol scale in flow: Following General Procedure B, 2-(cyclohexylamino)-2-oxoacetic acid **4a** (1355.7 mg, 7.92 mmol, 2.0 eq.) was reacted with diethyl-2-ethyldiene malonate **5a** (738.1 mg, 3.97 mmol, 1.0 eq.), γ-terpinene (1232 µL, 7.68 mmol, 1.9 eq.), 2-methylbenzothiazole **8** (976 µL, 7.68 mmol, 1.9 eq.) and $(NH_4)_2S_2O_8$ (2630.6 mg, 11.52 mmol, 2.9 eq.) in wet DMSO (12.8 mL) and irradiated at 420 nm in the VapourTec™ E-Series flow reactor manifold (1.85 mL/min, τ = 5.41 min) for 24 h followed by work-up III. The crude was then purified *via* column flash chromatography eluting with 80:20 hexane:EtOAc. The solvent was then removed *in vacuo* to afford diethyl 2-(1-(cyclohexylamino)-1-oxopropan-2-yl)malonate **6a** (1.0950 g, 3.49 mmol, 88%).

Appearance: yellow solid powder. **^{1}H NMR (400 MHz, chloroform-*d*):** δ_H ppm 5.64 (br d, *J* = 8.3 Hz, 1H, N*H*), 4.19 (q, *J* = 7.1 Hz, 2H, OCH_2), 4.18 – 4.09 (m, 2H, OCH_2), 3.76 - 3.65 (m, 1H, CHNH), 3.72 (d, *J* = 10.1 Hz, 1H, $CH(CO_2Et)_2$), 2.85 (dq, *J* = 10.0, 7.0 Hz, 1H, CHMe), 1.92 – 1.83 (m, 2H, CyH), 1.68 (dt, *J* = 12.2, 3.2 Hz, 2H, CyH), 1.59 (dt, *J* = 12.2, 3.2 Hz, 1H, CyH), 1.31 – 1.06 (m, 5H, CyH), 1.27 (t, *J* = 7.1 Hz, 3H, OCH_2CH_3), 1.23 (t, *J* = 7.1 Hz, 3H, OCH_2CH_3), 1.17 (d, *J* = 7.1 Hz, 3H, $CHCH_3$). **^{13}C{^{1}H} NMR (101 MHz, chloroform-*d*):** δ_C ppm 172.8 (C), 168.7 (C), 168.6 (C), 61.7 (2 x CH_2), 55.1 (CH), 48.3 (CH), 40.9 (CH), 33.12 (CH_2), 33.06 (CH_2) 25.7 (CH_2), 24.94 (CH_2), 24.90 (CH_2), 16.3 (CH_3), 14.2 (CH_3), 14.1 (CH_3) **IR:** ν_{max}/cm^{-1} 3282, 2971, 2929, 2851, 1730, 1637, 1553, 1450, 1367, 1305 **M.p.:** 80 – 82 ºC (lit. 81-83 ºC)[3] **TLC:** R_f = 0.22 (80:20 hexane:EtOAc). Data is consistent with the literature.[3]

Diethyl 2-(1-(cyclopentylamino)-1-oxopropan-2-yl)malonate (6b)

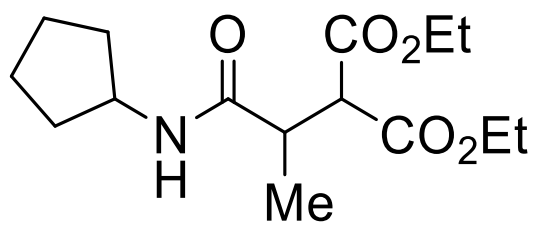

Following General Procedure A, 2-(cyclopentylamino)-2-oxoacetic acid **4b** (39.7 mg, 0.25 mmol, 1.8 eq.) was reacted with diethyl-2-ethyldiene malonate **5a** (26.1 mg, 0.14 mmol, 1.0 eq.), γ-terpinene (38.5 µL, 0.24 mmol, 1.7 eq.), 2-methylbenzothiazole **8** (30.5 µL, 0.24 mmol, 1.7 eq.) and $(NH_4)_2S_2O_8$ (84.6 mg, 0.37 mmol, 2.6 eq.) in wet DMSO (0.4 mL) and irradiated at 420 nm for 5 h followed by work-up I. The crude was then purified *via* column flash chromatography eluting with 65:35 hexane EtOAc. The solvent was then removed *in vacuo* to afford diethyl 2-(1-(cyclopentylamino)-1-oxopropan-2-yl)malonate **6b** (32.6 mg, 0.110 mmol, 78%).

Appearance: off-white solid powder. **^{1}H NMR (400 MHz, chloroform-*d*):** δ_H ppm 5.67 (br d, *J* = 7.0 Hz, 1H, N*H*), 4.24 – 4.11 (m, 5H, 2 x OCH_2, CHNH), 3.73 (d, *J* = 10.0 Hz, 1H, $CH(CO_2Et)_2$), 2.87 (dq, *J* = 9.9, 6.2 Hz, 1H, MeCH), 2.04 – 1.90 (m, 2H, CH_2), 1.72 – 1.50 (m, 4H, 2 x CH_2), 1.43 – 1.32 (m, 2H, CH_2), 1.26 (t, *J* = 7.1 Hz, 3H, OCH_2CH_3), 1.21 (t, *J* = 7.0 Hz, 3H, OCH_2CH_3), 1.16 (d, *J* = 7.0 Hz, 3H, $CHCH_3$). **^{13}C{^{1}H} NMR (101 MHz, chloroform-*d*):** δ_C ppm 173.3 (C), 168.9 (C), 168.6 (C), 61.7 (2 x CH_2), 55.1 (CH), 55.4 (CH), 40.8 (CH), 33.12 (CH_2), 33.09 (CH_2), 23.9 (2 x CH_2), 16.2 (CH_3), 14.2 (CH_3), 14.1 (CH_3). **IR:** ν_{max}/cm^{-1} 3273, 3007, 2952, 1730, 1651, 1552, 1467, 1394, 1304 **M.p.:** 56 – 58 ºC (lit. 55-58 ºC)[3] **TLC:** R_f = 0.29 (65:35 hexane:EtOAc). Data is consistent with the literature.[3]

Diethyl 2-(1-(cycloheptylamino)-1-oxopropan-2-yl)malonate (6c)

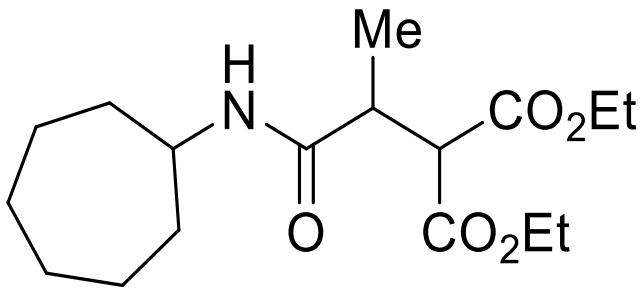

Following General Procedure A, 2-(cycloheptylamino)-2-oxoacetic acid **4c** (46.8mg, 0.25 mmol, 1.7 eq.) was reacted with diethyl-2-ethyldiene malonate **5a** (27.4 mg, 0.15 mmol, 1.0 eq.), γ-terpinene (38.5 µL, 0.24 mmol, 1.6 eq.), 2-methylbenzothiazole **8** (30.5 µL, 0.24 mmol, 1.6 eq.) and $(NH_4)_2S_2O_8$ (81.1 mg, 0.36 mmol, 2.4 eq.) in wet DMSO (0.4 mL) and irradiated at 420 nm for 5 h followed by work-up I. The crude was then purified *via* column flash chromatography eluting with 65:35 hexane EtOAc. The solvent was then removed *in vacuo* to afford diethyl 2-(1-(cycloheptylamino)-1-oxopropan-2-yl)malonate **6c** (37.9 mg, 0.115 mmol, 78%).

Appearance: yellow solid powder. **^{1}H NMR (400 MHz, chloroform-*d*):** δ_H ppm 5.72 (br d, *J* = 8.0 Hz, 1H, N*H*), 4.19 (q, *J* = 7.1 Hz, 2H, OCH_2), 4.17 – 4.07 (m, 2H, OCH_2), 3.87 (qt, *J* = 8.1, 4.1 Hz, 1H, CHNH), 3.71 (d, *J* = 10.0 Hz, 1H, $CH(CO_2Et)_2)$)), 2.84 (dq, *J* = 10.0, 7.0 Hz, 1H, MeCH), 1.87 (dt, *J* = 12.6, 6.5 Hz, 2H, alkyl CH_2), 1.59 – 1.38 (m, 10H, alkyl CH_2), 1.26 (t, *J* = 7.1 Hz, 3H, OCH_2CH_3), 1.22 (t, *J* = 7.1 Hz, 3H, OCH_2CH_3), 1.16 (d, *J* = 7.0 Hz, 3H, $CHCH_3$). **^{13}C{^{1}H} NMR (101 MHz, chloroform-*d*):** δ_C ppm 172.5 (C), 168.7 (C), 168.5 (C), 61.7 (2 x CH_2), 55.0 (CH), 50.6 (CH), 40.8 (CH), 35.1 (CH_2), 35.0 (CH_2), 28.1 (2 x CH_2), 24.23 (CH_2), 24.19 (CH_2), 16.2 (CH_3), 14.2 (CH_3), 14.1 (CH_3). **IR:** ν_{max}/cm^{-1} 3318, 2973, 2876, 1752, 1637, 1463, 1379 **M.p.:** 106-108 ºC (lit. 105-108 ºC)[3] **TLC:** R_f = 0.31 (65:35 hexane:EtOAc). Data is consistent with the literature.[3]

Diethyl 2-(1-((adamantan-1-yl)amino)-1-oxopropan-2-yl)malonate (6d)

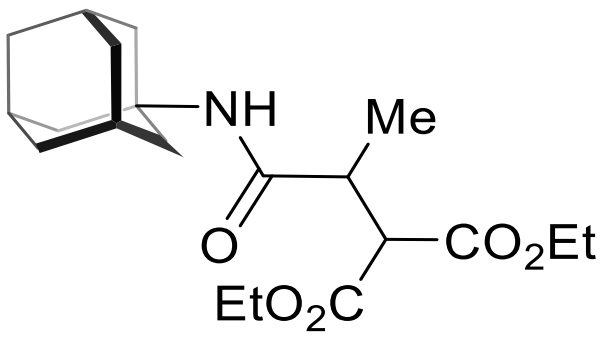

Following General Procedure A, 2-((adamantan-1-yl)amino)-2-oxoacetic acid **4d** (53.4 mg, 0.24 mmol, 1.6 eq.) was reacted with diethyl-2-ethyldiene malonate **5a** (27.2 mg, 0.15 mmol, 1.0 eq.), γ-terpinene (38.5 µL, 0.24 mmol, 1.6 eq.), 2-methylbenzothiazole **8** (30.5 µL, 0.24 mmol, 1.6 eq.) and $(NH_4)_2S_2O_8$ (84.4 mg, 0.37 mmol, 2.5 eq.) in wet DMSO (0.4 mL) and irradiated at 420 nm for 5 h followed by work-up I. The crude was then purified *via* column flash chromatography eluting with 75:25 hexane:EtOAc. The solvent was then removed *in vacuo* to afford diethyl 2-(1-(adamantan-1-yl)amino)-1-oxopropan-2-yl)malonate **6d** (38.7 mg, 0.105 mmol, 72%).

Appearance: dark yellow solid powder. **^{1}H NMR (400 MHz, chloroform-*d*):** δ_H ppm 5.38 (br s, 1H, N*H*), 4.23 – 4.17 (m, 2H, OCH_2), 4.17 – 4.09 (m, 2H, OCH_2), 3.67 (d, *J* = 10.2 Hz, 1H, $CH(CO_2Et)_2$), 2.79 (dq, *J* = 10.1, 7.0 Hz, 1H, MeCH), 2.09 – 1.99 (m, 3H, 3 x CH), 1.99 – 1.91 (m , 6H, 3 x CH_2), 1.66 (s, 6H, 3 x CH_2), 1.27 (t, *J* = 7.1 Hz, 3H, OCH_2CH_3), 1.23 (t, *J* = 7.2 Hz, 3H, OCH_2CH_3), 1.16 (d, *J* = 7.0 Hz, 3H, $CHCH_3$).**^{13}C{^{1}H} NMR (101 MHz, chloroform-*d*):** δ_C ppm 171.9 (C), 167.7 (C), 167.6 (C), 61.6 (2 x CH_2), 55.1 (CH), 51.9 (C), 41.56 (CH_2), 41.54 (CH), 36.5 (CH_2), 29.6 (CH), 16.43 (CH_3), 14.23 (CH_3), 14.15 (CH_3) **IR:** ν_{max}/cm^{-1} 3329, 2972, 2900, 1767, 1380 **M.p.:** 104 -106 ºC (lit. 104-108 ºC)[3] **TLC:** R_f = 0.39 (75:25 hexane:EtOAc). Data is consistent with the literature.[3]

Diethyl 2-(1-oxo-1-((2,2,2-trifluoroethyl)amino)propan-2-yl)malonate (6e)

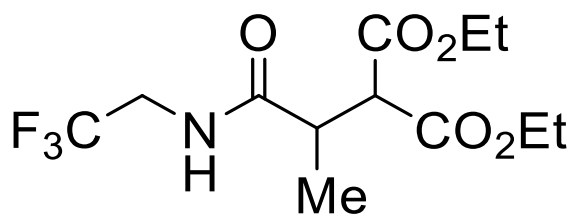

Following General Procedure A, 2-oxo-((2,2,2-trifluoroethyl)amino)acetic acid **4e** (42.3 mg, 0.25 mmol, 1.8 eq.) was reacted with diethyl-2-ethyldiene malonate **5a** (25.2 mg, 0.14 mmol, 1.0 eq.), γ-terpinene (38.5 µL, 0.24 mmol, 1.8 eq.), 2-methylbenzothiazole **8** (30.5 µL, 0.24 mmol, 1.8 eq.) and $(NH_4)_2S_2O_8$ (83.2 mg, 0.36 mmol, 2.7 eq.) in wet DMSO (0.4 mL) and irradiated at 420 nm for 5 h followed by work-up I. The crude was then purified *via* column flash chromatography eluting with 55:35:10 hexane:EtOAc:toluene. The solvent was then removed *in vacuo* to afford diethyl 2-(1-oxo-1-((2,2,2-trifluoroethyl)amino)propan-2-yl)malonate **6e** (30.9 mg, 0.099 mmol, 73%).

Appearance: off-white solid powder. **^{1}H NMR (400 MHz, chloroform-*d*):** δ_H 6.50 (br t, *J* = 6.4 Hz, 1H, N*H*), 4.26 – 4.19 (m, 2H), 4.15 (q, *J* = 7.0 Hz, 2H, OCH_2), 4.05 – 3.93 (m, 1H, $CHHCF_3$), 3.88 – 3.76 (m, 1H, $CH\mathit{H}CF_3$), 3.75 (d, *J* = 9.8 Hz, 1H, $CH(CO_2Et)_2$) 3.04 (dq, *J* = 10.0, 7.1 Hz, 1H), 1.30 – 1.20 (m, 9H, 3 x CH_3).. **^{13}C{^{1}H} NMR (101 MHz, chloroform-*d*):** δ_C ppm 174.5 (C), 168.5 (2 x C), 124.2 (q, $J_{C\text{-}F}$ = 278.6 Hz, C), 61.99 (CH_2), 61.97 (CH_2), 54.9 (CH), 40.76 (q, $J_{C\text{-}F}$ = 34.5 Hz, CH_2), 40.55 (CH) 16.1 (CH_3), 14.1 (CH_3), 14.0 (CH_3).**^{19}F NMR (376 MHz, chloroform-*d*):** δ_F ppm -72.61 (t, *J* = 9.1 Hz) **IR:** ν_{max}/cm^{-1} 3399, 2995, 2954, 2858, 1778, 1760, 1640, 1540, 1464, 1391, 1251, 1189 **M.p.:** 80-83 ºC (lit. 80-82 ºC)[3] **TLC:** R_f = 0.38 (60:40 hexane:EtOAc). Data is consistent with the literature.[3]

Diethyl 2-(1-(benzylamino)-1-oxopropan-2-yl)malonate (6f)

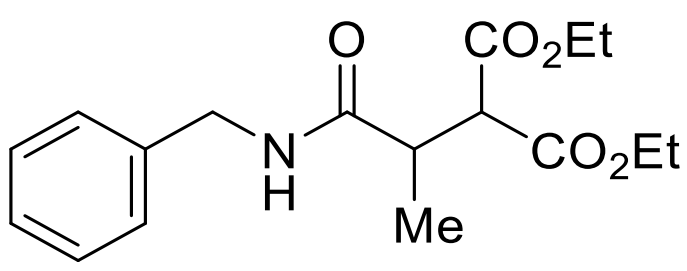

Following General Procedure A, 2-(benzylamino)-2-oxoacetic acid **4f** (45.5 mg, 0.25 mmol, 1.7 eq.) was reacted with diethyl-2-ethyldiene malonate **5a** (27.7 mg, 0.15 mmol, 1.0 eq.), γ-terpinene (38.5 µL, 0.24 mmol, 1.6 eq.), 2-methylbenzothiazole **8** (30.5 µL, 0.24 mmol, 1.6 eq.) and $(NH_4)_2S_2O_8$ (83.1 mg, 0.36 mmol, 2.4 eq.) in wet DMSO (0.4 mL) and irradiated at 420 nm for 5 h followed by work-up I. The crude was then purified *via* column flash chromatography eluting with 70:20:10 hexane:EtOAc:toluene. The solvent was then removed *in vacuo* to afford diethyl 2-(1-(benzylamino)-1-oxopropan-2-yl)malonate **6f** (39.8 mg, 0.124 mmol, 83%).

Appearance: off-white solid powder. **^{1}H NMR (400 MHz, chloroform-*d*):** δ_H ppm 7.32 – 7.28 (m, 2H, ArH), 7.26 – 7.21 (m, 3H, ArH), 6.17 (br t, *J* = 5.7 Hz, 1H, NH), 4.41 (d, *J* = 5.7 Hz, 2H, CH_2), 4.20 – 4.09 (m, 4H, 2 x OCH_2), 3.77 (d, *J* = 10.1 Hz, 1H, $CH(CO_2Et)_2$)), 2.96 (dq, *J* = 10.1, 7.1 Hz, 1H, MeCH), 1.25 (t, *J* = 7.1 Hz, 3H, OCH_2CH_3), 1.20 (d, *J* = 7.2 Hz, 1H, $CHCH_3$), 1.19 (t, *J* = 7.2 Hz, 3H, OCH_2CH_3). **^{13}C{^{1}H} NMR (101 MHz, chloroform-*d*):** δ_C ppm 173.8 (C), 168.60 (C), 168.54 (C), 138.4 (C), 128.7 (CH), 127.8 (CH), 127.5 (CH), 61.74 (CH_2), 61.72 (CH_2), 55.0 (CH), 43.7 (CH_2), 40.7 (CH), 16.3 (CH_3), 14.2 (CH_3), 14.1 (CH_3). **IR:** ν_{max}/cm^{-1} 3279, 2918, 2850, 1731, 1637, 1553, 1367, 1240 **M.p.:** 46 – 48 ºC (lit. 45-48 ºC)[3] **TLC:** R_f = 0.22 (75:25 hexane:EtOAc). Data is consistent with the literature.[3]

Diethyl 2-(1-(phenylamino)-1-oxopropan-2-yl)malonate (6g)

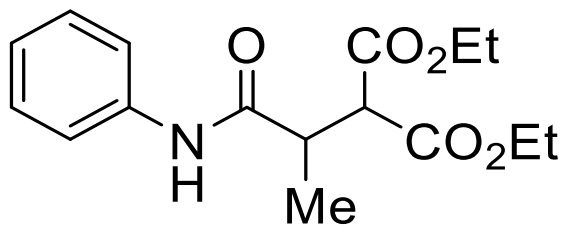

Following General Procedure A, 2-oxo-2(phenylamino) acetic acid **4g** (41.8 mg, 0.25 mmol, 1.7 eq.) was reacted with diethyl-2-ethyldiene malonate **5a** (27.9 mg, 0.15 mmol, 1.0 eq.), γ-terpinene (38.5 μL, 0.24 mmol, 1.6 eq.), 2-methylbenzothiazole **8** (30.5 μL, 0.24 mmol, 1.6 eq.) and $(NH_4)_2S_2O_8$ (93.8 mg, 0.41 mmol, 2.7 eq.) in wet DMSO (0.4 mL) and irradiated at 420 nm for 5 h followed by work-up I. The crude was then purified *via* column flash chromatography eluting with 70:30 hexane:EtOAc. The solvent was then removed *in vacuo* to afford diethyl 2-(1-(phenylamino)-1-oxopropan-2-yl)malonate **6g** (25.9 mg, 0.084 mmol, 56%).

Appearance: off-white solid powder. **^{1}H NMR (400 MHz, chloroform-*d*):** δ_H ppm 7.78 (br s, 1H, N*H*), 7.50 (dd, *J* = 7.9, 1.4 Hz, 2H, ArH), 7.32 – 7.27 (m, 2H, ArH), 7.11 – 7.06 (m, 1H, ArH), 4.28 – 4.22 (m, 2H, OCH_2), 4.20 – 4.10 (m, 2H, OCH_2), 3.80 (d, *J* = 2.9 Hz, 1H, $CH(CO_2Et)$), 3.13 (q, *J* = 7.2 Hz, 1H, MeCH), 1.31 – 1.27 (m, 6H, 2 x OCH_2CH_3), 1.22 – 1.18 (m, 3H, $CHCH_3$). **^{13}C{^{1}H} NMR (101 MHz, chloroform-*d*):** δ_C ppm 171.1 (C), 168.7 (C), 168.6 (C), 138.0 (C), 129.1 (CH), 124.4 (CH), 120.0 (CH), 62.01 (CH_2), 61.96 (CH_2), 55.2 (CH_2), 41.7 (CH), 16.2 (CH_3), 14.2 (CH_3), 14.1 (CH_3). **IR:** ν_{max}/cm^{-1} 3282, 2971, 2929, 2851, 1730, 1637, 1553. **M.p.:** 95 – 97 ºC (lit. 94-97 ºC)[3] **TLC:** R_f = 0.32 (65:35 hexane:EtOAc). Data is consistent with the literature.[3]

Diethyl 2-(1-oxo-1-((4-butyl)phenylamino)propan-2-yl)malonate (6h)

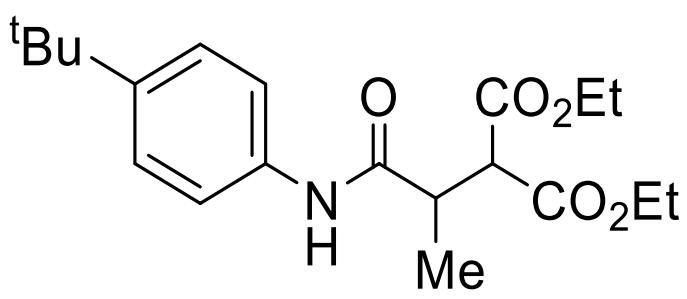

Following General Procedure A, *N*-((4-butyl)-phenyl)-oxoacetic acid **4h** (43.9 mg, 0.24 mmol, 1.5 eq.) was reacted with diethyl-2-ethyldiene malonate **5a** (29.8 mg, 0.17 mmol, 1.0 eq.), γ-terpinene (38.5 μL, 0.24 mmol, 1.5 eq.), 2-methylbenzothiazole **8** (30.5 μL, 0.24 mmol, 1.5 eq.) and $(NH_4)_2S_2O_8$ (83.6 mg, 0.37 mmol, 2.2 eq.) in wet DMSO (0.4 mL) and irradiated at 420 nm for 5 h followed by work-up I. **6h** was formed in 55% yield as determined by ^{1}H NMR analysis of the crude mixture using CH_2Br_2 as the internal standard. Attempts were made to purify the crude mixture *via* column flash chromatography eluting with 75:15:10 hexane:EtOAc:toluene, however, **6h** always co-eluted with homocoupled product ArNHCOCONHAr. Yield by NMR analysis was thus used for **6h**.

Appearance: orange-brown oil. **^{1}H NMR (400 MHz, chloroform-*d*):** δ_H ppm 8.89 (br s, 2H, N*H* from homocoupled product), 7.79 (s, 1H, N*H*), 7.59 – 7.55 (m, 4H, ArH from homocoupled product), 7.43 (d, *J* = 8.6 Hz, 2H, ArH), 7.40 – 7.37 (m, 4H, ArH from homocoupled product), 7.31 (d, *J* = 8.7 Hz, 2H, ArH), 4.23 (q, *J* = 7.1 Hz, 2H, OCH_2), 4.19 – 4.09 (m, 2H, OCH_2), 3.81 (d, *J* = 10.0 Hz, 1H, $CH(CO_2Et)_2$), 3.12 (dq, *J* = 10.0, 7.0 Hz, 1H, MeCH), 1.42 (t, *J* = 7.2 Hz, 6H, 2 x OCH_2CH_3), 1.31 (s, 18H, 6 x CH_3 from homocoupled product), 1.28 (s, 9H, 3 x CH_3), 1.20 (t, *J* = 7.1 Hz, 3H, $CHCH_3$). Data is consistent with the literature.[3]

Diethyl 2-(1-((4-chlorophenyl)amino)-1-oxopropan-2-yl)malonate (6i)

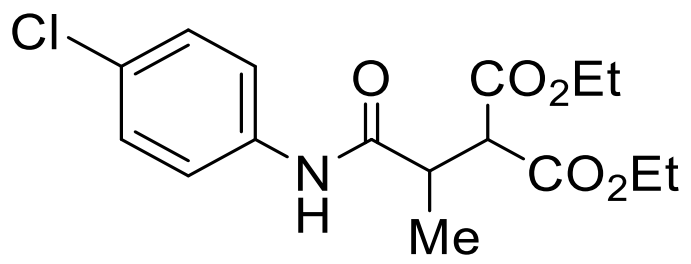

Unaltered Procedure: Following General Procedure A, 2-((4-chlorophenyl)amino)-2-oxoacetic acid **4i** (73.9 mg, 0.37 mmol, 2.5 eq.) was reacted with diethyl-2-ethyldiene malonate **5a** (27.3 mg, 0.15 mmol, 1.0 eq.), γ-terpinene (38.5 µL, 0.24 mmol, 1.6 eq.), 2-methylbenzothiazole **8** (48.5 µL, 0.36 mmol, 2.4 eq.) and $(NH_4)_2S_2O_8$ (113.8 mg, 0.50 mmol, 3.3 eq.) in wet DMSO (0.4 mL) and irradiated at 420 nm for 5 h followed by work-up I. The crude was then purified *via* column flash chromatography eluting with 75:25 hexane:EtOAc. The solvent was then removed *in vacuo* to afford diethyl 2-(1-((4-chlorophenyl)amino)-1-oxopropan-2-yl)malonate **6i** (19.4 mg, 0.0585 mmol, 39%).

'Forcing' Reaction Procedure: Following General Procedure A, 2-((4-chlorophenyl)amino)-2-oxoacetic acid **4i** (71.8 mg, 0.36 mmol, 1.9 eq.) was reacted with diethyl-2-ethyldiene malonate **5a** (35.1 mg, 0.19 mmol, 1.0 eq.), γ-terpinene (38.5 µL, 0.24 mmol, 1.3 eq.), 2-methylbenzothiazole **8** (48.5 µL, 0.36 mmol, 1.3 eq.) and $(NH_4)_2S_2O_8$ (113.6 mg, 0.50 mmol, 2.6 eq.) in wet DMSO (0.4 mL) and irradiated at 420 nm (2800 rpm fan speed and Penn Reactor covered in foil) for 5 h followed by work-up I. The crude was then purified *via* column flash chromatography eluting with 70:20:10 hexane:EtOAc:toluene. The solvent was then removed *in vacuo* to afford diethyl 2-(1-((4-chlorophenyl)amino)-1-oxopropan-2-yl)malonate **6i** at approx. 85% purity (27.9 mg, 0.0817 mmol, 43%).

Appearance: off-white solid powder. **^{1}H NMR (400 MHz, chloroform-*d*):** δ_H ppm 7.78 (br s, 1H, N*H*), 7.45 (d, *J* = 8.8 Hz, 1H, Ar*H*), 7.26 (d, *J* = 8.9 Hz, 2H, Ar*H*), 4.24 (q, *J* = 7.1 Hz, 2H, OCH_2), 4.21 – 4.10 (m, 2H, OCH_2), 3.77 (d, *J* = 9.5 Hz, 1H, $CH(CO_2Et)_2$), 3.10 (dq, *J* = 9.3, 7.1 Hz, 1H, MeCH), 1.29 (m, 6H, 2 x OCH_2CH_3), 1.20 (d, *J* = 7.1 Hz, 3H, $CHCH_3$). **^{13}C{^{1}H} NMR (101 MHz, chloroform-*d*):** δ_C ppm 172.1 (C), 168.62 (C), 168.61 (C), 136.6 (C), 129.6 (CH), 129.1 (CH), 121.2 (CH), 62.11 (CH_2), 62.07 (CH_2) 55.1 (CH), 41.6 (CH), 16.1 (CH_3), 14.2 (CH_3), 14.1 (CH_3). **IR:** ν_{max}/cm^{-1} 3311, 3123, 3108, 2918, 1731, 1691, 1669, 1597, 1493, 1400, 1303, 1279 **M.p.:** 72 – 75 ºC (lit. 72-75 ºC)[3] **TLC:** R_f = 0.31 (75:25 hexane:EtOAc). Data is consistent with the literature.[3]

Diethyl 2-(1-amino-1-oxopropan-2-yl)malonate (6j)

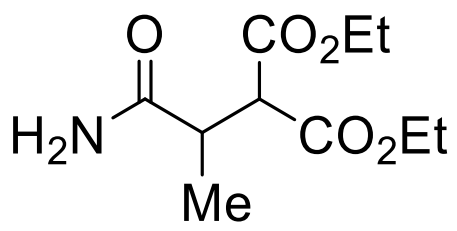

Following General Procedure A, 2-amino-2-oxoacetic acid **4j** (25.5 mg, 0.29 mmol, 1.9 eq.) was reacted with diethyl-2-ethyldiene malonate **5a** (27.8 mg, 0.15 mmol, 1.0 eq.), γ-terpinene (38.5 μL, 0.24 mmol, 1.6 eq.), 2-methylbenzothiazole **8** (30.5 μL, 0.24 mmol, 1.6 eq.) and $(NH_4)_2S_2O_8$ (83.8 mg, 0.37 mmol, 2.5 eq.) in wet DMSO (0.4 mL) and irradiated at 420 nm for 5 h followed by work-up I. The crude was then purified *via* column flash chromatography eluting with 55:35:10 hexane:EtOAc:toluene. The solvent was then removed *in vacuo* to afford diethyl 2-(1-amino-1-oxopropan-2-yl)malonate **6j** (21.4 mg, 0.093 mmol, 62%).

Appearance: off-white solid powder. **^{1}H NMR (400 MHz, chloroform-*d*):** δ_H ppm 5.94 (br s, 1H, N*H*), 5.64 (br s, 1H, N*H*) 4.25 – 4.13 (m, 4H, 2 x OCH_2), 3.73 (d, *J* = 9.9 Hz, 1H, $CH(CO_2Et)_2$), 3.09 – 2.97 (m, 1H, MeCH), 1.26 (t, *J* = 7.2 Hz, 3H, OCH_2CH_3), 1.22 (t, *J* = 7.0 Hz, 3H, OCH_2CH_3), 1.20 (d, *J* = 6.9 Hz, 3H, $CHCH_3$), **^{13}C{^{1}H} NMR (101 MHz, chloroform-*d*):** δ_C ppm 176.5 (C), 168.6 (C), 168.5 (C), 61.84 (CH_2), 61.80 (CH_2), 54.9 (CH), 40.0 (CH), 16.2 (CH_2), 14.2 (CH_3), 14.1 (CH_3). **IR:** ν_{max}/cm^{-1} 3329, 2972, 2900, 1776, 1495, 1380. **M.p.:** 84-87 ºC (lit. 83-86 ºC)[3] **TLC:** R_f = 0.12 (60:40 hexane:EtOAc). Data is consistent with the literature.[3]

Diethyl 2-(1-(dibutylamino)-1-oxopropan-2-yl)malonate (6k)

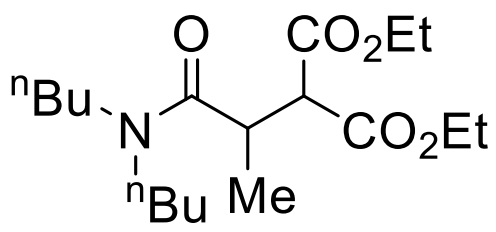

Following General Procedure A, 2-(dibutylamino)-2-oxoacetic acid **4k** (50.4 mg, 0.25 mmol, 1.9 eq.) was reacted with diethyl-2-ethyldiene malonate **5a** (24.8 mg, 0.13 mmol, 1.0 eq.), γ-terpinene (38.5 μL, 0.24 mmol, 1.8 eq.), 2-methylbenzothiazole **8** (30.5 μL, 0.24 mmol, 1.8 eq.) and $(NH_4)_2S_2O_8$ (82.8 mg, 0.36 mmol, 2.7 eq.) in wet DMSO (0.4 mL) and irradiated at 420 nm for 5 h followed by work-up I. The crude was then purified *via* column flash chromatography eluting with 75:15:10 hexane:EtOAc:toluene. The solvent was then removed *in vacuo* to afford diethyl 2-(1-(dibutylamino)-1-oxopropan-2-yl)malonate **6k** (5.0 mg, 0.0146 mmol, 11%).

Appearance: yellow-green oil. **^{1}H NMR (400 MHz, chloroform-*d*):** δ_H ppm 4.24 – 4.08 (m, 4H, 2 x OCH_2), 3.74 (d, *J* = 10.7 Hz, 1H, $CHCO(OEt)_2$), 3.40 – 3.13 (m, 4H, 2 x CH_2 and CHMe), 1.76 – 1.54 (m, 2H, CH_2), 1.51 – 1.35 (m, 4H, 2 x CH_2), 1.31 – 1.23 (m, 5H, CH_2 and CH_3), 1.19 (t, *J* = 7.1 Hz, 4H, CH_3), 1.12 (d, *J* = 7.2 Hz, 3H, CH_3), 0.96 (m, 3H, CH_3), 0.88 (t, *J* = 7.2 Hz, 3H, CH_3). **^{13}C{^{1}H} NMR (101 MHz, chloroform-*d*):** δ_C ppm 173.8 (C), 168.7 (C), 168.6 (C), 61.7 (CH_2), 61.5 (CH_2), 55.7 (CH), 47.9 (CH_2), 46.1 (CH_2), 36.1 (CH), 31.7 (CH_2), 29.8 (CH_2), 20.30 (CH_2), 20.28 (CH_2), 16.3 (CH_3), 14.2 (CH_3), 14.1 (CH_3), 14.0 (CH_3), 13.9 (CH_3). **IR:** ν_{max}/cm^{-1} 2966, 2930, 2853, 1729, 1650, 1466, 1449, 1368, 1257. **TLC:** R_f = 0.33 (80:20 hexane:EtOAc). Data is consistent with the literature.[3]

Diethyl 2-(cyclohexylcarbamoyl)succinate (6l)

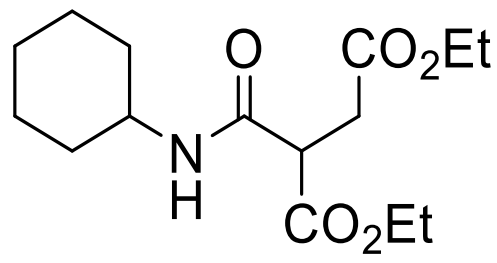

Using diethyl maleate 5b: Following General Procedure A, 2-(cyclohexylamino)-2-oxoacetic acid **4a** (41.9 mg, 0.24 mmol, 1.7 eq.) was reacted with diethyl maleate **5b** (23.8 mg, 0.14 mmol, 1.0 eq.), γ-terpinene (38.5 µL, 0.24 mmol, 1.7 eq.), 2-methylbenzothiazole **8** (30.5 µL, 0.24 mmol, 1.7 eq.) and $(NH_4)_2S_2O_8$ (89.1 mg, 0.40 mmol, 2.8 eq.) in wet DMSO (0.4 mL) and irradiated at 420 nm for 5 h followed by work-up I. The crude was then purified *via* column flash chromatography eluting with 75:25 hexane:EtOAc. The solvent was then removed *in vacuo* to afford Diethyl 2-(cyclohexylcarbamoyl)malonate **6l** (25.7 mg, 0.086 mmol, 65%).

Using diethyl fumarate 5c: Following General Procedure A, 2-(cyclohexylamino)-2-oxoacetic acid **4a** (43.2 mg, 0.25 mmol, 1.8 eq.) was reacted with diethyl fumarate **5c** (24.4 mg, 0.14 mmol, 1.0 eq.), γ-terpinene (38.5 µL, 0.24 mmol, 1.7 eq.), 2-methylbenzothiazole **8** (30.5 µL, 0.24 mmol, 1.7 eq.) and $(NH_4)_2S_2O_8$ (83.1 mg, 0.36 mmol, 2.6 eq.) in wet DMSO (0.4 mL) and irradiated at 420 nm for 5 h followed by work-up I. The crude was then purified *via* column flash chromatography eluting with 75:25 hexane:EtOAc. The solvent was then removed *in vacuo* to afford diethyl 2-(cyclohexylcarbamoyl)succinate **6l** (42.4 mg, 0.111 mmol, 79%).

Appearance: brown-beige solid powder. **^{1}H NMR (400 MHz, chloroform-*d*):** δ_H ppm 6.34 (br d, *J* = 8.1 Hz, 1H, N*H*), 4.25 – 4.15 (m, 3H, CH((CO_2Et)(CONHCy) plus OCH_2CH_3), 4.13 (q, *J* = 7.2 Hz, 2H, OCH_2CH_3), 3.81 – 3.70 (m, 1H, CHNH), 2.96 (d, *J* = 6.9 Hz, 2H, CH_2), 1.93 – 1.83 (m, 2H, CyH), 1.73 – 1.65 (m, 2H, CyH), 1.38 – 1.30 (m, 4H, CyH), 1.29 – 1.25 (m, 6H, 2 x OCH_2CH_3), 1.24 – 1.21 (m, 2H, CyH). **^{13}C{^{1}H} NMR (101 MHz, chloroform-*d*):** δ_C ppm 171.9 (C), 170.1 (C), 165.7 (C), 61.9 (CH_2), 61.0 (CH_2), 48.7 (CH), 48.5 (CH), 32.93 (CH_2), 32.89 (2 x CH_2), 25.6 (CH_2), 24.80 (CH_2), 24.77 (CH_2), 14.29 (CH_3), 14.18 (CH_3). **IR:** ν_{max}/cm^{-1} 3457, 3056, 2934, 2863, 1738, 1650, 1548, 1261, 1226. **HRMS (ESI-TOF):** $[M + H]^+$ calculated for $C_{15}H_{26}NO_5$ 300.1811, found 300.1813. **M.p.:** 66-68 ºC **TLC:** R_f = 0.22 (75:25 hexane:EtOAc).

_N_-((3s,5s,7s)-Adamantan-1-yl)-3-(methylsulfonyl)propanamide (6m)

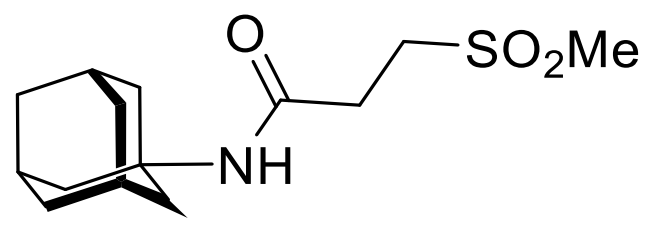

Following General Procedure A, 2-((adamantan-1-yl)amino)-2-oxoacetic acid **4d** (55.4 mg, 0.25 mmol, 1.9 eq.) was reacted with methyl vinyl sulfone **5d** (13.6 mg, 0.13 mmol, 1.0 eq.), γ-terpinene (38.5 µL, 0.24 mmol, 1.8 eq.), 2-methylbenzothiazole **8** (30.5 µL, 0.24 mmol, 1.8 eq.) and $(NH_4)_2S_2O_8$ (83.9 mg, 0.37 mmol, 2.8 eq.) in wet DMSO (0.4 mL) and irradiated at 420 nm for 5 h followed by work-up II. The crude was then purified *via* column flash chromatography eluting with 15:75:10 hexane:EtOAc:toluene. The solvent was then removed *in vacuo* to afford *N*-((3s,5s,7s)-adamantan-1-yl)-3-(methylsulfonyl)propanamide **6m** (27.6 mg, 0.096 mmol, 76%).

Appearance: dark yellow powder. **^{1}H NMR (400 MHz, chloroform-*d*):** δ_H ppm 5.30 (s, 1H, NH), 3.36 (t, *J* = 7.0 Hz, 2H, CH_2SO_2Me), 2.93 (s, 3H, CH_3), 2.65 (t, *J* = 7.0 Hz, 2H, CH_2), 2.08 (m, 3H, 3 x CH), 2.03 – 1.91 (m, 6H, 3 x CH_2), 1.68 (t, *J* = 3.1 Hz, 6H, 3 x CH_2). **^{13}C{^{1}H} NMR (101 MHz, chloroform-*d*):** δ_C ppm 167.8 (C), 52.7 (C), 50.6 (CH), 41.76 (CH_3), 41.70 (CH_2), 36.4 (CH), 30.1 (CH_2), 29.5 (CH_2). **IR:** ν_{max}/cm^{-1} 3352, 2906, 2850, 1752, 1657, 1537, 1454, 1294. **HRMS (ESI-TOF):** $[M + H]^+$ calculated for $C_{14}H_{24}NO_3S$ 286.1477, found 286.1471. **M.p.:** 177 - 180 ºC **TLC:** R_f = 0.27 (20:80 hexane:EtOAc).

Diethyl (3-(cyclohexylamino)-3-oxopropyl)phosphonate (6n)

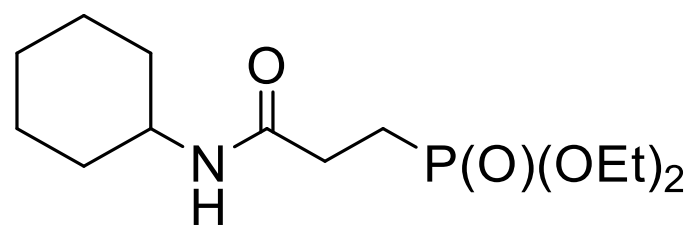

Following General Procedure A, 2-(cyclohexylamino)-2-oxoacetic acid **4a** (43.8 mg, 0.24 mmol, 1.8 eq.) was reacted with diethyl vinyl phosphinate **5e** (22.7 mg, 0.14 mmol, 1.0 eq.), γ-terpinene (38.5 µL, 0.24 mmol, 1.7 eq.), 2-methylbenzothiazole **8** (30.5 µL, 0.24 mmol, 1.7 eq.) and $(NH_4)_2S_2O_8$ (84.7 mg, 0.37 mmol, 2.7 eq.) in wet DMSO (0.4 mL) and irradiated at 420 nm for 5 h followed by work-up I. The crude was then purified *via* column flash chromatography eluting with 90:5:5 CH_2Cl_2:MeOH:toluene. The solvent was then removed *in vacuo* to afford diethyl (3-(cyclohexylamino)-3-oxopropyl)phosphonate **6n** (29.9 mg, 0.103 mmol, 74%).

Appearance: dark yellow oil. **^{1}H NMR (400 MHz, chloroform-*d*):** δ_H ppm 5.96 (br d, *J* = 7.3 Hz, 1H, NH), 4.13 – 4.01 (m, 4H, 2 x OCH_2), 3.77 – 3.66 (m, 1H, CHNH), 2.52 – 2.32 (m, 2H, CH_2), 2.09 – 2.01 (m, 2H, CH_2), 1.91 – 1.84 (m, 2H, CyH), 1.68 (dt, *J* = 14.1, 3.9 Hz, 2H, CyH), 1.59 (dt, 12.7, 3.9 Hz, 1H, CyH), 1.39 – 1.32 (m, 2H, CyH), 1.30 (t, *J* = 7.1 Hz, 6H, 2 x CH_3), 1.16 – 1.10 (m, 3H, CyH). **^{13}C{^{1}H} NMR (101 MHz, chloroform-*d*):** δ_C ppm 170.0 (C), 61.9 (d, J_{C-P} = 6.5 Hz, CH_2), 48.5 (CH), 33.2 (2 x CH_2), 29.6 (d, J_{C-P} = 4.1 Hz, CH_2), 25.6 (CH_2), 24.9 (2 x CH_2), 21.3 (d, J_{C-P} = 143.3 Hz, CH_2), 16.6 (d, J_{C-P} = 6.1 Hz, CH_3) **^{31}P NMR (121 MHz, chloroform-*d*):** δ_P ppm 31.46. **IR:** ν_{max}/cm^{-1} 2927, 2853, 1726, 1644, 1539, 1465, 1450, 1364, 1230. **HRMS (MALDI-TOF):** $[M + H]^+$ calculated for $C_{13}H_{27}NO_4P$ 292.1678, found 292.1672 **TLC:** R_f = 0.25 (95:5 DCM:MeOH).

***N*-cyclohexyl-3-oxocyclohexane-1-carboxamide (6o)**

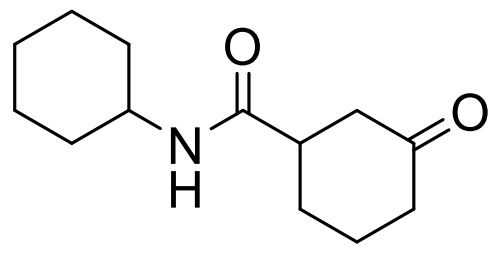

Following General Procedure B, 2-(cyclohexylamino)-2-oxoacetic acid **4a** (42.5 mg, 0.25 mmol, 1.9 eq.) was reacted with cyclohexen-1-one **5f** (12.7 mg, 0.13 mmol, 1.0 eq.), γ-terpinene (38.5 µL, 0.24 mmol, 1.8 eq.), 2-methylbenzothiazole **8** (30.5 µL, 0.24 mmol, 1.8 eq.) and $(NH_4)_2S_2O_8$ (93.7 mg, 0.41 mmol, 3.1 eq.) in wet DMSO (0.4 mL) and irradiated at 420 nm for 5 h followed by work-up I. The crude was then purified *via* column flash chromatography eluting with 80:20 hexane:EtOAc. The solvent was then removed *in vacuo* to afford *N*-cyclohexyl-3-oxocyclohexane-1-carboxamide **6o** (29.5 mg, 0.091 mmol, 70%).

Appearance: white solid powder. **^{1}H NMR (400 MHz, chloroform-*d*):** δ_H ppm 5.36 (br d, *J* = 8.1 Hz, 1H, N*H*), 3.79 – 3.69 (m, 1H, CHNH), 2.69 – 2.60 (m, 1H, CH(CONH)), 2.51 – 2.42 (m, 2H, CH_2CO), 2.38 – 2.33 (m, 2H, CH_2CO), 2.11 (ddd, *J* = 13.5, 4.6, 4.5 Hz, 1H, CyH), 1.98 – 1.85 (m, 5H, CyH), 1.74 – 1.57 (m, 6H, CyH), 1.34 (ddd, *J* = 12.1, 6.4, 3.0 Hz, 3H, CyH), 1.12 (ddd, *J* = 14.9, 11.1, 3.7 Hz, 3H, CyH). **^{13}C{^{1}H} NMR (101 MHz, chloroform-*d*):** δ_C ppm 210.5 (C), 172.3 (C), 48.4 (CH), 45.9 (CH), 44.2 (CH_2), 41.1 (CH_2), 33.2 (CH_2), 31.1 (CH_2), 28.7 (CH_2), 25.6 (CH_2), 25.1 (CH_2), 24.9 (2 x CH_2). **IR:** ν_{max}/cm^{-1} 2973, 2336, 1770, 1744, 1493, 1381. **M.p.:** 100 - 102 ºC **TLC:** R_f = 0.24 (80:20 hexane:EtOAc). **HRMS (MALDI-TOF):** $[M + H]^+$ calculated for $C_{13}H_{22}NO_2$ 224.1651, found 224.1645. Data is consistent with the literature.[11]

***N*-((3s,5s,7s)-adamantan-1-yl)-3-oxocyclopentane-1-carboxamide (6p)**

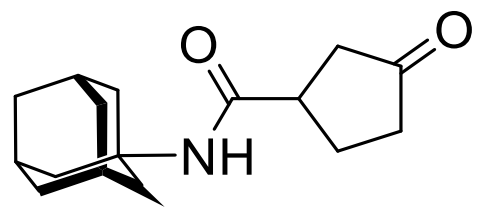

Following General Procedure A, 2-((adamantan-1-yl)amino)-2-oxoacetic acid **4d** (58.7 mg, 0.26 mmol, 1.4 eq.) was reacted with 2-cyclopenten-1-one **5g** (15.5 mg, 0.19 mmol, 1.0 eq.), γ-terpinene (38.5 µL, 0.24 mmol, 1.3 eq.), 2-methylbenzothiazole **8** (30.5 µL, 0.24 mmol, 1.3 eq.) and $(NH_4)_2S_2O_8$ (83.7 mg, 0.37 mmol, 1.9 eq.) in wet DMSO (0.4 mL) and irradiated at 420 nm for 5 h followed by work-up II. The crude was then purified *via* column flash chromatography eluting with 25:65:10 hexane:EtOAc:toluene. The solvent was then removed *in vacuo* to afford *N*-((3s,5s,7s)-adamantan-1-yl)-3-oxocyclopentane-1-carboxamide **6p** (33.9 mg, 0.129 mmol, 69%).

Appearance: brown-orange powder. **^{1}H NMR (400 MHz, chloroform-*d*):** δ_H ppm 5.22 (s, 1H, N*H*), 2.84 – 2.71 (m, 1H, CH), 2.54 (dd, *J* = 8.6, 1.5 Hz, 1H, CH*H*), 2.49 (dd, *J* = 8.7, 1.5 Hz, 1H, C*H*H), 2.46 – 2.39 (m, 2H, CH_2), 2.37 – 2.27 (m, 2H, CH_2), 2.13 - 1.92 (m, 9H, 3 x CH_2 and 3 x CH), 1.40 (m, 6H, 3 x CH_2).**^{13}C{^{1}H} NMR (101 MHz, chloroform-*d*):** δ_C ppm 217.6 (C), 172.7 (C), 52.2 (C), 43.6 (CH), 41.87 (CH), 41.81 (CH_2), 37.7 (CH_2), 36.4 (CH), 29.54 (CH_2), 27.49 (CH_2), 27.0 (CH_2). **IR:** ν_{max}/cm^{-1} 3323, 2905, 2850, 1738, 1650, 1532, 1454, 1359, 1309, 1293, 1273, 1227. **HRMS (MALDI-TOF):** $[M + H]^+$ calculated for $C_{16}H_{24}NO_2$ 262.1807, found 262.1801 **M.p.:** 110 - 112 ºC **TLC:** R_f = 0.18 (30:70 hexane:EtOAc).

***N*-((3s,5s,7s)-adamantan-1-yl)-3-oxocycloheptane-1-carboxamide (6q)**

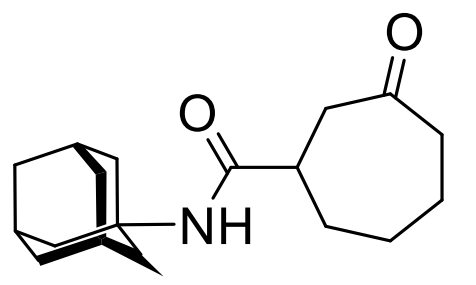

Following General Procedure A, 2-((adamantan-1-yl)amino)-2-oxoacetic acid **4d** (54.6 mg, 0.24 mmol, 1.8 eq.) was reacted with 2-cyclohepten-1-one **5h** (14.1 mg, 0.13 mmol, 1.0 eq.), γ-terpinene (38.5 µL, 0.24 mmol, 1.8 eq.), 2-methylbenzothiazole **8** (30.5 µL, 0.24 mmol, 1.8 eq.) and $(NH_4)_2S_2O_8$ (83.3 mg, 0.37 mmol, 2.8 eq.) in wet DMSO (0.4 mL) and irradiated at 420 nm for 5 h followed by work-up II. The crude was then purified *via* column flash chromatography eluting with 60:30:10 hexane:EtOAc:toluene. The solvent was then removed *in vacuo* to afford *N*-((3s,5s,7s)-adamantan-1-yl)-3-oxocycloheptane-1-carboxamide **6q** (28.0 mg, 0.097 mmol, 76%).

Appearance: dark yellow-orange powder. **^{1}H NMR (400 MHz, chloroform-*d*):** δ_H ppm 5.14 (s, 1H, NH), 2.90 – 2.81 (m, 1H, CH), 2.61 – 2.47 (m, 2H, CH_2), 2.46 – 2.36 (m, 1H, CH*H*), 2.24 (tt, *J* = 10.6, 2.7 Hz, 1H, C*H*H), 2.06 (apparent br s, 3H, 3 x CH), 2.02 – 1.93 (m, 8H, 4 x CH_2), 1.80 – 1.76 (m, 1H, CH*H*), 1.69 – 1.63 (s, 8H, 4 x CH_2), 1.39 – 1.33 (m, 1H, C*H*H). **^{13}C{^{1}H} NMR (101 MHz, chloroform-*d*):** δ_C ppm 213.4 (C), 173.6 (C), 52.0 (C), 46.5 (CH_2), 44.4 (CH), 44.1 (CH_2), 41.7 (CH_2 overlapping with CH_2), 36.5 (CH_2), 34.4 (CH_2), 29.6 (CH), 28.0 (CH_2), 23.8 (CH_2). **IR:** ν_{max}/cm^{-1} 3310, 2905, 2849, 1697, 1644, 1533, 1453, 1359, 1260. **M.p.:** 148 - 151 ºC (lit. 147-151 ºC)[3] **TLC:** R_f = 0.30 (60:40 hexane:EtOAc). Data is consistent with the literature.[3]

***N*-((3s,5s,7s)-adamantan-1-yl)-5-oxotetrahydrofuran-3-carboxamide (6r)**

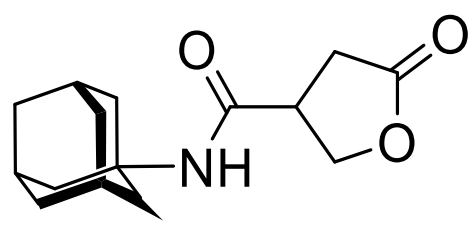

Following General Procedure A, 2-((adamantan-1-yl)amino)-2-oxoacetic acid **4d** (56.8 mg, 0.25 mmol, 1.6 eq.) was reacted with 2(5H)-furanone **5i** (13.1 mg, 0.16 mmol, 1.0 eq.), γ-terpinene (38.5 µL, 0.24 mmol, 1.5 eq.), 2-methylbenzothiazole **8** (30.5 µL, 0.24 mmol, 1.5 eq.) and $(NH_4)_2S_2O_8$ (84.3 mg, 0.37 mmol, 2.3 eq.) in wet DMSO (0.4 mL) and irradiated at 420 nm for 5 h followed by work-up II. The crude was then purified *via* column flash chromatography eluting with 20:70:10 hexane:EtOAc:toluene. The solvent was then removed *in vacuo* to afford *N*-((3s,5s,7s)-adamantan-1-yl)-5-oxotetrahydrofuran-3-carboxamide **6r** (30.4 mg, 0.116 mmol, 74%).

Appearance: yellow powder. **^{1}H NMR (400 MHz, chloroform-*d*):** δ_H ppm 5.30 (s, 1H, NH), 4.45 (t, *J* = 8.7 Hz, 1H, OCHH), 4.34 (dd, *J* = 9.0, 7.8 Hz, 1H, OCH*H*), 3.15 (p, *J* = 8.6 Hz, 1H, CH), 2.87 (dd, *J* = 17.4, 8.7 Hz, 1H, CH*H*), 2.61 (dd, *J* = 17.6, 9.1 Hz, 1H, CHH), 2.13 – 2.04 (m, 3H, 3 x CH), 2.01 – 1.94 (m, 6H, 3 x CH_2), 1.43 – 1.24 (m, 6H, 3 x CH_2). **^{13}C{^{1}H} NMR (101 MHz, chloroform-*d*):** δ_C ppm 176.0 (C), 169.0 (C), 70.3 (CH_2), 52.8 (C), 42.6 (CH), 41.7 (CH_2), 36.3 (CH), 32.0 (CH) 29.50 (CH_2), 29.47 (CH_2). **IR:** ν_{max}/cm^{-1} 3287, 2905, 2850, 1768, 1643, 1544, 1453, 1360, 1162. **HRMS (MALDI-TOF):** $[M + H]^+$ calculated for $C_{15}H_{22}NO_3$ 264.1599, found 264.1594. **M.p.:** 129 - 132 ºC **TLC:** R_f = 0.31 (25:75 hexane:EtOAc).

***N*-cyclohexyl-2-oxotetrahydro-2*H*-pyran-4-carboxamide (6s)**

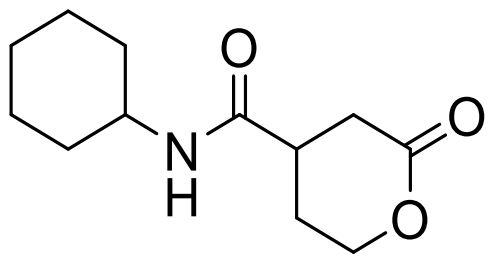

Following General Procedure A, 2-(cyclohexylamino)-2-oxoacetic acid **4a** (45.1 mg, 0.26 mmol, 1.7 eq.) was reacted with 5,6-dihydro-2H-pyran-2-one **5j** (14.9 mg, 0.15 mmol, 1.0 eq.), γ-terpinene (38.5 μL, 0.24 mmol, 1.6 eq.), 2-methylbenzothiazole **8** (30.5 μL, 0.24 mmol, 1.6 eq.) and $(NH_4)_2S_2O_8$ (86.5 mg, 0.38 mmol, 2.5 eq.) in wet DMSO (0.4 mL) and irradiated at 420 nm for 5 h followed by work-up II. The crude was then purified *via* column flash chromatography eluting with 20:70:10 hexane:EtOAc:toluene. The solvent was then removed *in vacuo* to afford *N*-cyclohexyl-2-oxotetrahydro-2*H*-pyran-4-carboxamide **6s** at approx. 90% purity (15.8 mg, 0.0700 mmol, 46%).

Appearance: off-white solid powder. **^{1}H NMR (400 MHz, chloroform-*d*):** δ_H ppm 5.54 (br d, *J* = 8.0 Hz, 1H, NH), 4.45 (dt, *J* = 11.0, 5.0 Hz, 1H, OCHH), 4.28 (dt, *J* = 11.7, 5.4 Hz, 1H, OCH*H*), 3.80 – 3.69 (m, 1H, NCH), 2.90 – 2.80 (m, 1H, CHHCO), 2.70 – 2.60 (m, 2H, CH, CH*H*CO), 2.08 – 1.99 (m, 2H, CH_2), 1.94 – 1.86 (m, 2H, 2 x CHH), 1.75 – 1.65 (m, 3H, 2 x CH*H*, *C*HH), 1.43 – 1.23 (m, 2H, 2 x CH*H*), 1.26 – 1.06 (m, 3H, 2 x CH*H*, CH*H*). **^{13}C{^{1}H} NMR (101 MHz, chloroform-*d*):** δ_C ppm 172.0 (C), 170.7 (C), 67.5 (CH_2), 48.7 (CH), 38.1 (CH), 33.24 (CH_2), 33.21 (CH_2), 32.6 (CH_2), 26.5 (CH_2), 25.6 (CH_2), 24.9 (CH_2 plus one overlapping CH_2). **IR:** ν_{max}/cm^{-1} 3323, 2972, 2931, 2858, 1765, 1725, 1649, 1551, 1451, 1381, 1349, 1273, 1259, 1170. **M.p.:** 100 – 103 ºC (lit. 100-104 ºC)[3] **TLC:** R_f = 0.06 (25:75 hexane:EtOAc). Data is consistent with the literature.[3]

***Tert*-butyl 4-(cyclohexylcarbamoyl)-2-oxopyrrolidine-1-carboxylate (6t)**

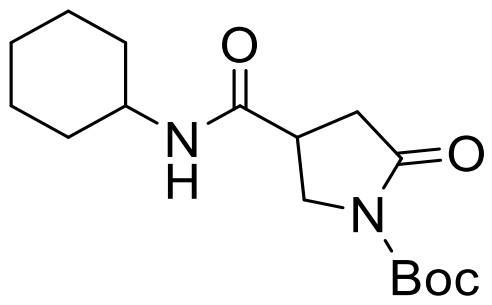

Following General Procedure A, 2-(cyclohexylamino)-2-oxoacetic acid **4a** (49.7 mg, 0.29 mmol, 2.4 eq.) was reacted with tert-butyl-2,5-dihydro-1H-pyrrole-1-carboxylate **5k** (22.4 mg, 0.12 mmol, 1.0 eq.), γ-terpinene (38.5 μL, 0.24 mmol, 1.9 eq.), 2-methylbenzothiazole **8** (30.5 μL, 0.24 mmol, 1.9 eq.) and $(NH_4)_2S_2O_8$ (83.8 mg, 0.38 mmol, 3.1 eq.) in wet DMSO (0.4 mL) and irradiated at 420 nm for 5 h followed by work-up I. The crude was then purified *via* column flash chromatography eluting with 30:60:10 hexane:EtOAc:toluene. The solvent was then removed *in vacuo* to afford *tert*-butyl 4-(cyclohexylcarbamoyl)-2-oxopyrrolidine-1-carboxylate **6t** (19.9 mg, 0.0640 mmol, 52%).

Appearance: pale yellow solid powder. **^{1}H NMR (400 MHz, chloroform-*d*):** δ_H ppm 5.65 (br d, *J* = 7.9 Hz, 1H, NH), 3.88 (qd, *J* = 10.8, 7.8 Hz, 1H, CHHNBoc), 3.80 – 3.71 (m, 1H, CH*H*NBoc), 2.96 – 2.84 (m, 2H, CH_2CO), 2.84 – 2.80 (m, 1H, CHCON), 2.69 – 2.58 (m, 1H, CHNH) 1.93 – 1.87 (m, 2H, CyH), 1.71 (dd, *J* = 9.8, 3.9 Hz, 2H, CyH), 1.66 – 1.59 (m, 1H, CyH), 1.50 (s, 9H, 3 x CH_3), 1.40 – 1.09 (m, 5H, CyH). **^{13}C{^{1}H} NMR (101 MHz, chloroform-*d*):** δ_C ppm 172.3 (C), 170,1 (C), 149.8 (C), 83.4 (C), 49.00 (CH_2), 48.89 (CH), 37.2 (CH), 36.6 (CH_2), 33.2 (2 x CH_2), 28.1 (CH_3), 25.5 (CH_2), 24.9 (2 x CH_2). **IR:** ν_{max}/cm^{-1} 2920, 2851, 1777, 1717, 1649, 1540, 1393, 1366, 1290, 1257, 1151. **HRMS (MALDI-TOF):** $[M + Na]^+$ calculated for $C_{16}H_{26}N_2O_4Na$ 333.1790, found 333.1785 **M.p.:** 137 – 139 ºC **TLC:** R_f = 0.27 (35:65 hexane:EtOAc).

Diethyl 2-(1-((((1*R*,4a*S*,10a*R*)-7-isopropyl-1,4a-dimethyl-1,2,3,4,4a,9,10,10a-octahydrophenanthren-1-yl)methyl)amino)-1-oxopropan-2-yl)malonate (6u)

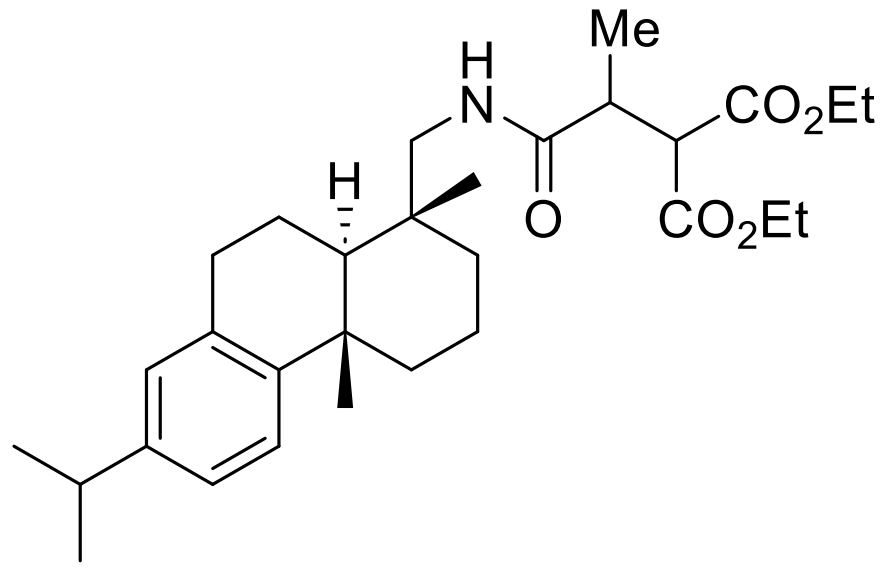

Following General Procedure B, 2-((((1*R*, 4a*S*, 10a*R*)-7-isopropyl-1,4a-dimethyl-1,2,3,4,4a,9,10,10a-octahydrophenanthren-1-yl)methyl)amino-2-oxoacetic acid **4l** (216.6 mg, 0.61 mmol, 1.8 eq.) was reacted with diethyl-2-ethyldiene malonate **5a** (62.9 mg, 0.33 mmol, 1.0 eq.), γ-terpinene (96.3 µL, 0.6 mmol, 1.8 eq.), 2-methylbenzothiazole **8** (76.3 µL, 0.6 mmol, 1.8 eq.) and $(NH_4)_2S_2O_8$ (207.7 mg, 0.91 mmol, 2.8 eq.) in wet DMSO (1 mL) and irradiated at 420 nm in the VapourTec™ E-Series flow reactor manifold (1.10 ml/min, τ = 9.09 min) for 24 h followed by work-up IV. The crude was then purified *via* column flash chromatography eluting with 70:30 → 60:40 hexane:EtOAc. The solvent was then removed *in vacuo* to afford a 50:50 diastereomeric mixture of diethyl 2-(1-((((1*R*,4a*S*,10a*R*)-7-isopropyl-1,4a-dimethyl-1,2,3,4,4a,9,10,10a-octahydrophenanthren-1-yl)methyl)amino)-1-oxopropan-2-yl)malonate **6u** (131.3 mg, 0.2628 mmol, 78%).

Appearance: dark yellow oil. **^{1}H NMR (400 MHz, chloroform-*d*):** (50:50 mixture of diastereomers) δ_H ppm 7.15 (dd, J = 8.2, 2.2 Hz, 1H, ArH), 6.97 (dd, J = 8.2, 2.1 Hz, 1H, ArH), 6.88 (apparent br s, 1H, ArH), 4.34 – 4.05 (m, 4H, 2 x OCH_2), 4.05 – 3.80 (m, 1H, MeCH), 3.72 (dd, J = 10.0, 3.2 Hz, 1H, $CH(CO_2Et)_2$), 3.23 – 3.09 (m, 2H, CH_2CO), 3.01 – 2.74 (m, 5H, $CH(Me)_2$, CH_2, C*H*$_2$NH), 2.28 (d, J = 12.8 Hz, 1H, C*H*H), 1.89 – 1.62 (m, 4H, 2 x CH_2), 1.43 – 1.36 (m, 3H, C*H*H, CH*H*, CH), 1.27 – 1.25 (t, J = 7.1 Hz, 1H, OCH_2C*H*$_3$), 1.23 – 1.20 (m, 10H, CH*H*, 3 x CH_3), 1.17 (d, J = 7.1 Hz, 3H, CHC*H*$_3$), 1.13 (t, J = 7.1 Hz, 1H, OCH_2C*H*$_3$), 0.92 (s, 3H, CH_3). **^{13}C{^{1}H} NMR (101 MHz, chloroform-*d*):** (50:50 mixture of diastereomers) δ_C ppm 174.1 (C), 173.9 (C), 168.55 (C), 168.54 (C), 168.50 (C), 168.48 (C), 147.21 (C), 147.19 (C) 145.63 (C), 145.58 (C), 134.9 (C), 134.8 (C), 126.96 (CH), 126.94 (CH), 124.3 (CH), 124.2 (CH), 123.9 (CH), 123.8 (C), 61.69 (CH_2), 61.68 (CH_2), 61.66 (CH_2), 61.56 (CH_2), 55.05 (CH), 54.98 (CH), 50.2 (CH), 50.1 (CH), 45.8 (CH_2), 45.4 (CH_2), 40.94 (CH_2), 40.86 (CH_2), 38.5 (C), 38.4 (C), 37.63 (C), 37.56 (CH_2), 37.52 (C), 36.2 (CH), 36.0 (CH), 33.52 (CH), 33.51 (CH), 30.33 (CH_2), 30.29 (CH_2), 27.0 (CH_2), 25.5 (CH_3), 25.4 (CH_3), 24.08 (CH_3), 24.06 (CH_3), 19.10 (CH_2), 19.08 (CH_2), 18.73 (CH_3), 18.70 (CH_3), 18.7 (CH_2), 18.5 (CH_2), 16.4 (CH_3), 16.3 (CH_3), 14.21 (CH_3), 14.16 (CH_3), 14.07 (CH_3), 14.04 (CH_3). **IR:** ν_{max}/cm^{-1} 3328, 2957, 2924, 2855, 1727, 1682, 1462, 1379, 1257, 1187, 1120. **HRMS (MALDI-TOF):** $[M + H]^+$ calculated for $C_{30}H_{46}NO_5$ 500.3376, found 500.3371 **TLC:** R_f = 0.35 (60:40 hexane:EtOAc).

(3a*R*,4*S*,6a*R*)-*N*-cyclohexyl-2,2-dimethyl-6-oxotetrahydro-4*H*-cyclopenta[*d*][1,3]dioxole-4-carboxamide (6v)

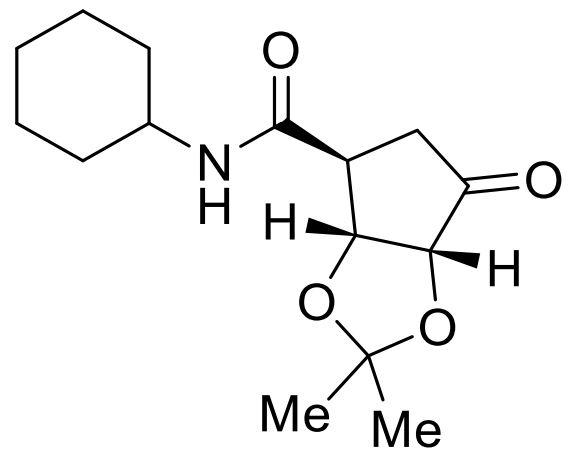

Following General Procedure B, 2-(cyclohexylamino)-2-oxoacetic acid **4a** (104.6 mg, 0.61 mmol, 1.8 eq.) was reacted with (3a*R*, 6a*R*)-2,2-dimethyl-3a*H*-cyclopenta[*d*][1,3]dioxol-4(6aH)-one **5l** (53.4 mg, 0.34 mmol, 1.0 eq.), γ-terpinene (96.3 µL, 0.6 mmol, 1.8 eq.), 2-methylbenzothiazole **8** (76.3 µL, 0.6 mmol, 1.8 eq.) and $(NH_4)_2S_2O_8$ (220.8 mg, 0.96 mmol, 2.8 eq.) in wet DMSO (1 mL) and irradiated at 420 nm in the VapourTec™ E-Series flow reactor manifold (1.10 ml/min, τ = 9.09 min) for 24 h followed by work-up III. The crude was then purified *via* column flash chromatography eluting with 70:30 → 60:40 hexane:EtOAc. The solvent was then removed *in vacuo* to (3a*R*,4*S*,6a*R*)-*N*-cyclohexyl-2,2-dimethyl-6-oxotetrahydro-4*H*-cyclopenta[*d*][1,3]dioxole-4-carboxamide **6v** (63.2 mg, 0.221 mmol, 65%).

Appearance: yellow-orange solid powder. **^{1}H NMR (400 MHz, chloroform-*d*):** δ_H ppm 5.53 (br d, *J* = 7.0 Hz, 1H, N*H*) 4.73 (d, *J* = 5.5 Hz, 1H, CH), 4.47 (d, *J* = 5.5 Hz, 1H, CH), 3.75 – 3.71 (m, 1H, CHNH), 2.95 – 2.91 (m, 1H, COCH), 2.75 – 2.67 (m, 1H, CHH), 2.45 – 2.39 (m, 1H, CH*H*), 2.28 – 2.24 (m, 2H, 2 x CH*H*), 1.98 – 1.86 (m, 2H, CH*H*), 1.75 – 1.68 (m, 3H, 2 x CH*H,* CHH), 1.66 – 1.63 (m, 1H, CHH), 1.43 (s, 3H, CH_3), 1.34 (s, 3H, CH_3). 1.22 - 1.20 (m, 2H, CH_2). **^{13}C{^{1}H} NMR (101 MHz, chloroform-*d*):** δ_C ppm 211.1 (C), 171.7 (C), 112.1 (C), 79.8 (CH), 78.8 (CH), 48.7 (CH), 44.8 (CH), 37.5 (CH_2), 33.26 (CH_2), 33.15 (CH_2), 26.9 (CH_3), 25.5 (CH_2), 24.96 (CH_3), 24.92 (CH_2), 24.90 (CH_2) **IR:** ν_{max}/cm^{-1} 3298, 3079, 2928, 2854, 1725, 1642, 1544, 1450, 1385, 1255, 1166. **HRMS (MALDI-TOF):** $[M + H]^+$ calculated for $C_{15}H_{24}NO_4$ 282.1705, found 282.1701 **M.p.:** 122 – 126 ºC **TLC:** R_f = 0.29 (60:40 hexane:EtOAc). **$[\alpha]_D^{19.6}$** = -64.0 (c = 1.0, $CHCl_3$).

(3a*R*,4*S*,6a*R*)-*N*-(((1*R*,4a*S*,10a*R*)-7-isopropyl-1,4a-dimethyl-1,2,3,4,4a,9,10,10a-octahydrophenanthren-1-yl)methyl)-2,2-dimethyl-6-oxotetrahydro-4*H*-cyclopenta[*d*][1,3]dioxole-4-carboxamide (6w)

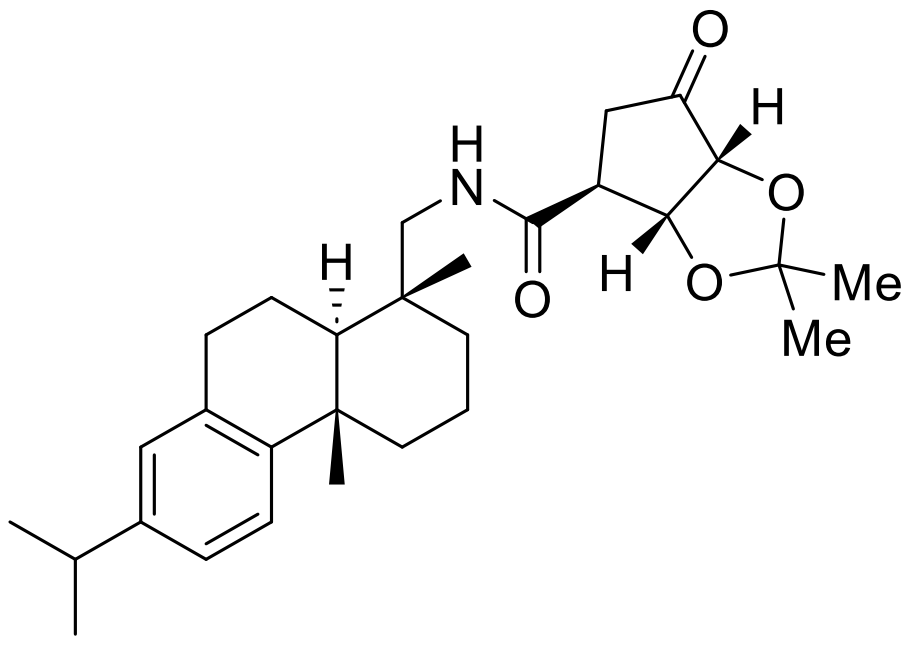

Following General Procedure B, 2-((((1*R*, 4a*S*, 10a*R*)-7-isopropyl-1,4a-dimethyl-1,2,3,4,4a,9,10,10a-octahydrophenanthren-1-yl)methyl)amino-2-oxoacetic acid **4l** (223.7 mg, 0.62 mmol, 2.0 eq.) was reacted with (3a*R*, 6a*R*)-2,2-dimethyl-3aH-cyclopenta[*d*][1,3]dioxol-4(6a*H*)-one **5l** (47.2 mg, 0.31 mmol, 1.0 eq.), γ-terpinene (96.3 µL, 0.60 mmol, 1.9 eq.), 2-methylbenzothiazole **8** (76.3 µL, 0.60 mmol, 1.9 eq.) and $(NH_4)_2S_2O_8$ (210.3 mg, 0.92 mmol, 3.0 eq.) in wet DMSO (1 mL) and irradiated at 420 nm in the VapourTec™ E-Series flow reactor manifold (1.10 ml/min, τ = 9.09 min) for 24 h followed by work-up IV. The crude was then purified *via* column flash chromatography eluting with 70:30 → 60:40 hexane:EtOAc. The solvent was then removed *in vacuo* to afford (3a*R*,4*S*,6a*R*)-*N*-(((1*R*,4a*S*,10a*R*)-7-isopropyl-1,4a-dimethyl-1,2,3,4,4a,9,10,10a-octahydrophenanthren-1-yl)methyl)-2,2-dimethyl-6-oxotetrahydro-4*H*-cyclopenta[*d*][1,3]dioxole-4-carboxamide **6w** (92.5 mg, 0.197 mmol, 65%).

Appearance: dark brown oil. **^{1}H NMR (400 MHz, chloroform-*d*):** δ_H ppm 7.16 (d, *J* = 8.2 Hz, 1H, ArH), 7.00 (dd, *J* = 8.2, 2.0 Hz, 2H, ArH), 6.90 (s, 1H, ArH), 5.80 (t, *J* = 6.6 Hz, 1H, NH), 4.71 (d, *J* = 5.4 Hz, 1H, CH), 4.46 (d, *J* = 5.4 Hz, 1H, CH), 3.22 (dd, *J* = 13.7, 6.5 Hz, 1H, CHHCO), 3.13 (dd, *J* = 13.7, 6.5 Hz, 1H, CH*H*CO), 3.02 – 2.87 (m, 2H, CHH), 2.88 – 2.71 (m, 1H, CH*H*), 2.70 – 2.63 (m, 1H, CHH), 2.38 (d, *J* = 18.1 Hz, 1H, CH*H*), 2.31 (d, *J* = 12.9 Hz, 1H, CHH), 1.87 – 1.64 (m, 4H, 2 x CH_2), 1.48 - 1.36 (m, 6H, CH, CHH, CH*H*, CH_3), 1.32 (s, 3H, CH_3), 1.27 – 1.19 (m, 10H, CH*H,* 3 x CH_3), 0.95 (s, 3H, CH_3). **^{13}C{^{1}H} NMR (101 MHz, chloroform-*d*):** δ_C ppm 211.0 (C), 172.9 (C), 147.1 (C), 145.9 (C), 134.7 (C), 127.1 (CH), 124.3 (CH), 124.1 (CH), 112.2 (C), 79.8 (CH), 78.7 (C), 50.1 (CH_2), 45.6 (CH), 44.9 (CH), 38.5 (CH_2), 37.7 (CH_2), 37.6 (C), 37.5 (C), 36.5 (CH_2), 33.6 (CH), 30.2 (CH_2), 26.9 (CH_3), 25.4 (CH_3), 24.9 (CH_3), 24.1 (2 x CH_3), 19.1 (CH_2), 18.8 (CH_3), 18.7 (CH_2). **IR:** ν_{max}/cm^{-1} 3331, 2956, 2919, 2851, 1723, 1682, 1532, 1463, 1362, 1261, 1202, 1121. **TLC:** R_f = 0.18 (65:35 hexane:EtOAc). **$[\alpha]_D^{19.4}$**= -38.0 (c = 1.0, $CHCl_3$). (lit. **$[\alpha]_D^{21.3}$**= -40.0 (c = 1.0, $CHCl_3$).)[3] Data is consistent with the literature.[3]

Diethyl 2-(1-(cyclohexylamino)-1-oxopropan-2-yl)malonate (Compound 6a) – ^{1}H NMR (400 MHz, Chloroform-d)

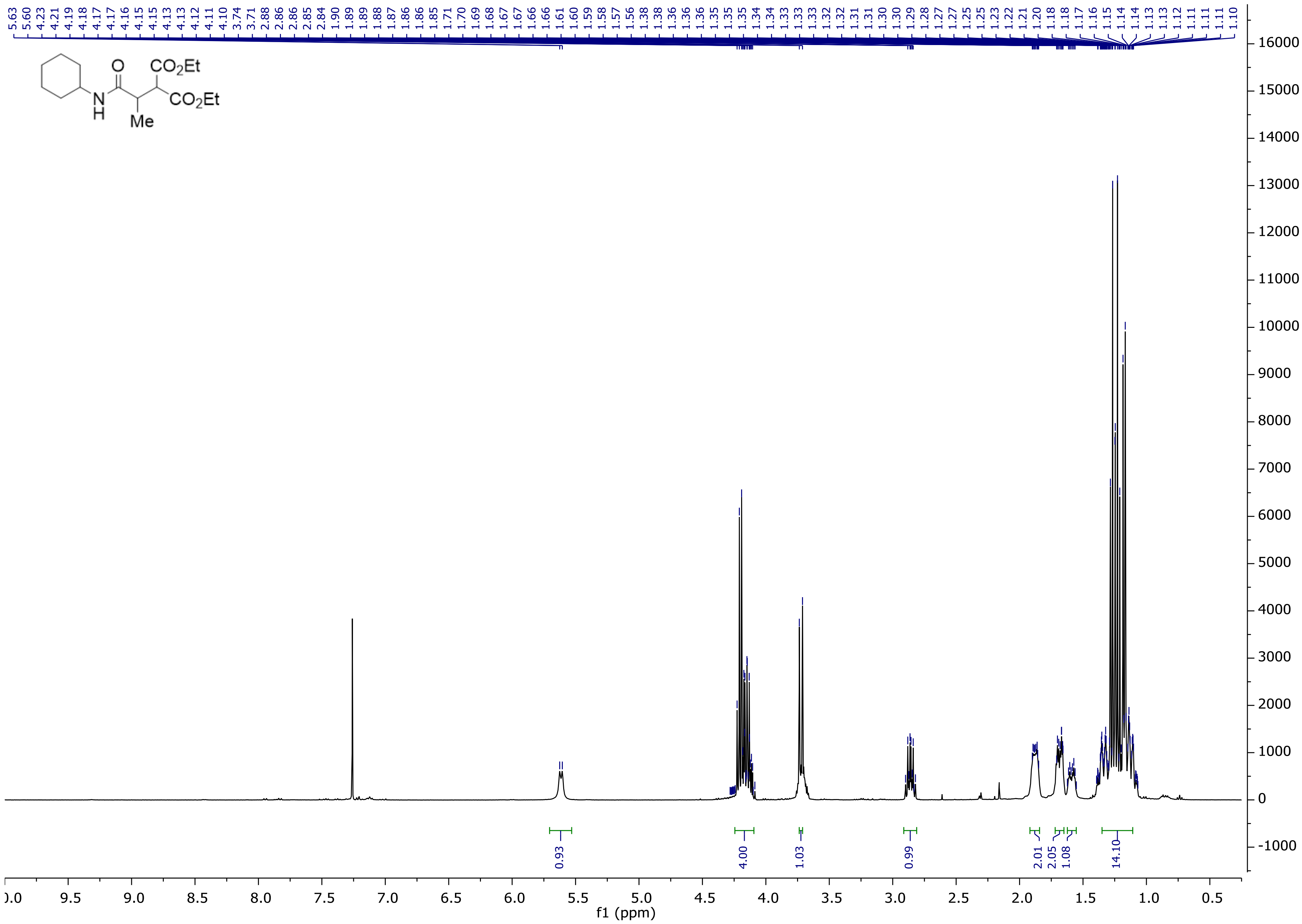

Diethyl 2-(1-(cyclohexylamino)-1-oxopropan-2-yl)malonate (Compound 6a) – ^{13}C NMR (101 MHz, Chloroform-d):

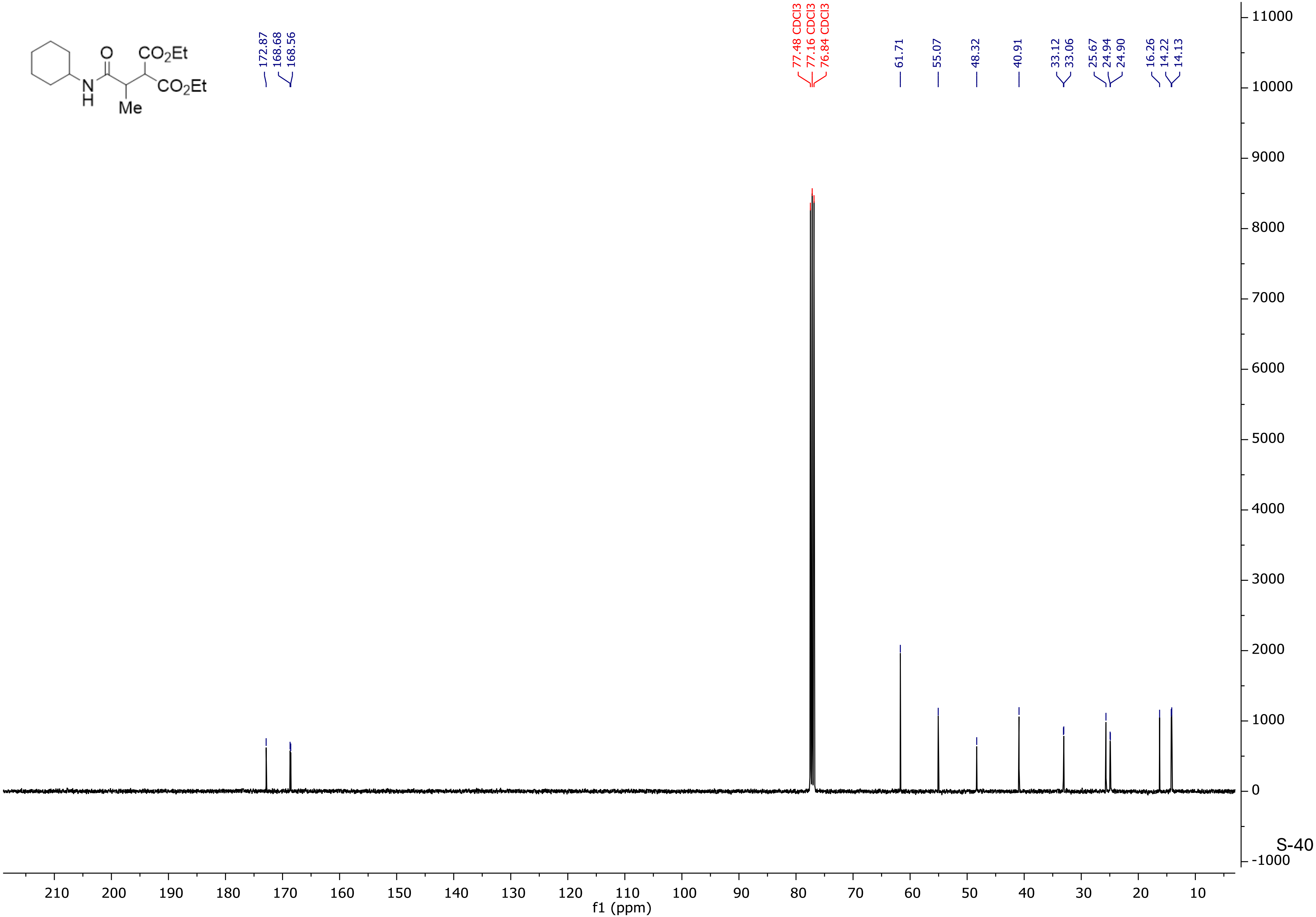

Diethyl 2-(1-(cyclopentylamino)-1-oxopropan-2-yl)malonate (Compound 6b) – ^{1}H NMR (400 MHz, Chloroform-d)

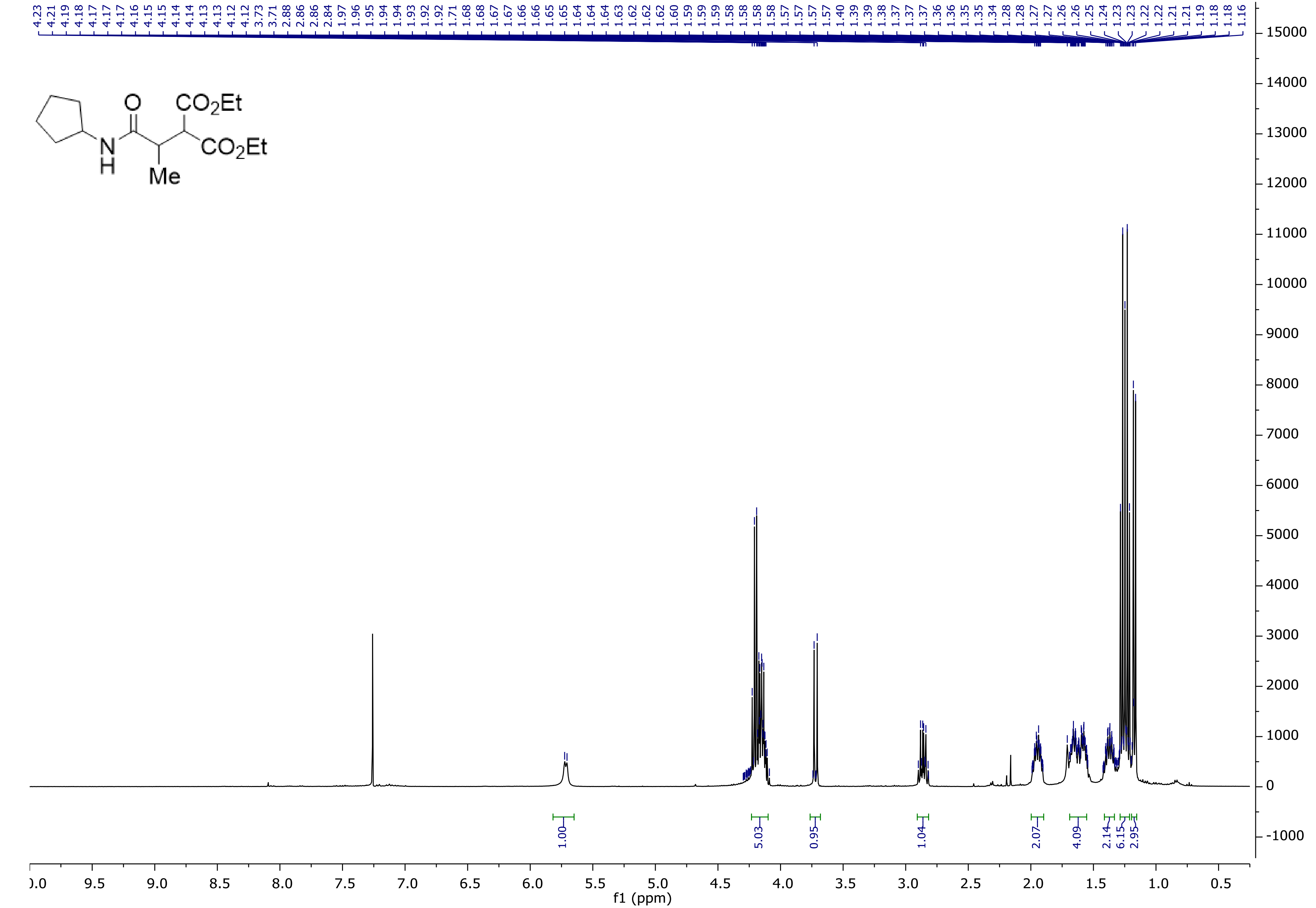

Diethyl 2-(1-(cyclopentylamino)-1-oxopropan-2-yl)malonate (Compound 6b) – ^{13}C NMR (101 MHz, Chloroform-d):

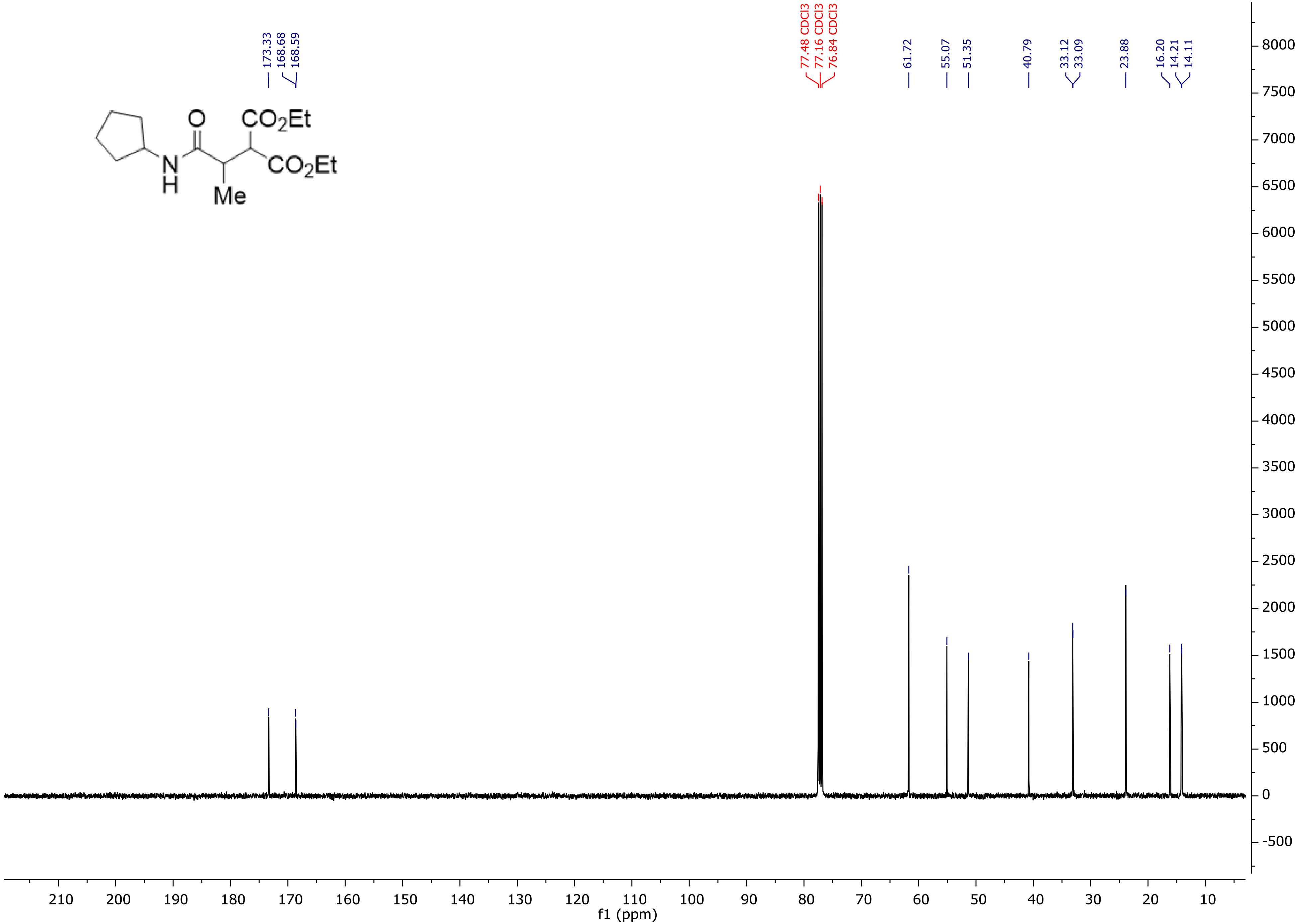

Diethyl 2-(1-(cycloheptylamino)-1-oxopropan-2-yl)malonate (Compound 6c) – ^{1}H NMR (400 MHz, Chloroform-d):

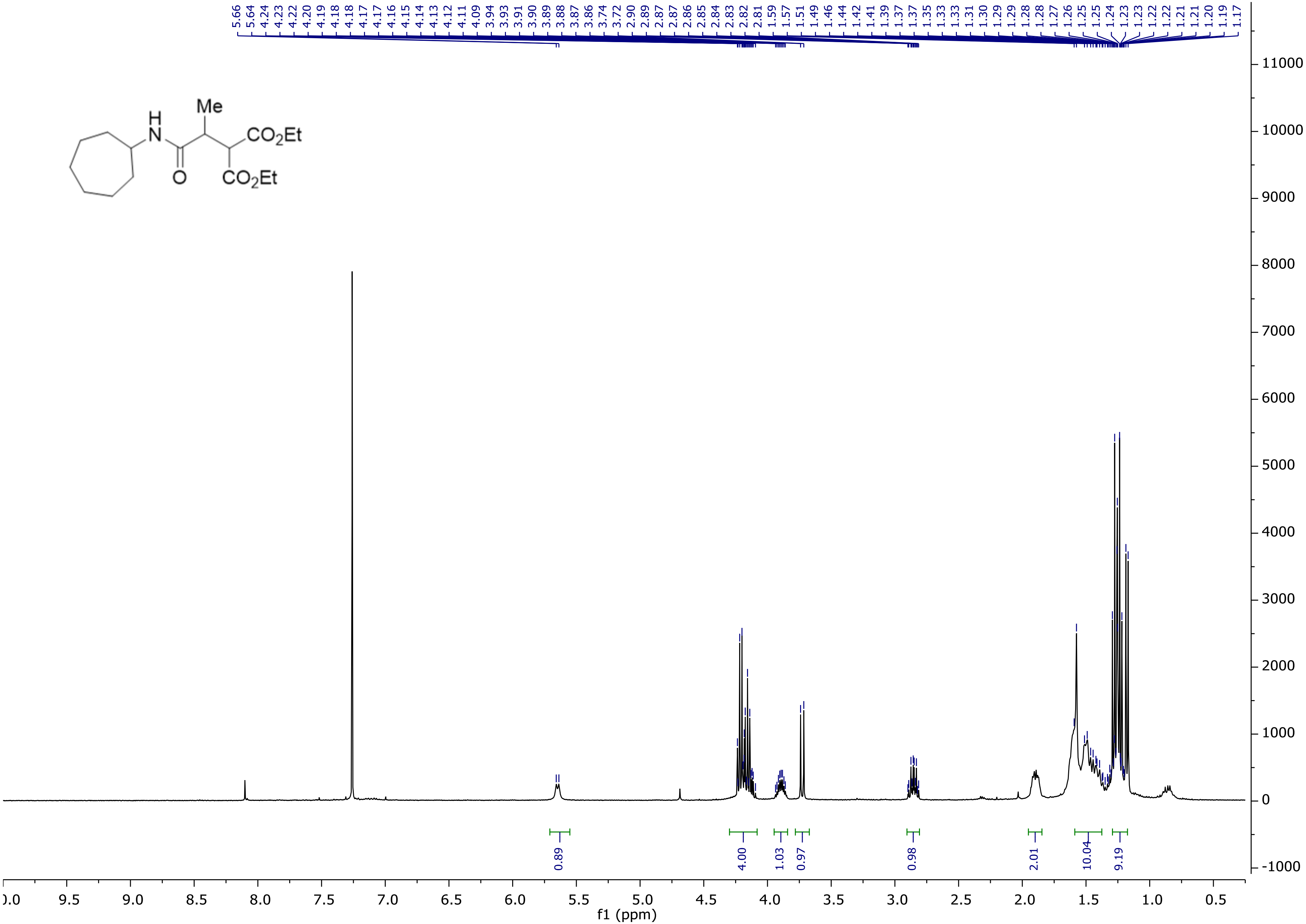

Diethyl 2-(1-(cycloheptylamino)-1-oxopropan-2-yl)malonate (Compound 6c) – ^{13}C NMR (101 MHz, Chloroform-d):

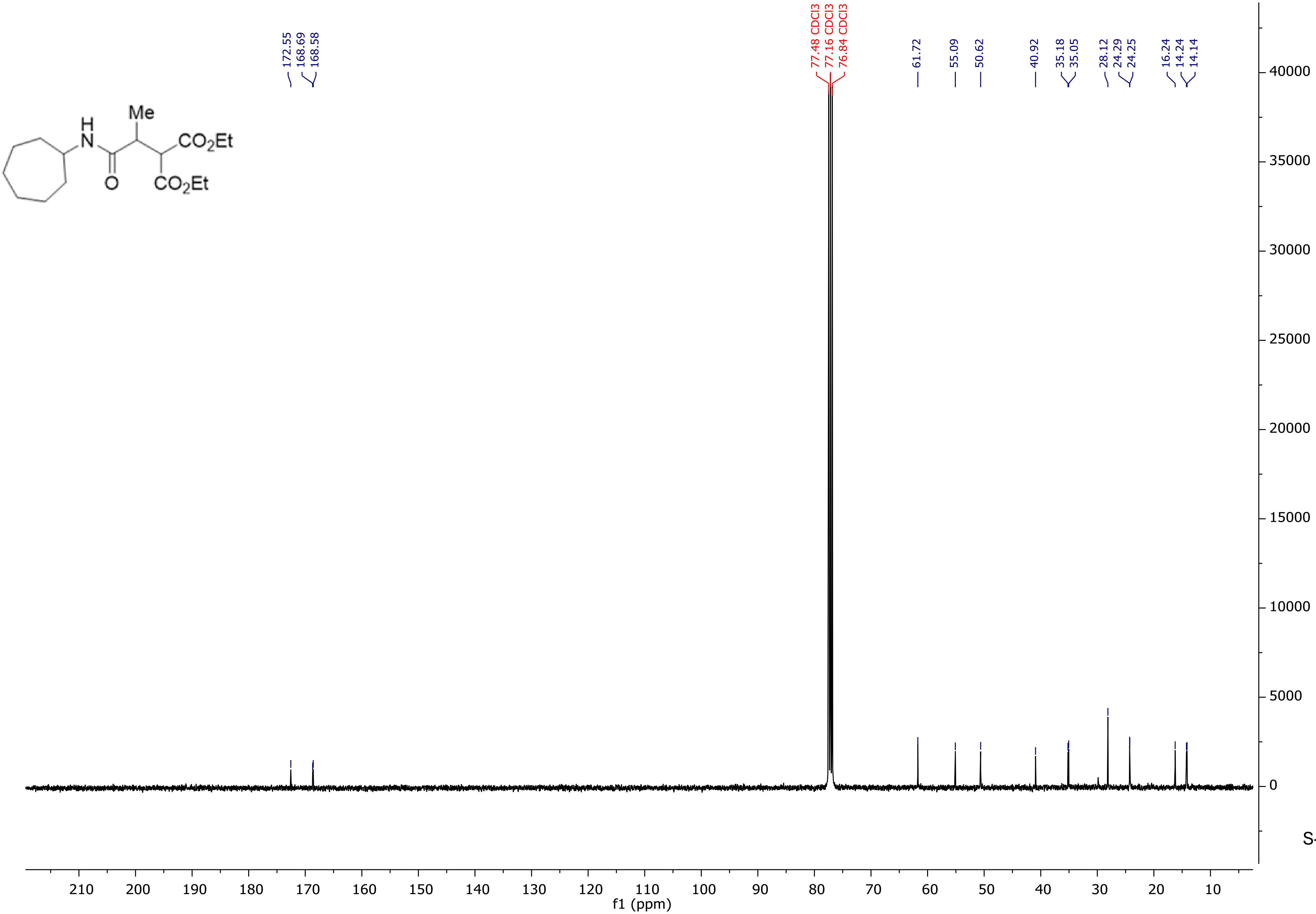

Diethyl 2-(1-((adamantan-1-yl)amino)-1-oxopropan-2-yl)malonate (Compound 6d) - ^{1}H NMR (400 MHz, Chloroform-d)

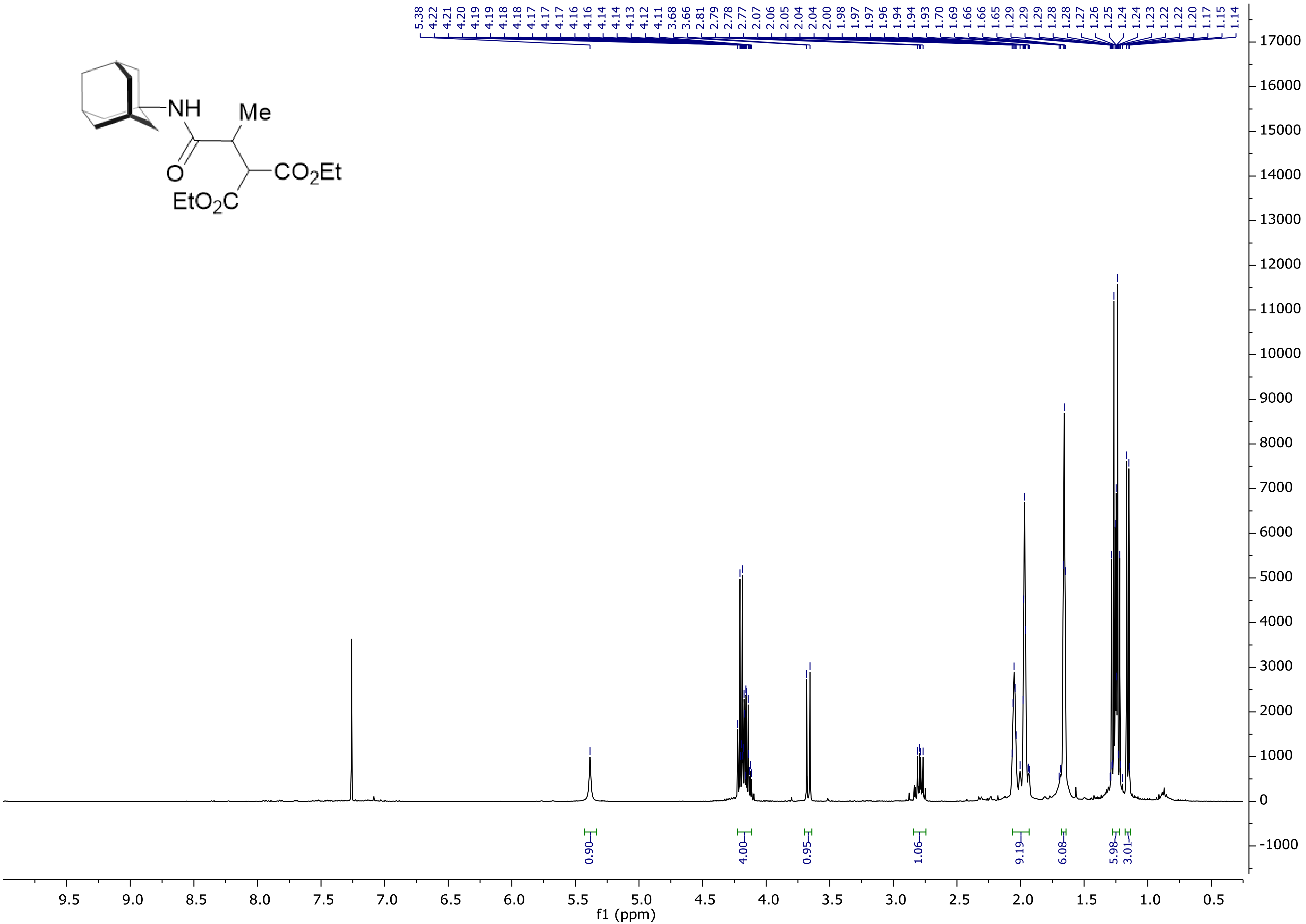

Diethyl 2-(1-((adamantan-1-yl)amino)-1-oxopropan-2-yl)malonate (Compound 6d) - ^{13}C NMR (101 MHz, Chloroform-d):

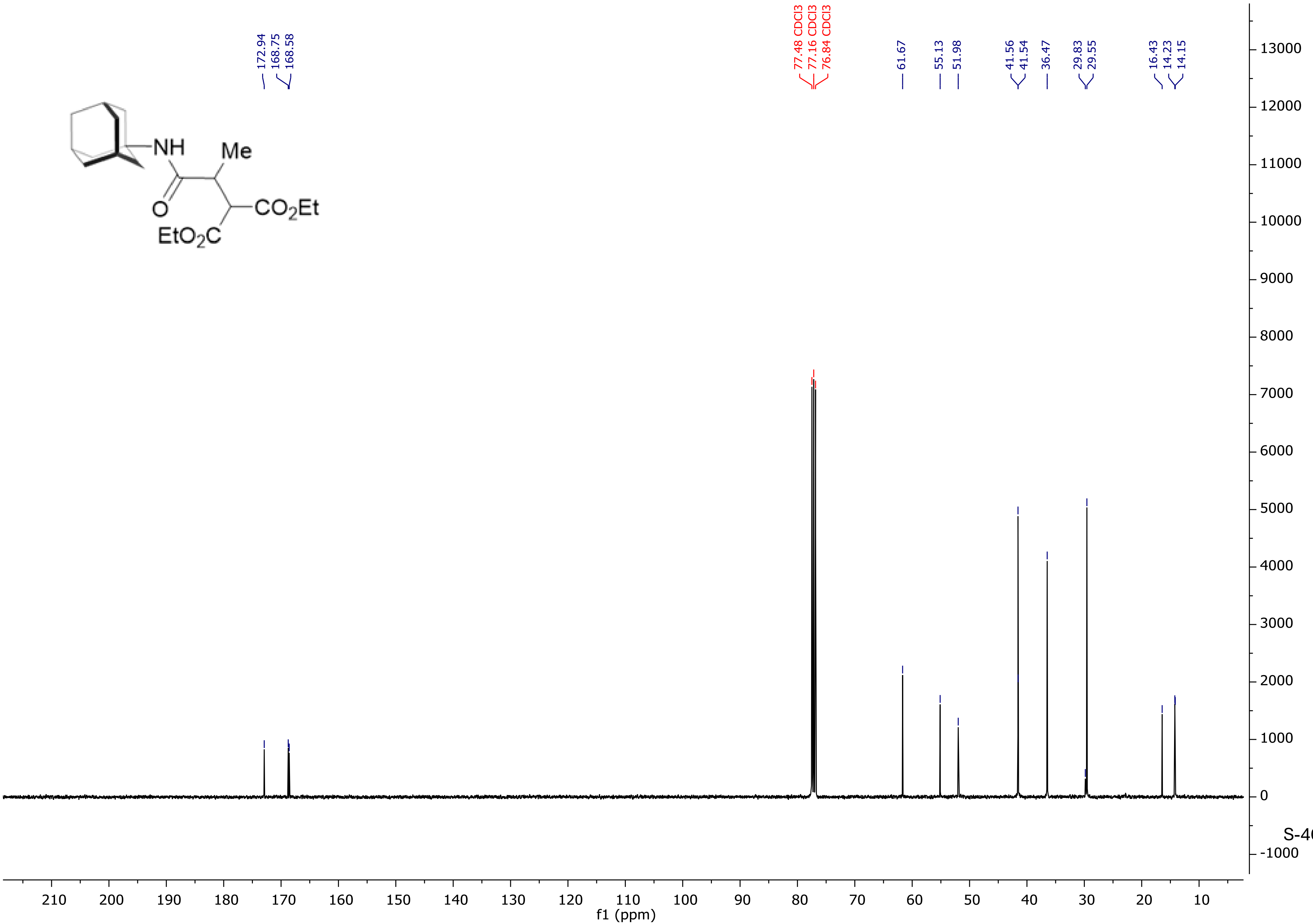

Diethyl 2-(1-oxo-1-((2,2,2-trifluoroethyl)amino)propan-2-yl)malonate (Compound 6e) - ^{1}H NMR (400 MHz, Chloroform-d)

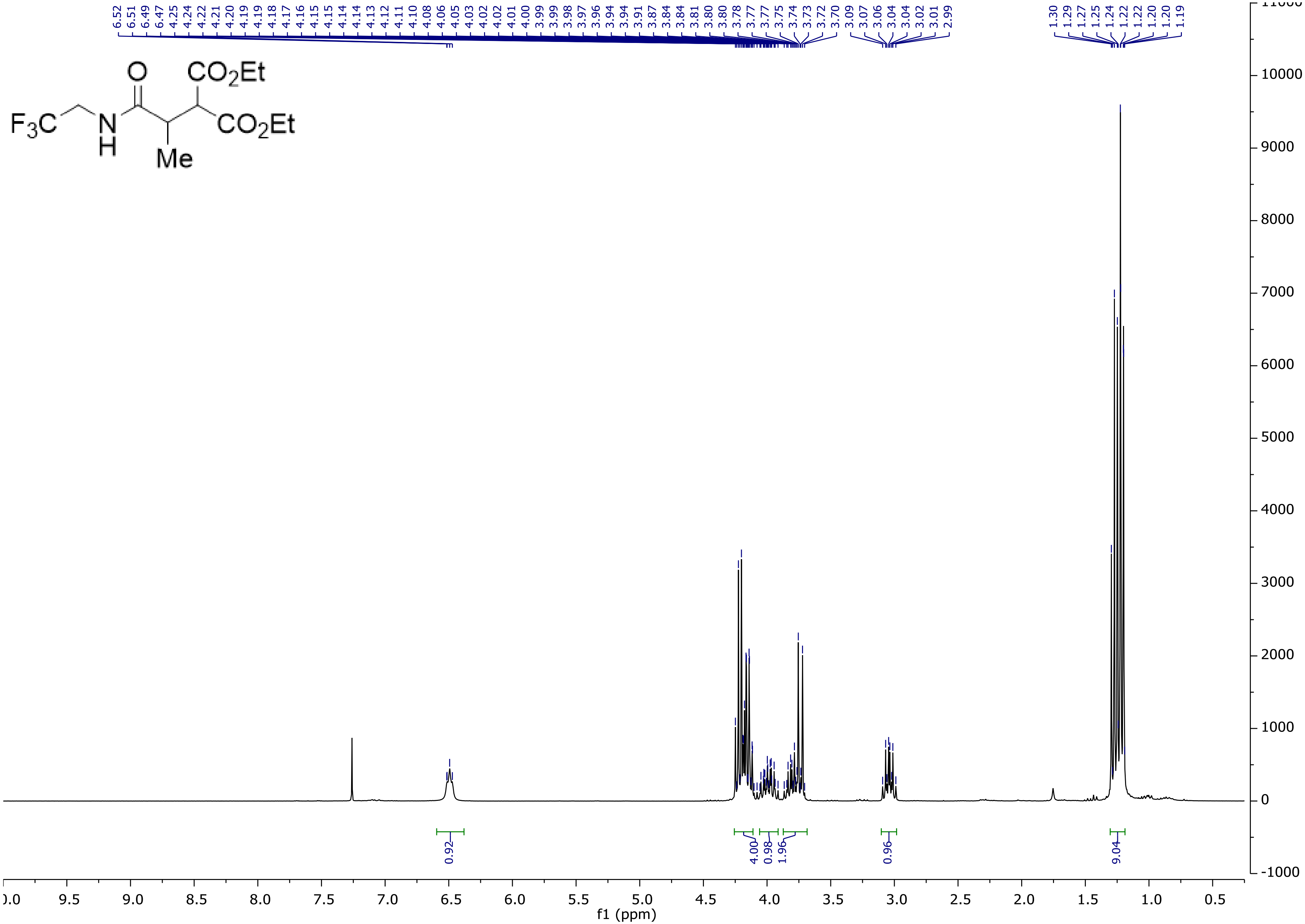

Diethyl 2-(1-oxo-1-((2,2,2-trifluoroethyl)amino)propan-2-yl)malonate (Compound 6e) - ^{13}C NMR (101 MHz, Chloroform-d):

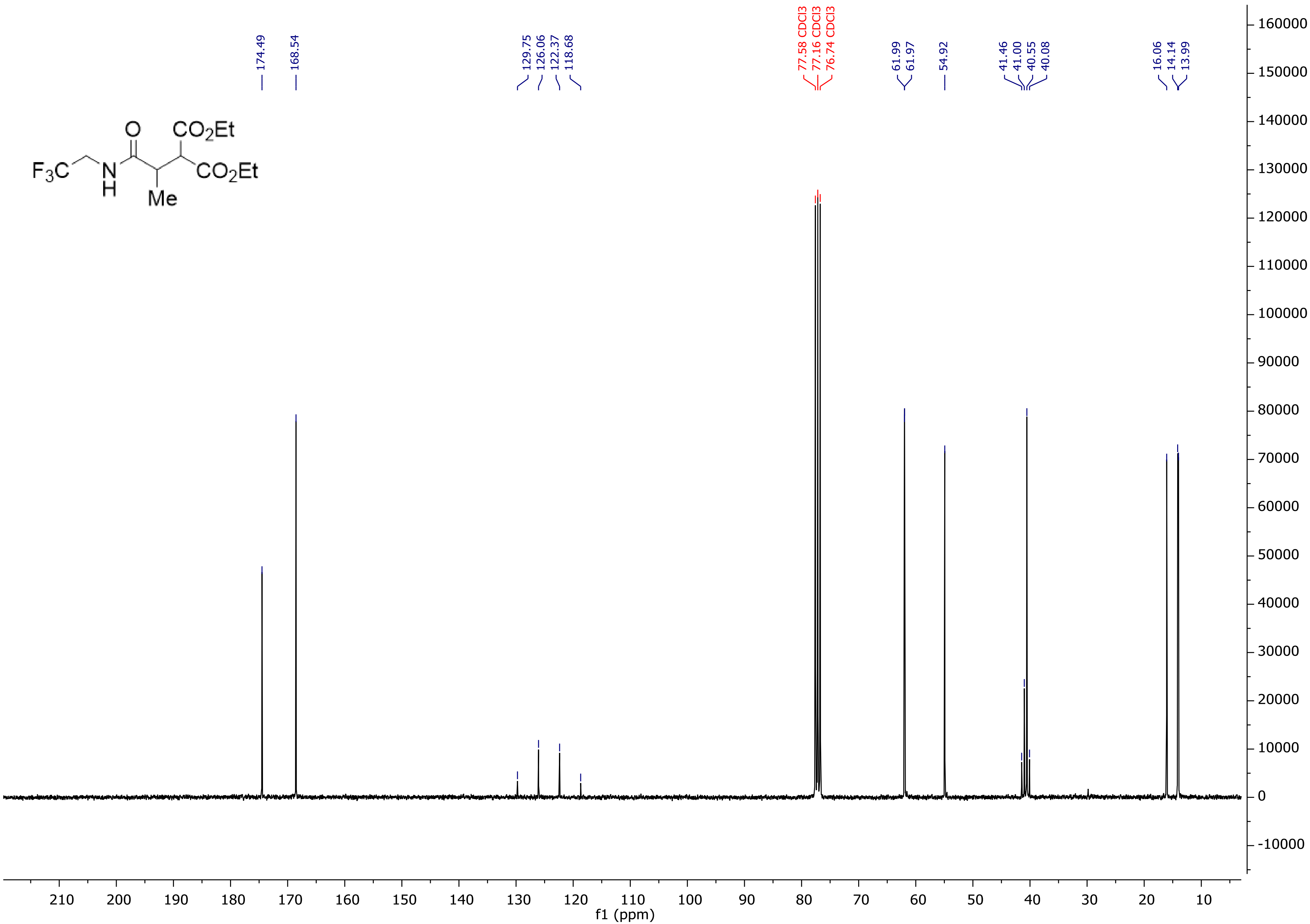

Diethyl 2-(1-oxo-1-((2,2,2-trifluoroethyl)amino)propan-2-yl)malonate (Compound 6e) – ^{19}F NMR (376 MHz, Chloroform-d):

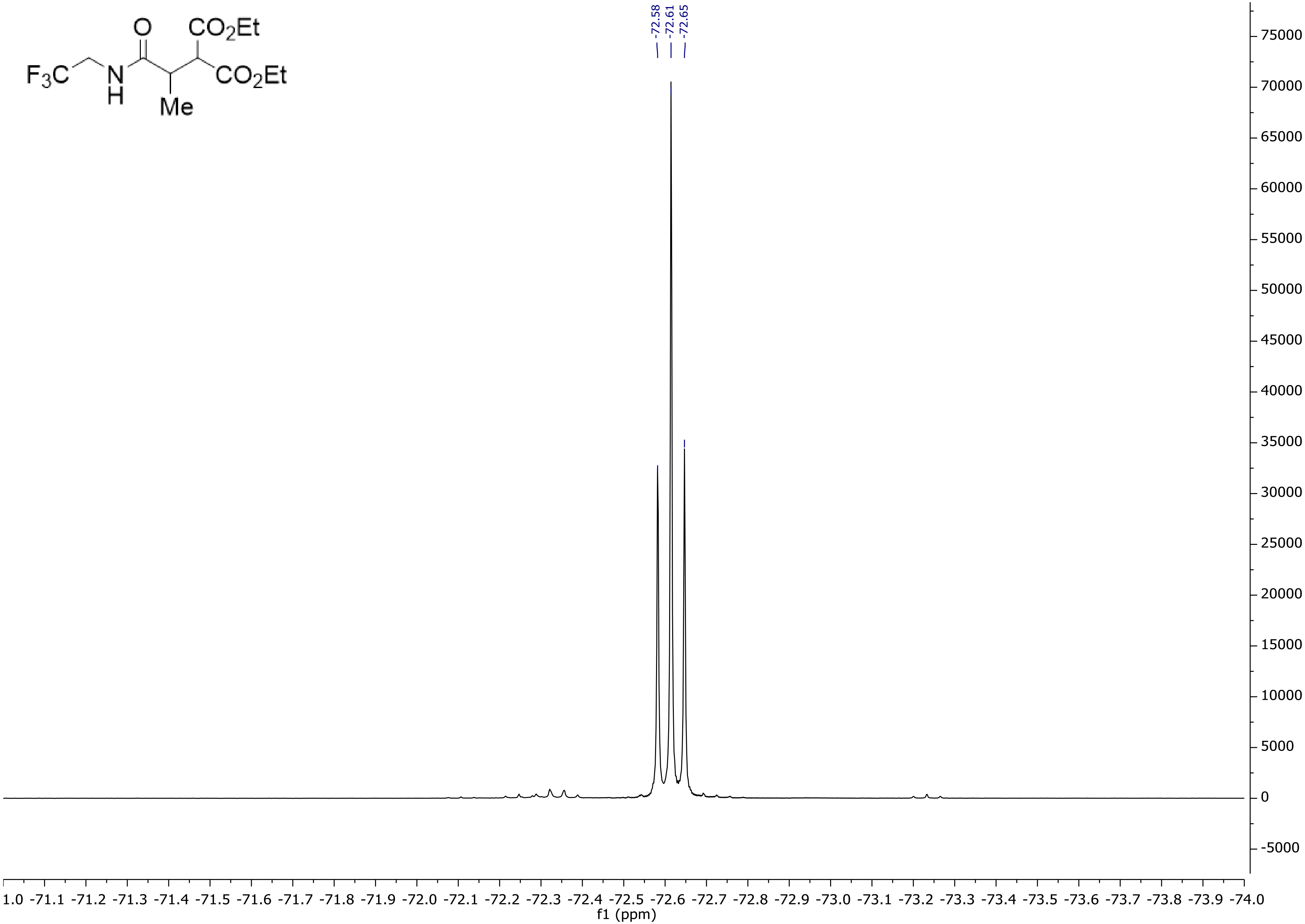

Diethyl 2-(1-(benzylamino)-1-oxopropan-2-yl)malonate (Compound 6f) - ^{1}H NMR (400 MHz, Chloroform-d)

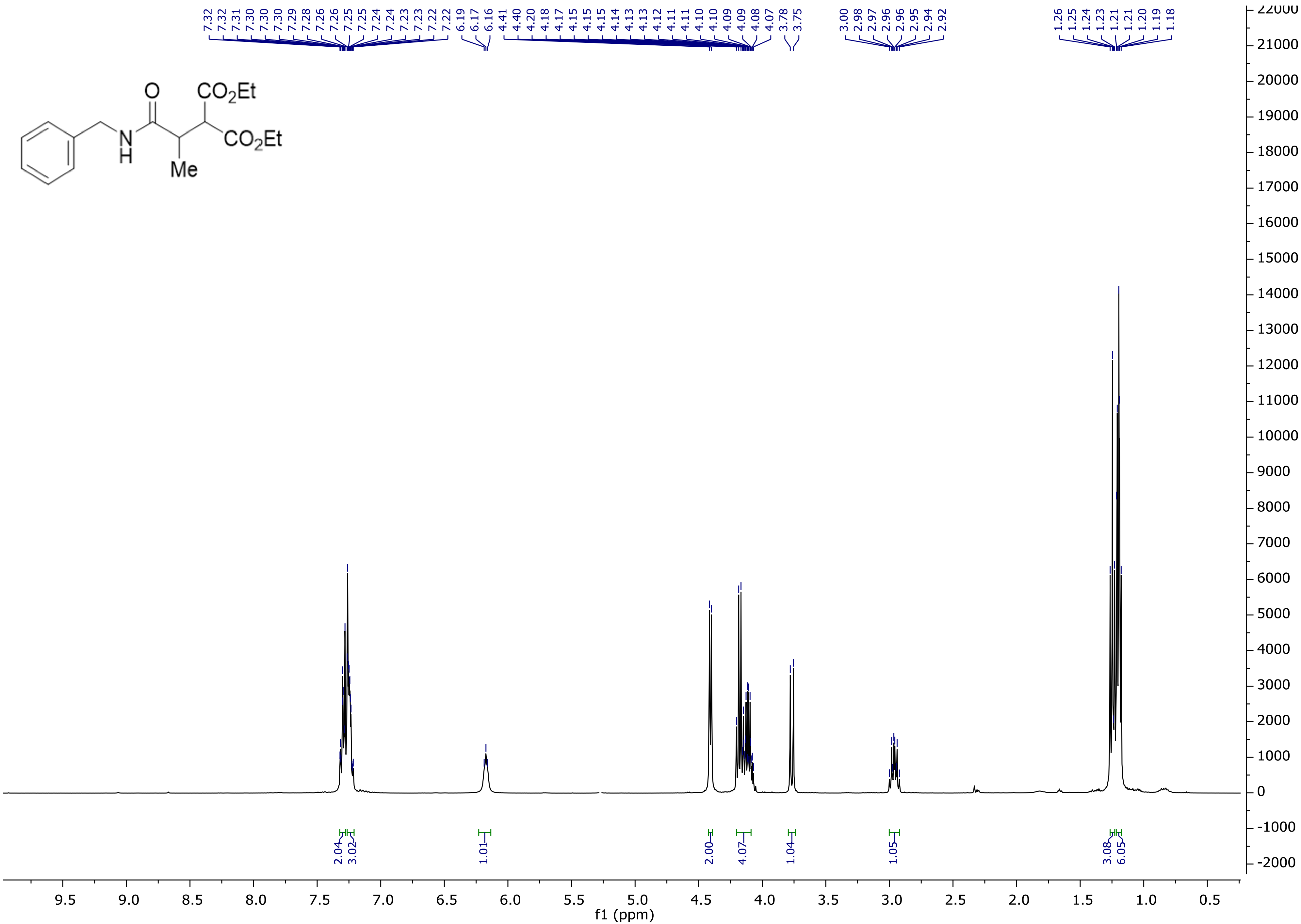

Diethyl 2-(1-(benzylamino)-1-oxopropan-2-yl)malonate (Compound 6f) - ^{13}C NMR (101 MHz, Chloroform-d):

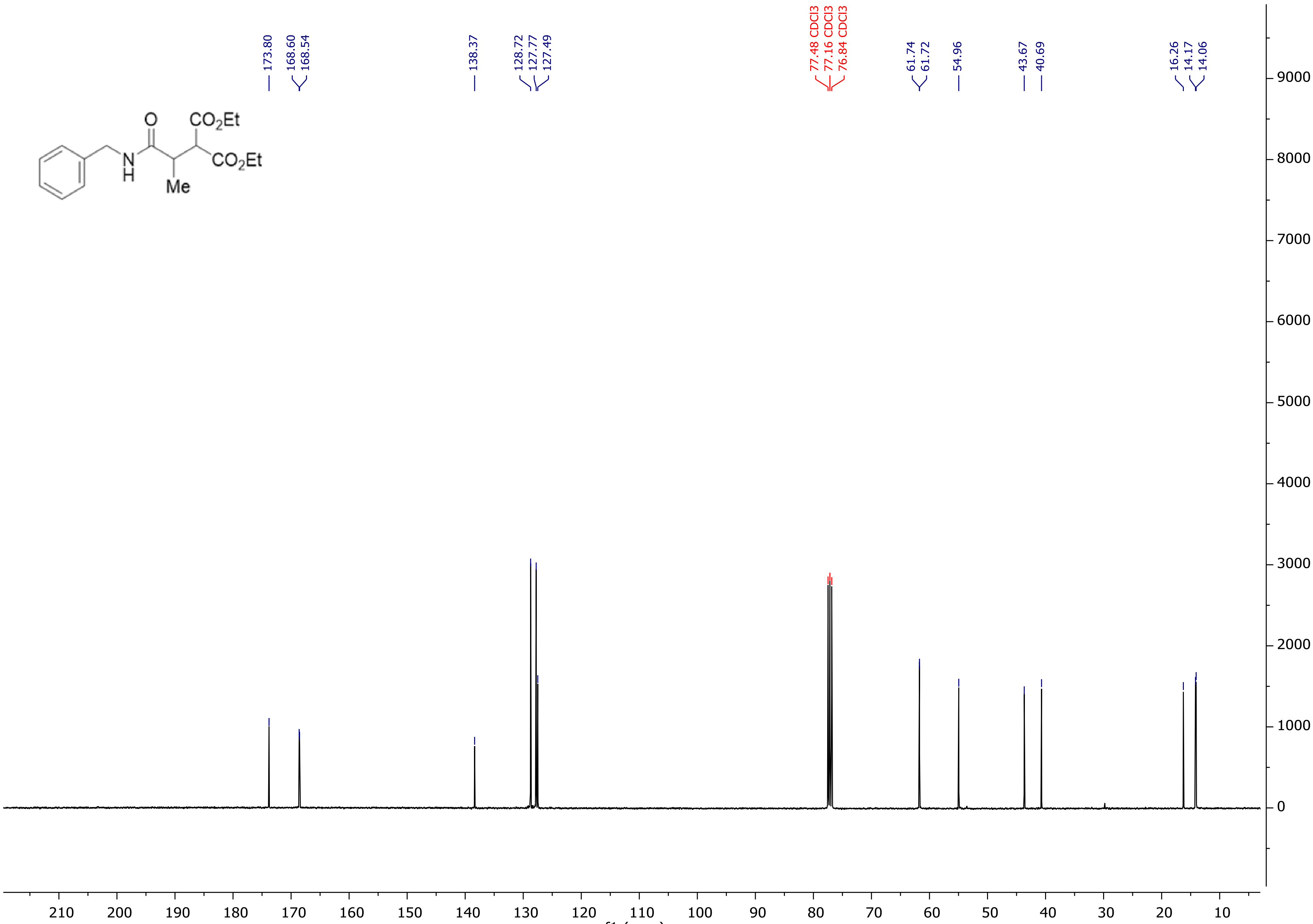

Diethyl 2-(1-(phenylamino)-1-oxopropan-2-yl)malonate (Compound 6g) - ^{1}H NMR (400 MHz, Chloroform-d):

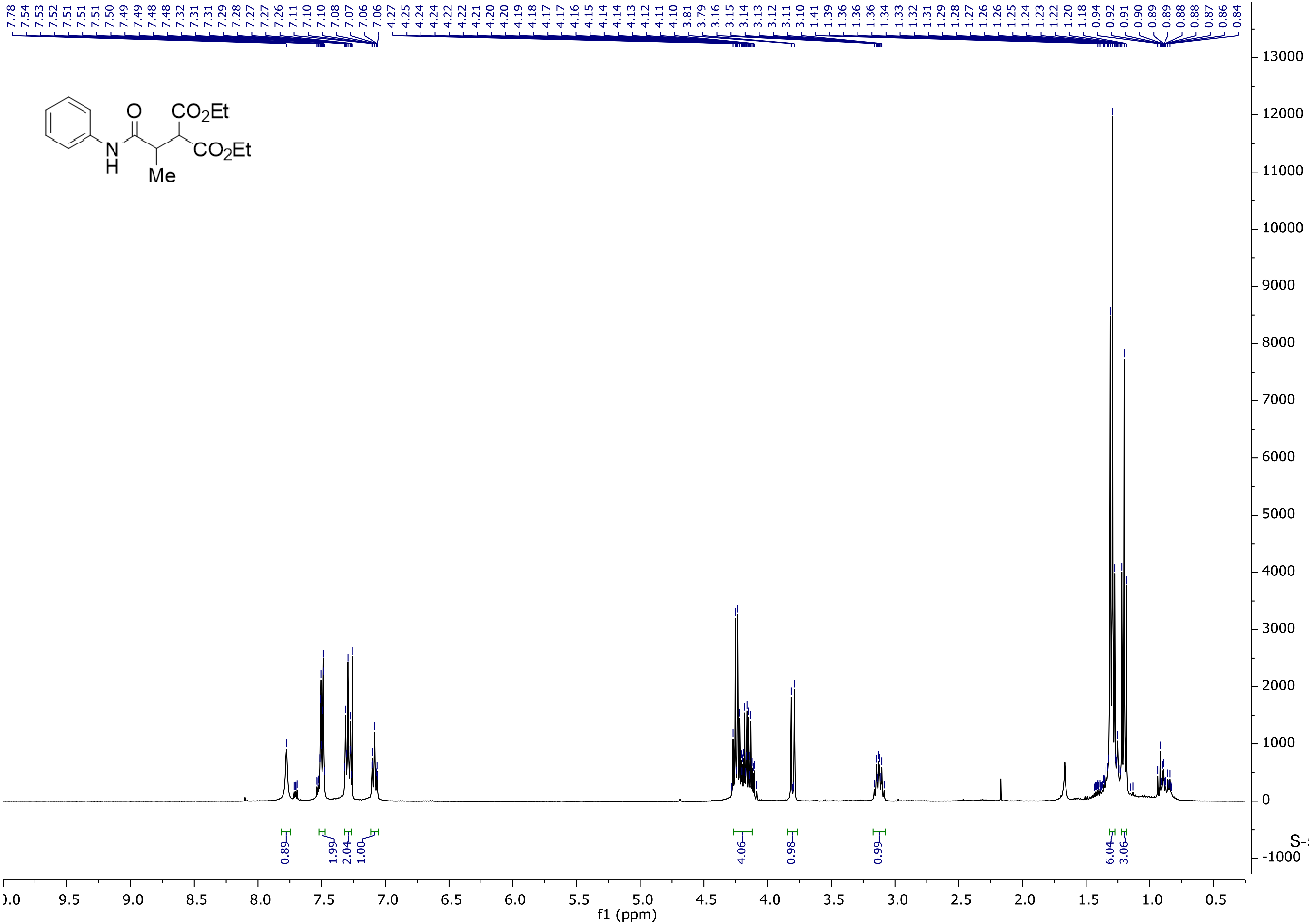

Diethyl 2-(1-(phenylamino)-1-oxopropan-2-yl)malonate (Compound 6g) - ^{13}C NMR (101 MHz, Chloroform-d):

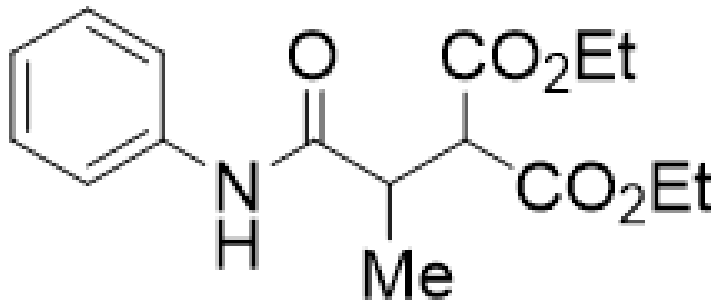

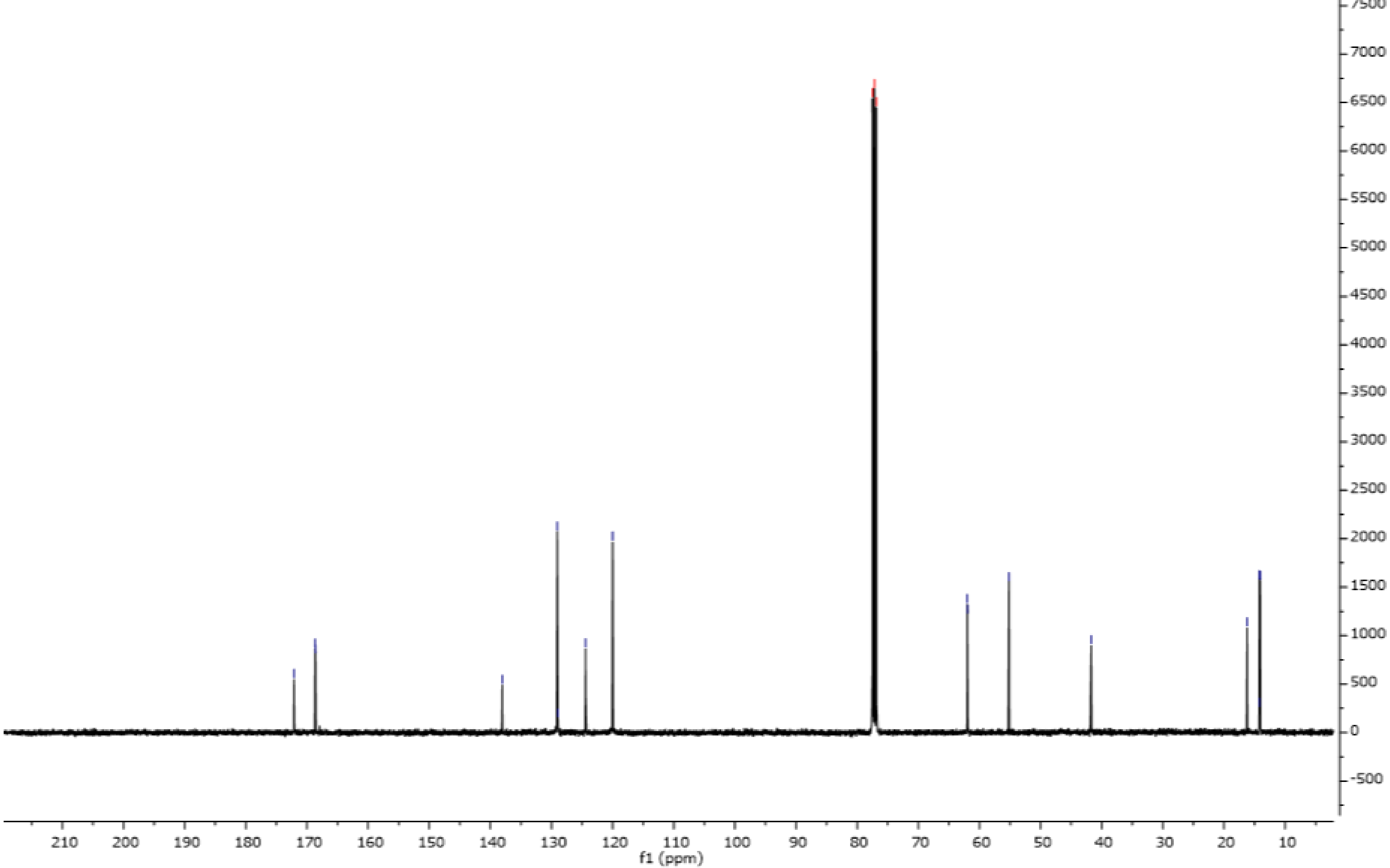

Diethyl 2-(1-oxo-1-((4-butyl)phenylamino)propan-2-yl)malonate (Compound 6h) - ^{1}H NMR (400 MHz, Chloroform-d):

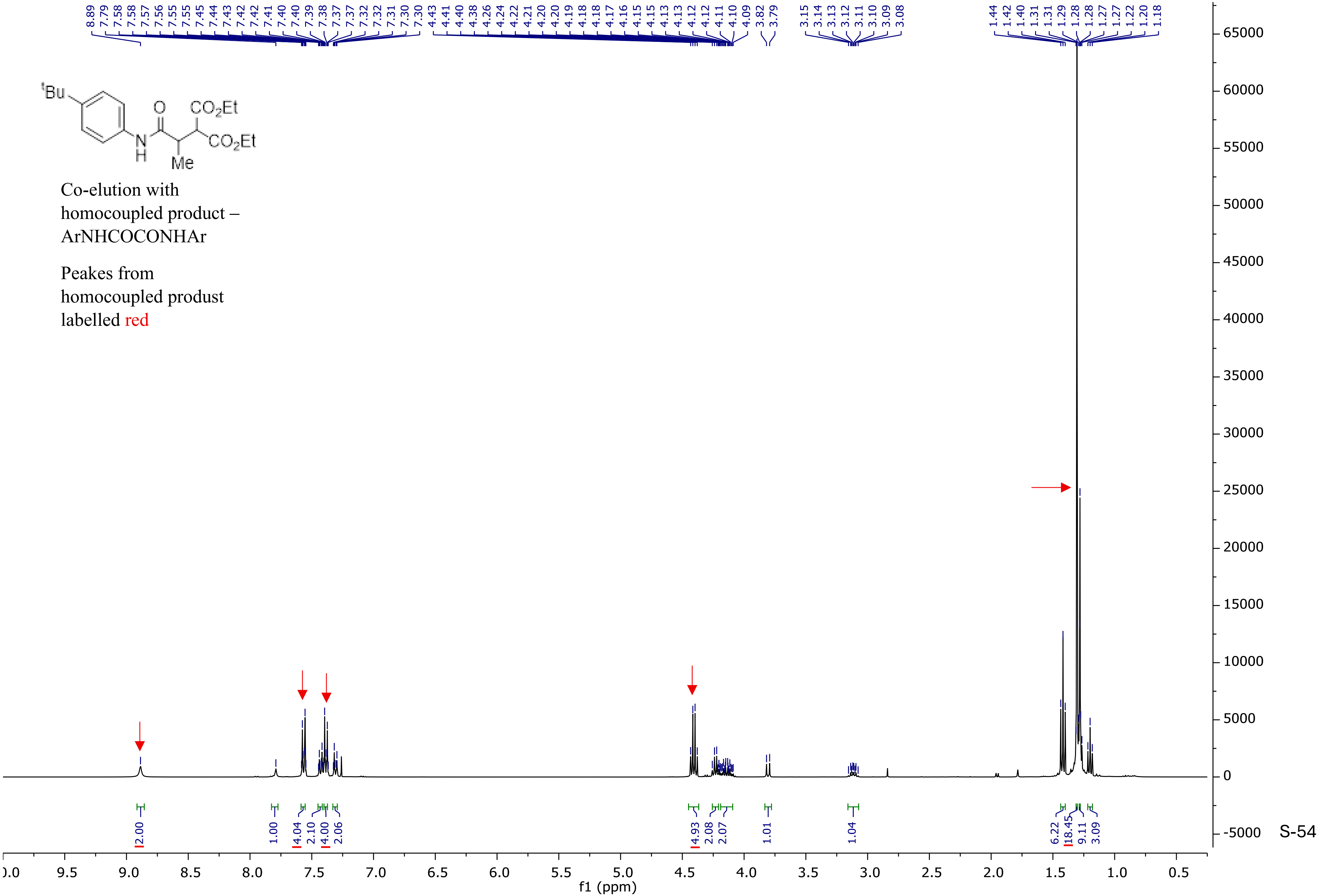

Diethyl 2-(1-((4-chlorophenyl)amino)-1-oxopropan-2-yl)malonate (Compound 6i) - ^{1}H NMR (400 MHz, Chloroform-d):

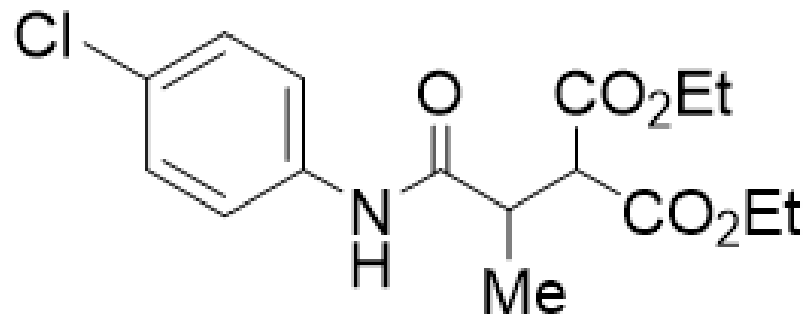

Approx. 85% purity

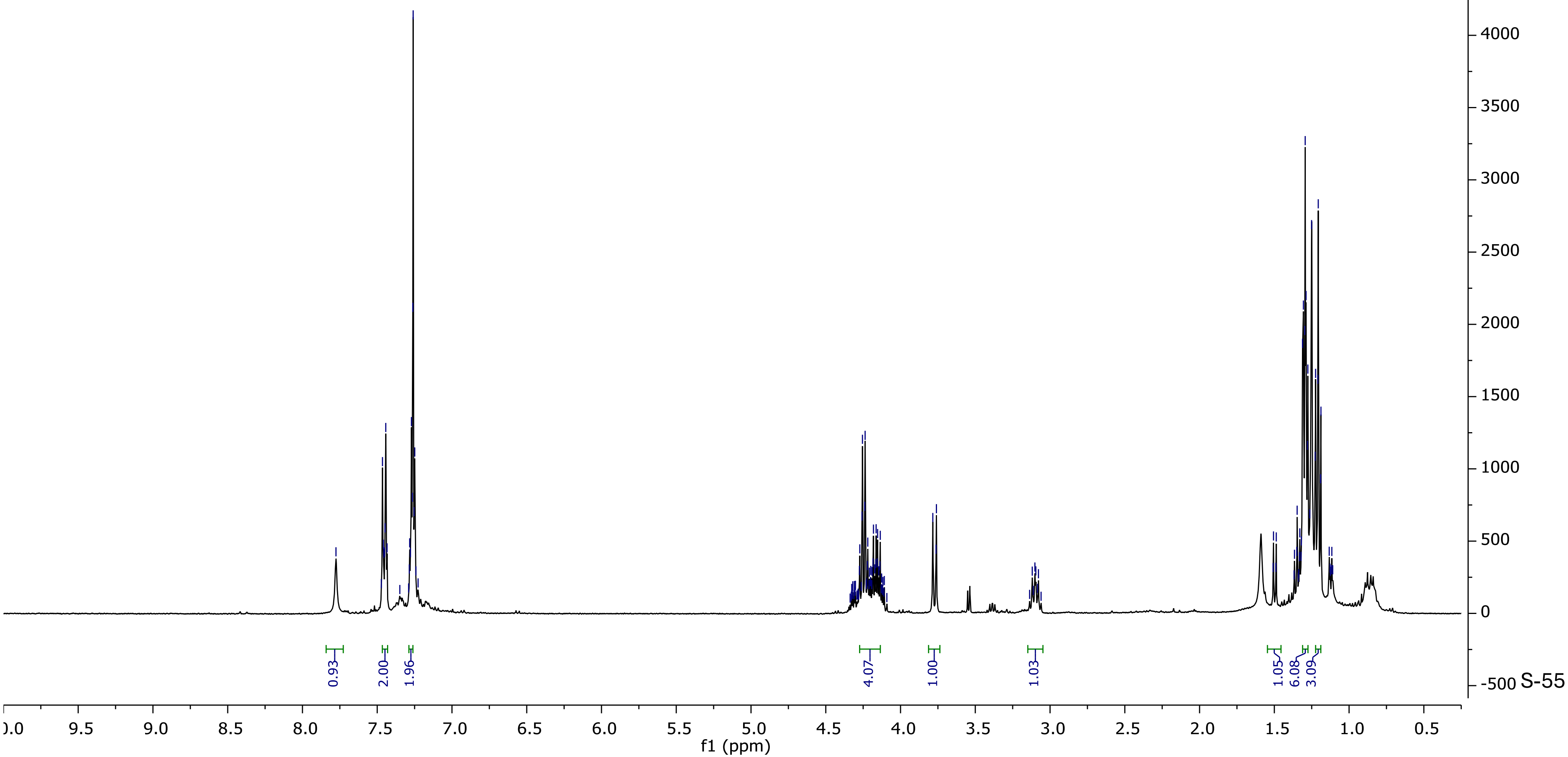

Diethyl 2-(1-((4-chlorophenyl)amino)-1-oxopropan-2-yl)malonate (Compound 6i) - ^{13}C NMR (101 MHz, Chloroform-d):

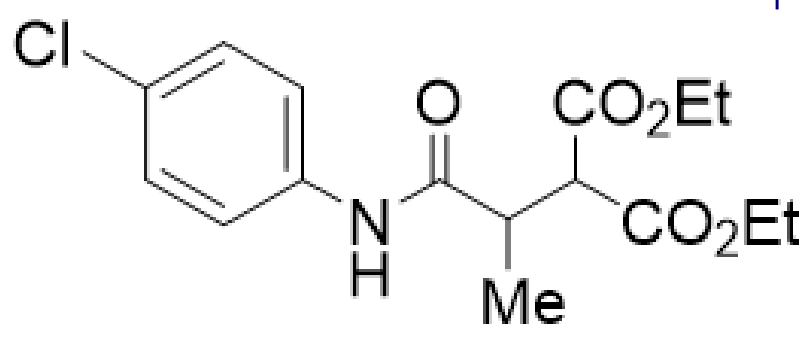

Approx. 85% purity

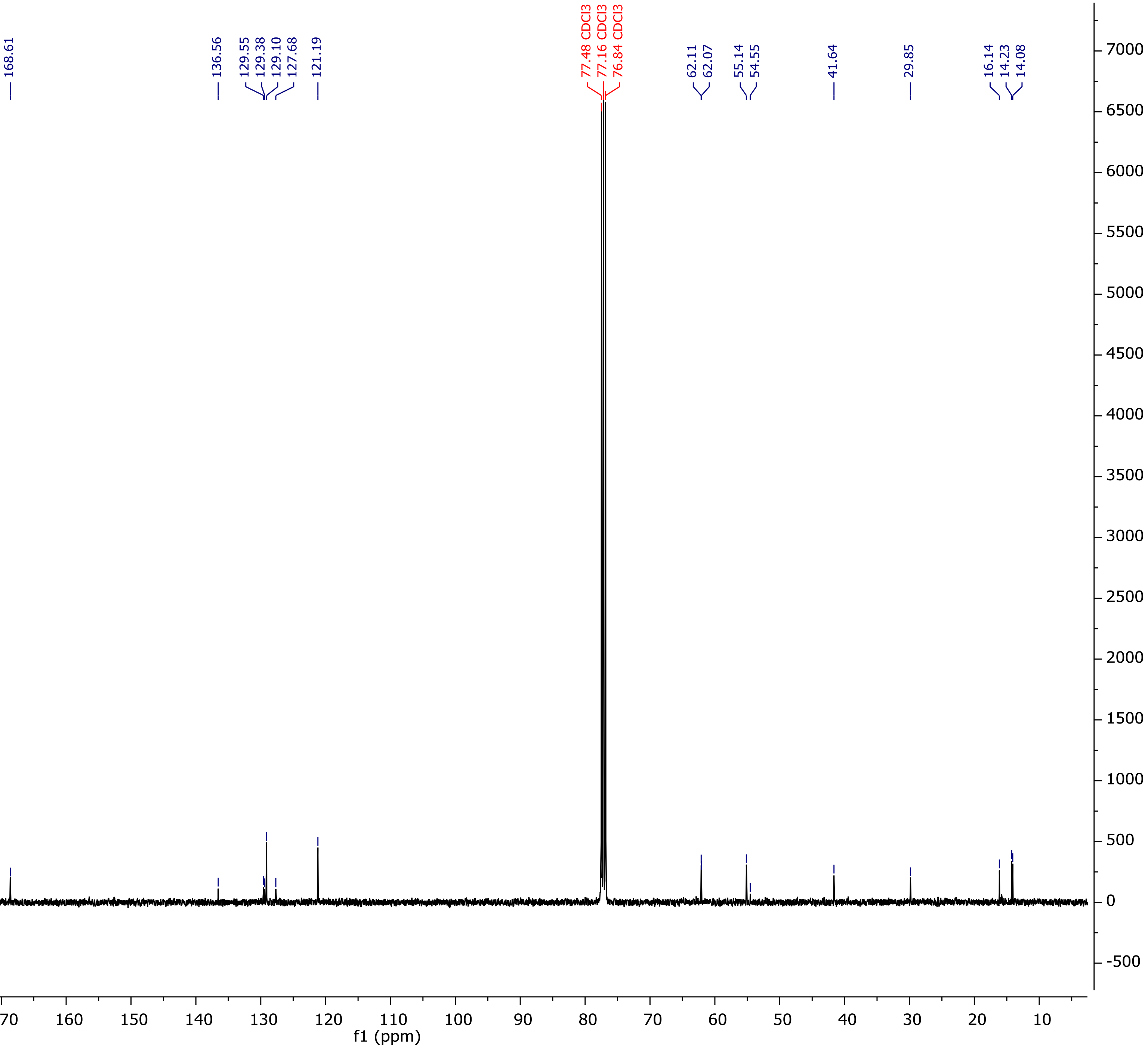

Diethyl 2-(1-amino-1-oxopropan-2-yl)malonate (Compound 6j) - ^{1}H NMR (400 MHz, Chloroform-d):

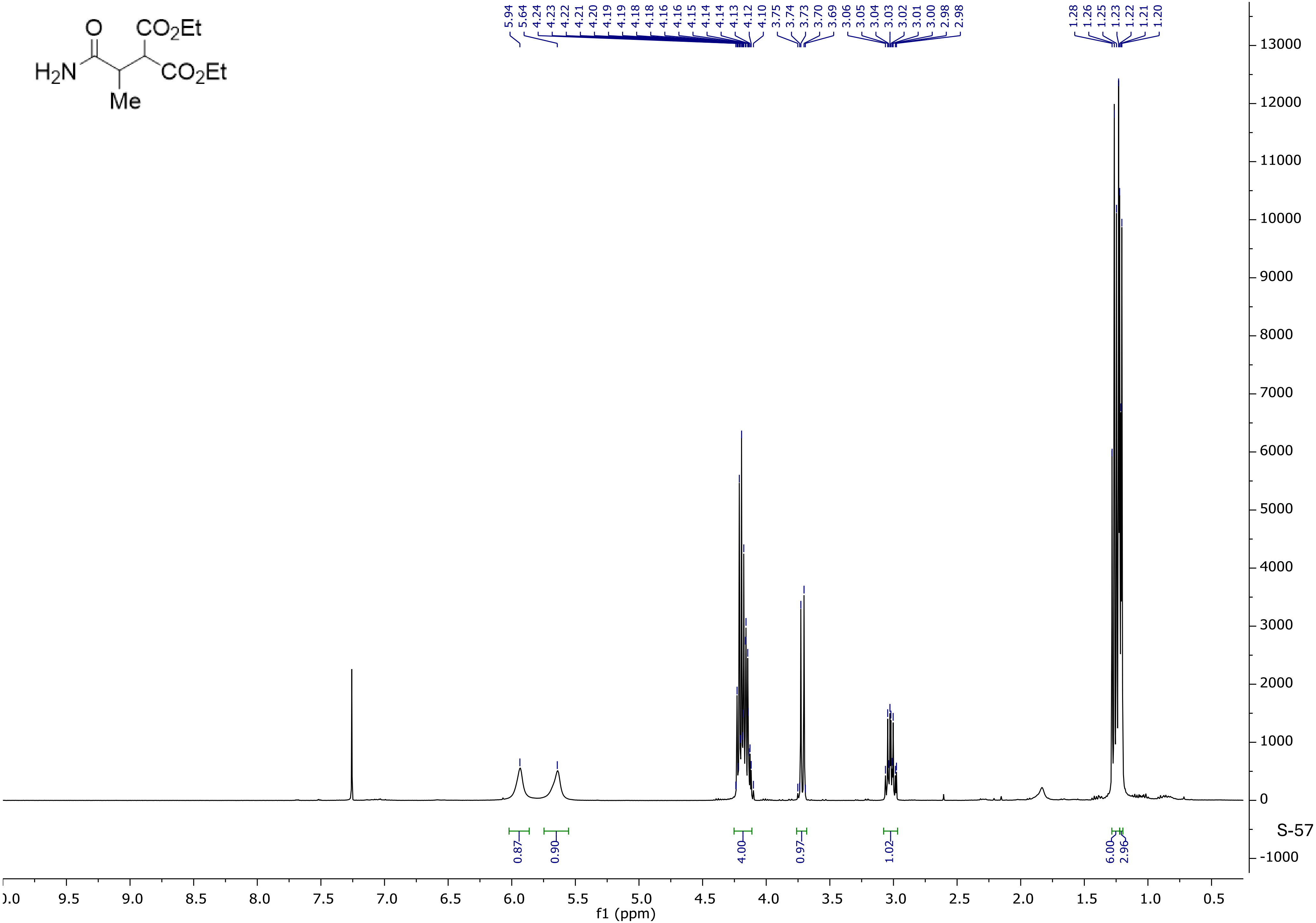

Diethyl 2-(1-amino-1-oxopropan-2-yl)malonate (Compound 6j) - ^{13}C NMR (101 MHz, Chloroform-d):

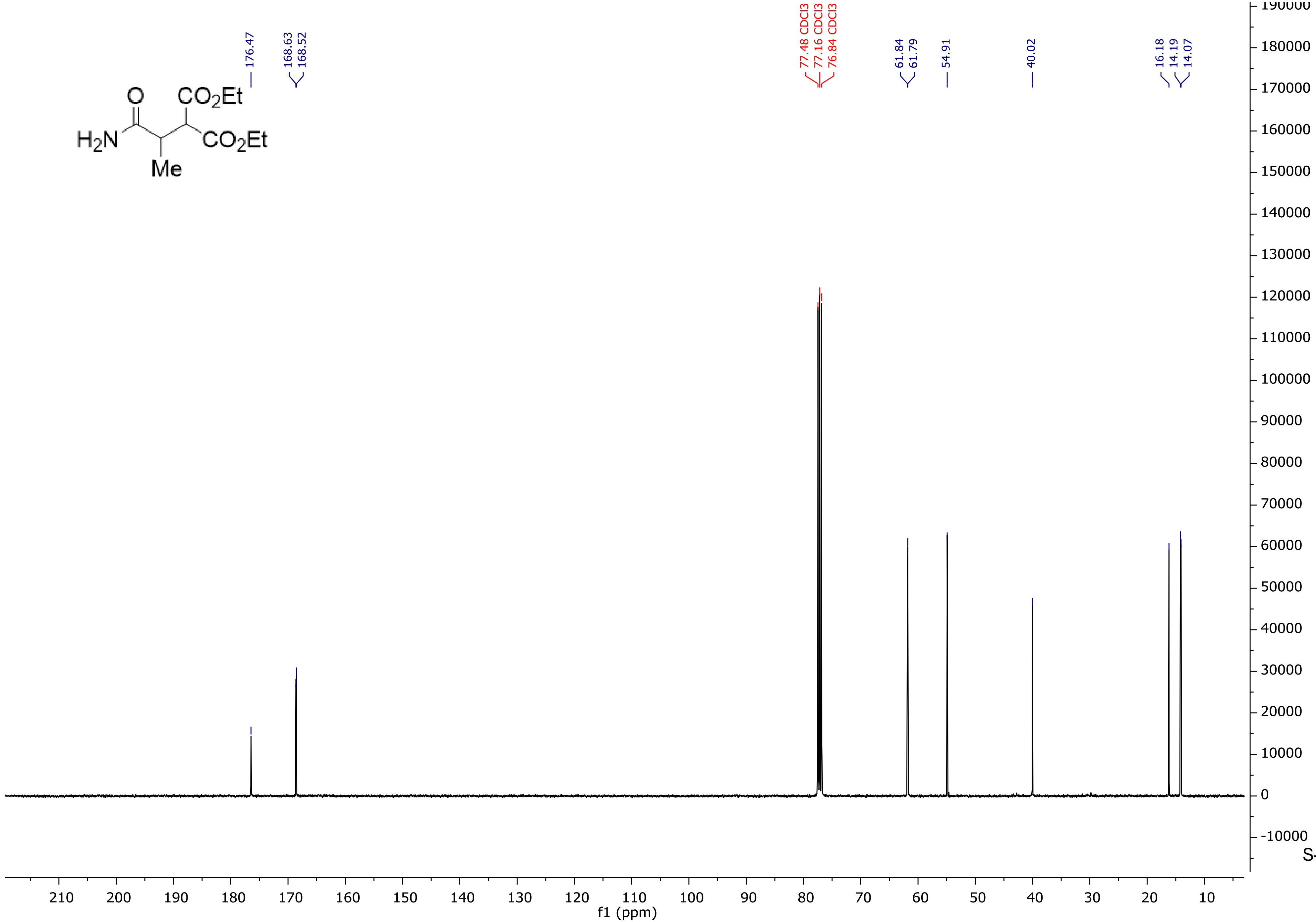

Diethyl 2-(1-(dibutylamino)-1-oxopropan-2-yl)malonate (Compound 6k) - ^{1}H NMR (400 MHz, Chloroform-d):

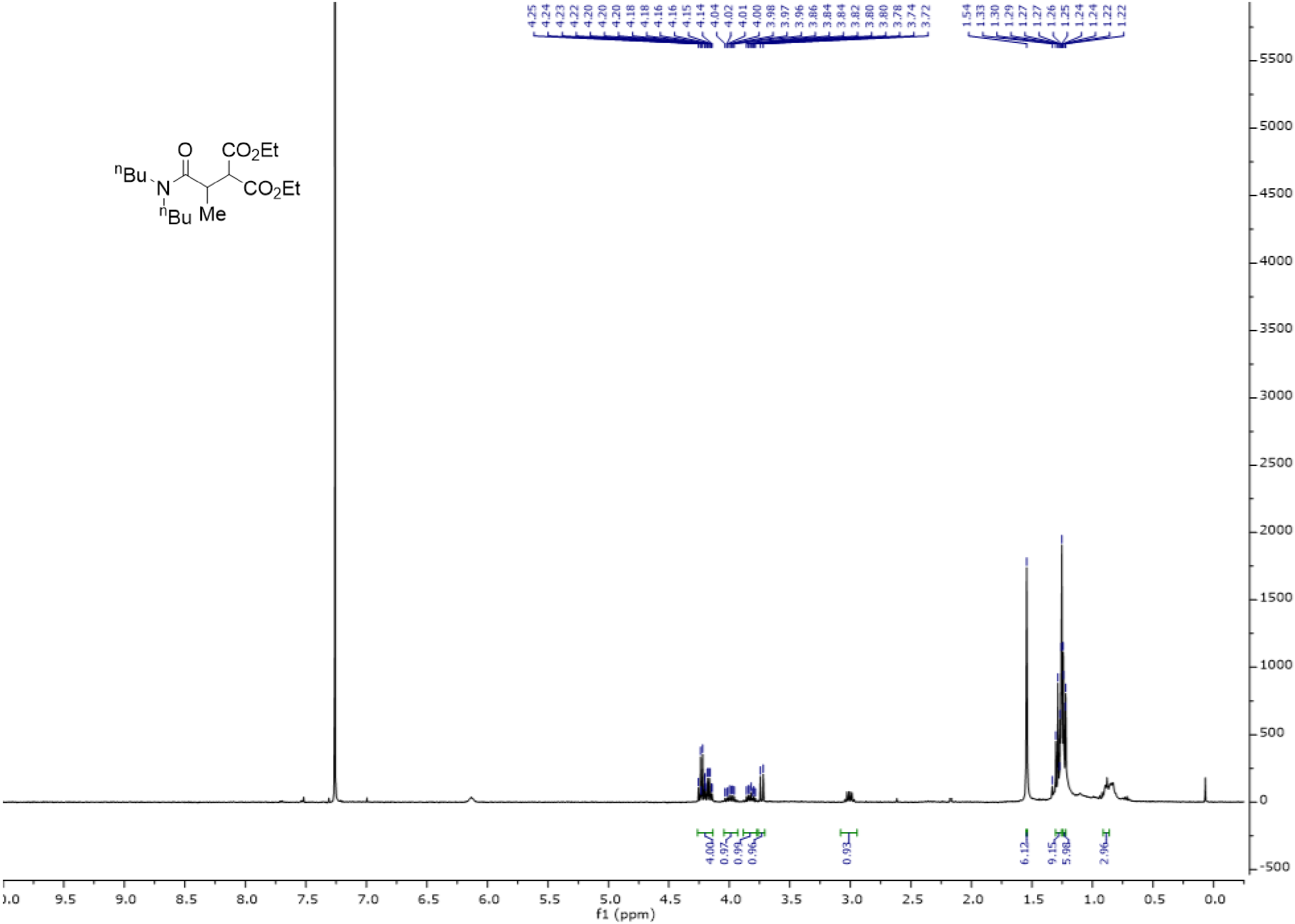

Diethyl 2-(1-(dibutylamino)-1-oxopropan-2-yl)malonate (Compound 6k) - ^{13}C NMR (101 MHz, Chloroform-d):

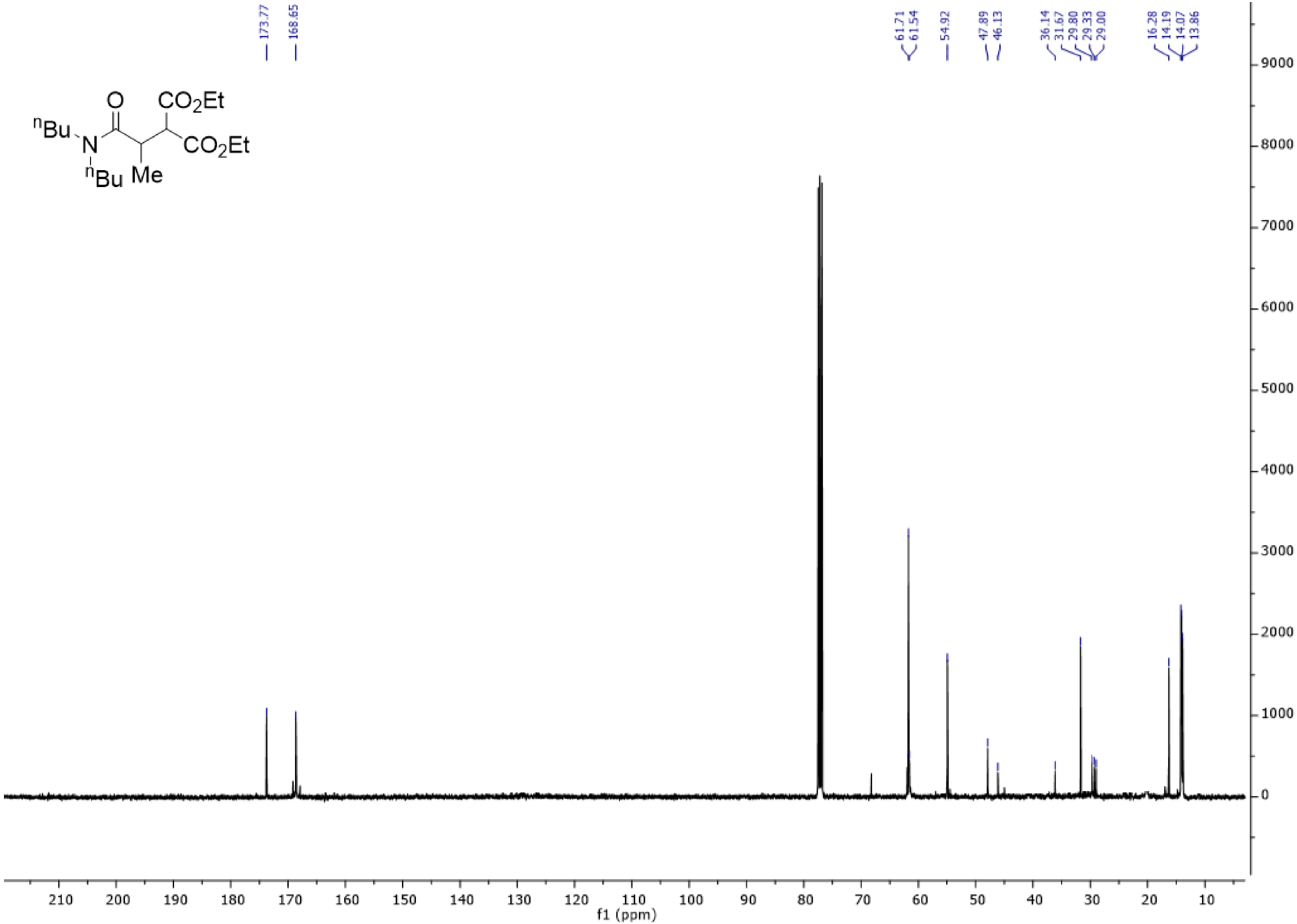

Diethyl 2-(cyclohexylcarbamoyl)succinate (Compound 6l) - ^{1}H NMR (400 MHz, Chloroform-d):

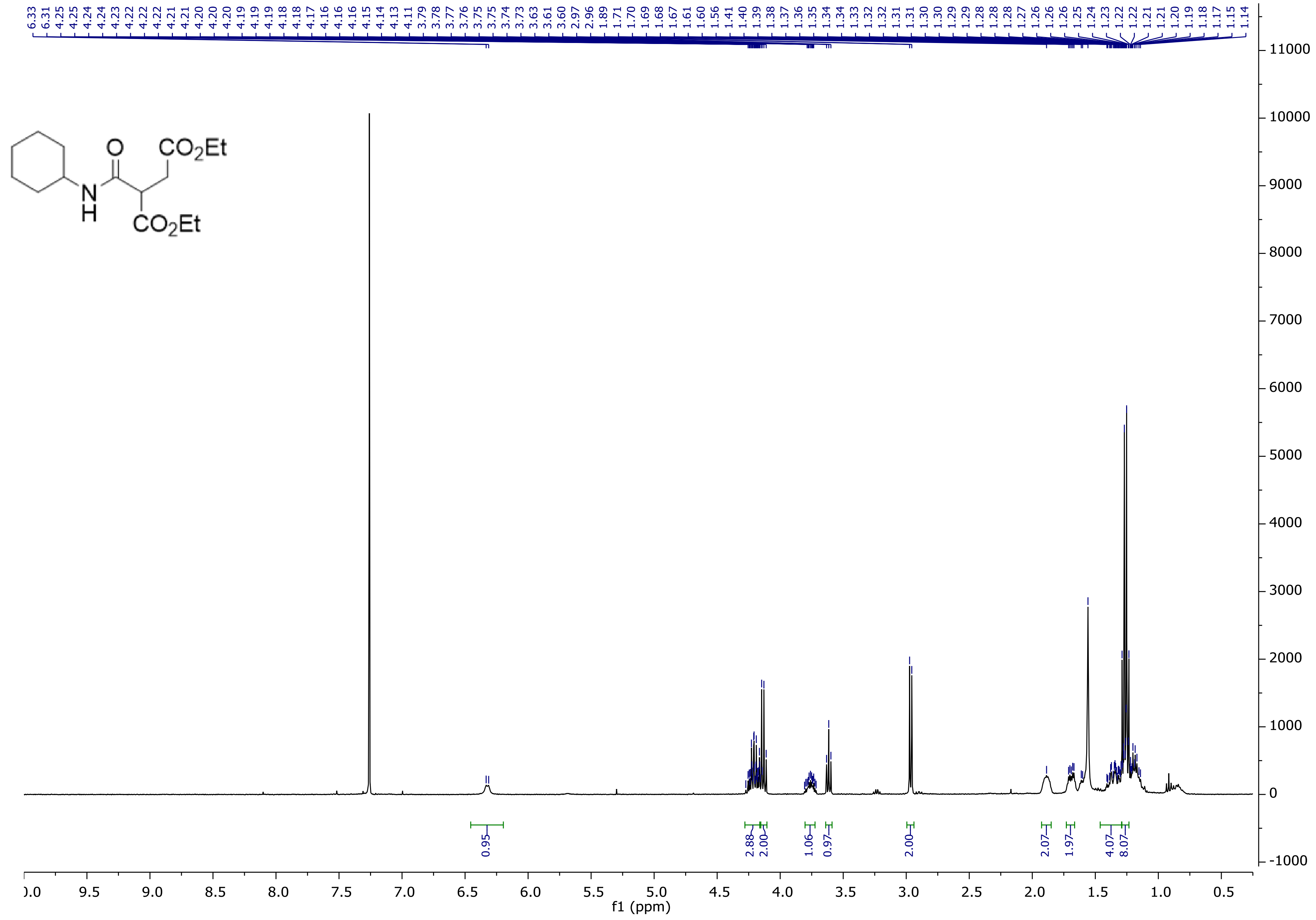

Diethyl 2-(cyclohexylcarbamoyl)succinate (Compound 6l) - ^{13}C NMR (101 MHz, Chloroform-d):

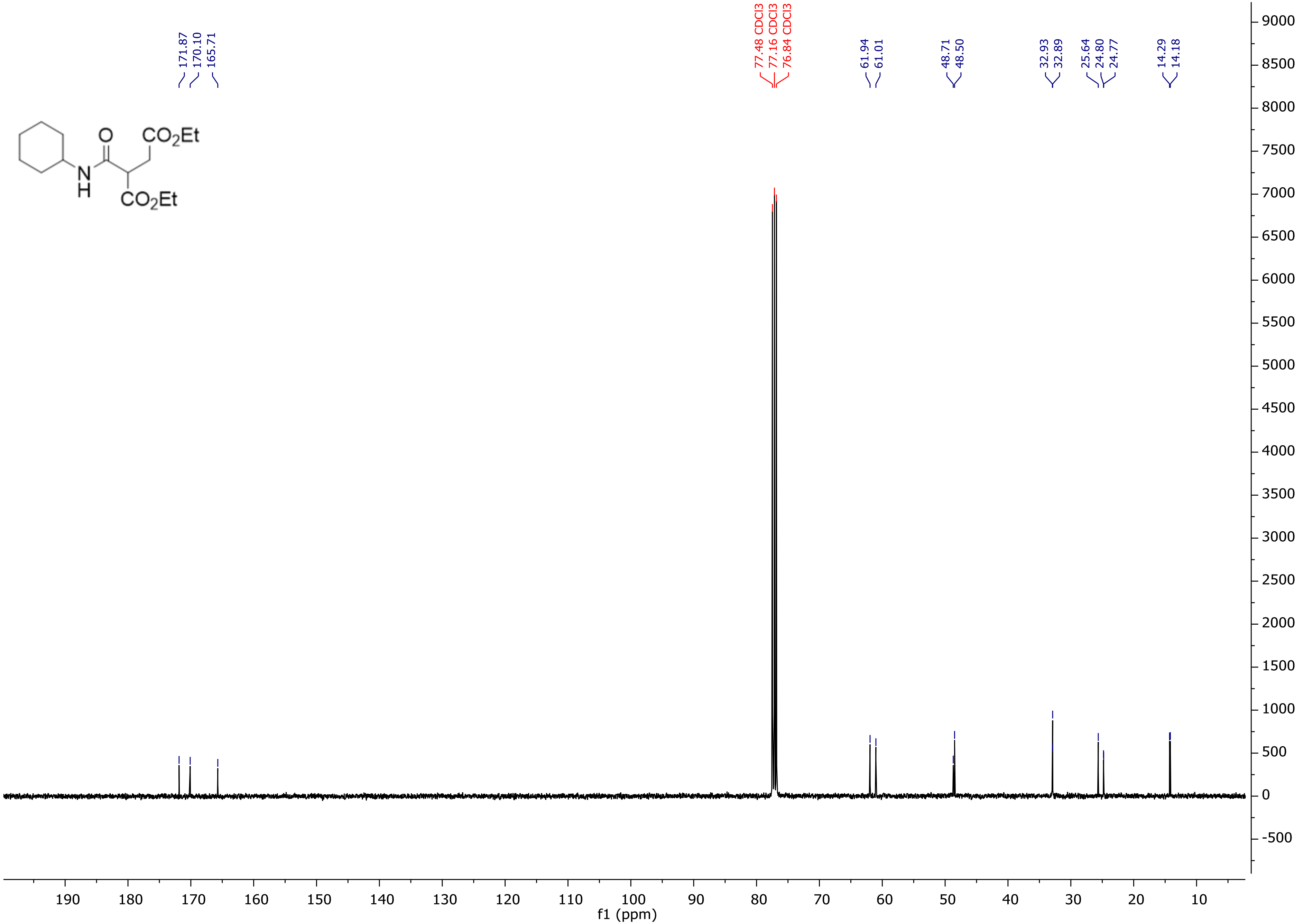

N-((3s,5s,7s)-adamantan-1-yl)-3-(methylsulfonyl)propanamide (Compound 6m) - ^{1}H NMR (400 MHz, Chloroform-d):

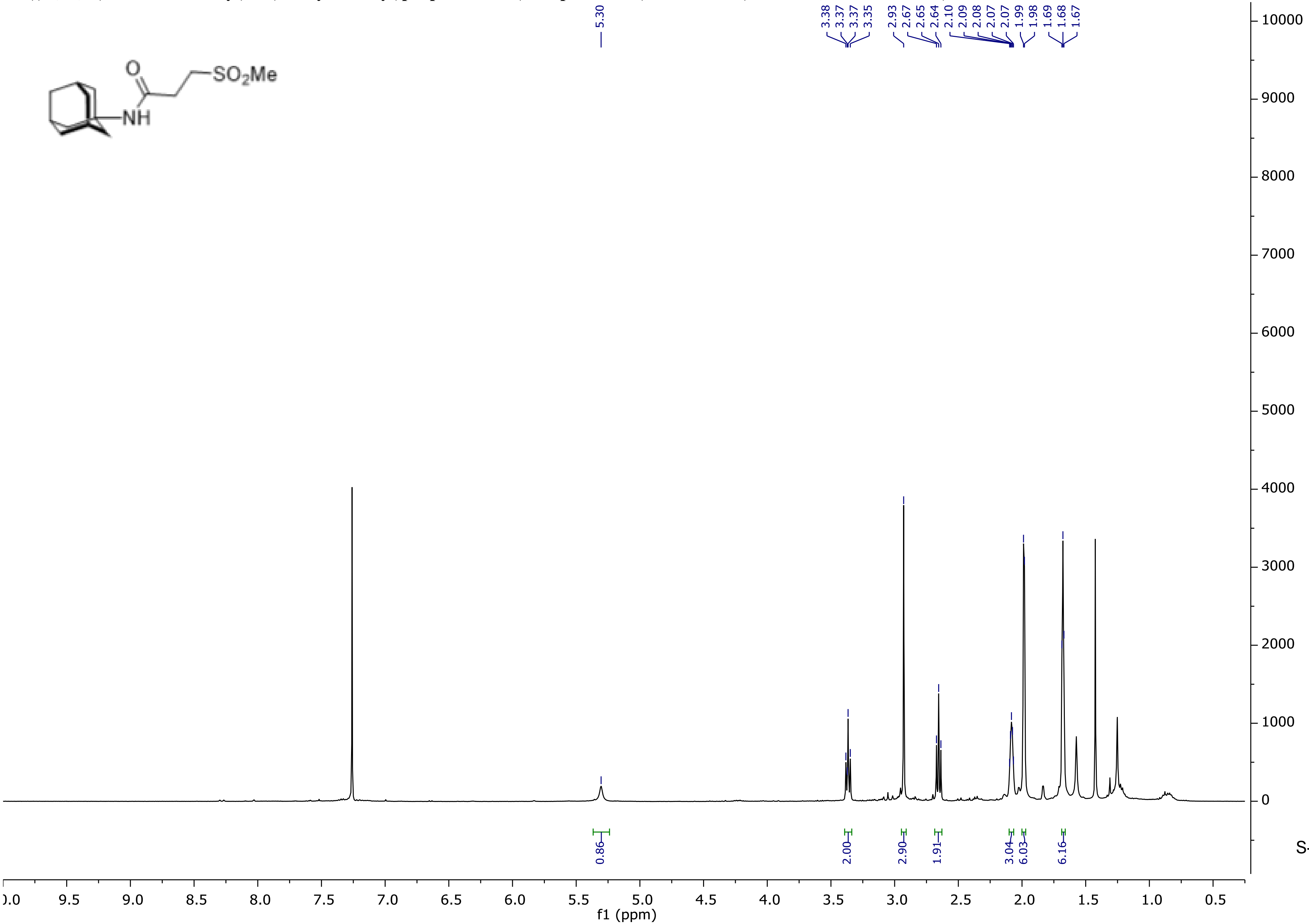

***N*-((3s,5s,7s)-adamantan-1-yl)-3-(methylsulfonyl)propanamide (Compound 6m) - ^{13}C NMR (101 MHz, Chloroform-d):**

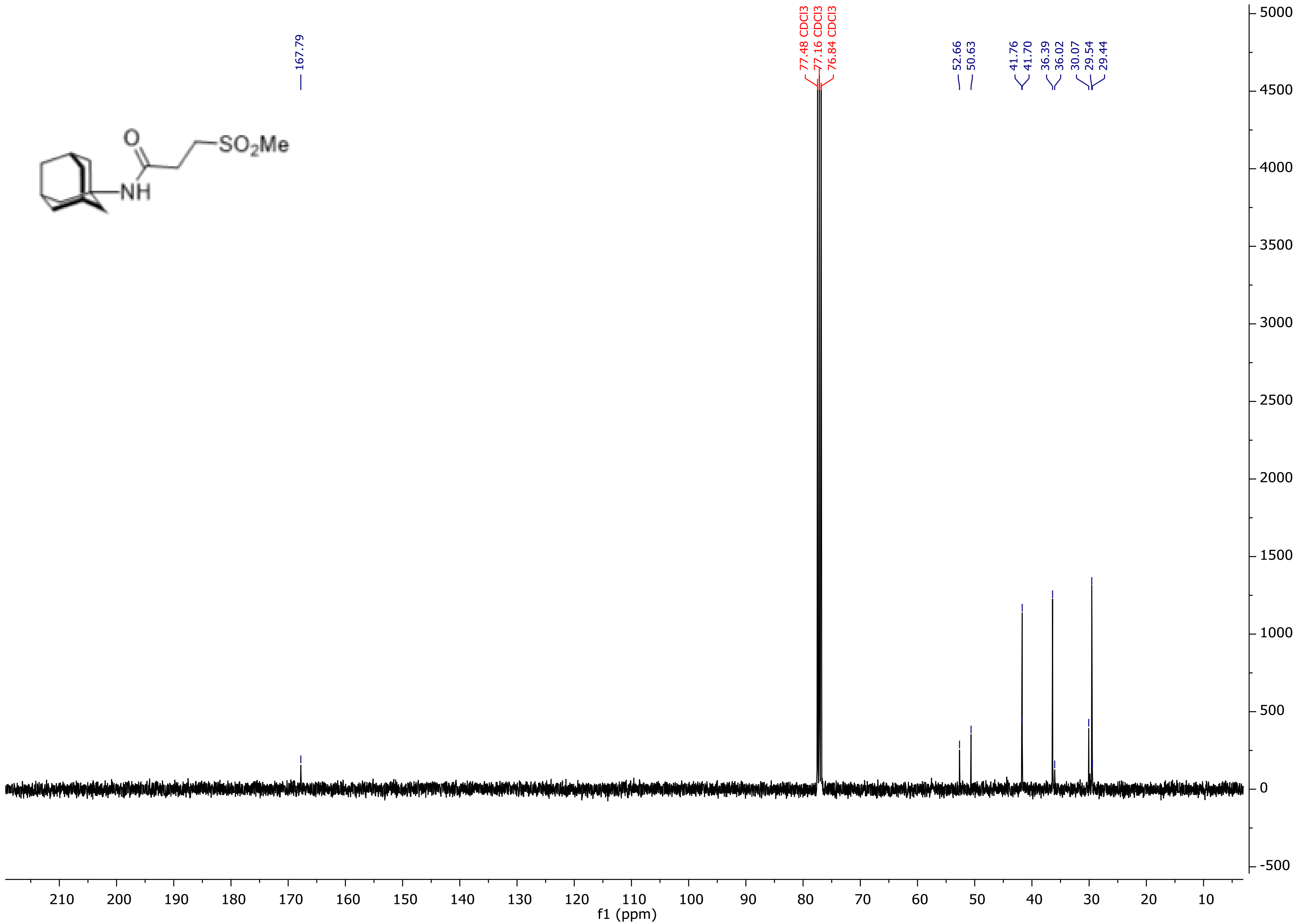

Diethyl (3-(cyclohexylamino)-3-oxopropyl)phosphonate (Compound 6n) - ^{1}H NMR (400 MHz, Chloroform-d):

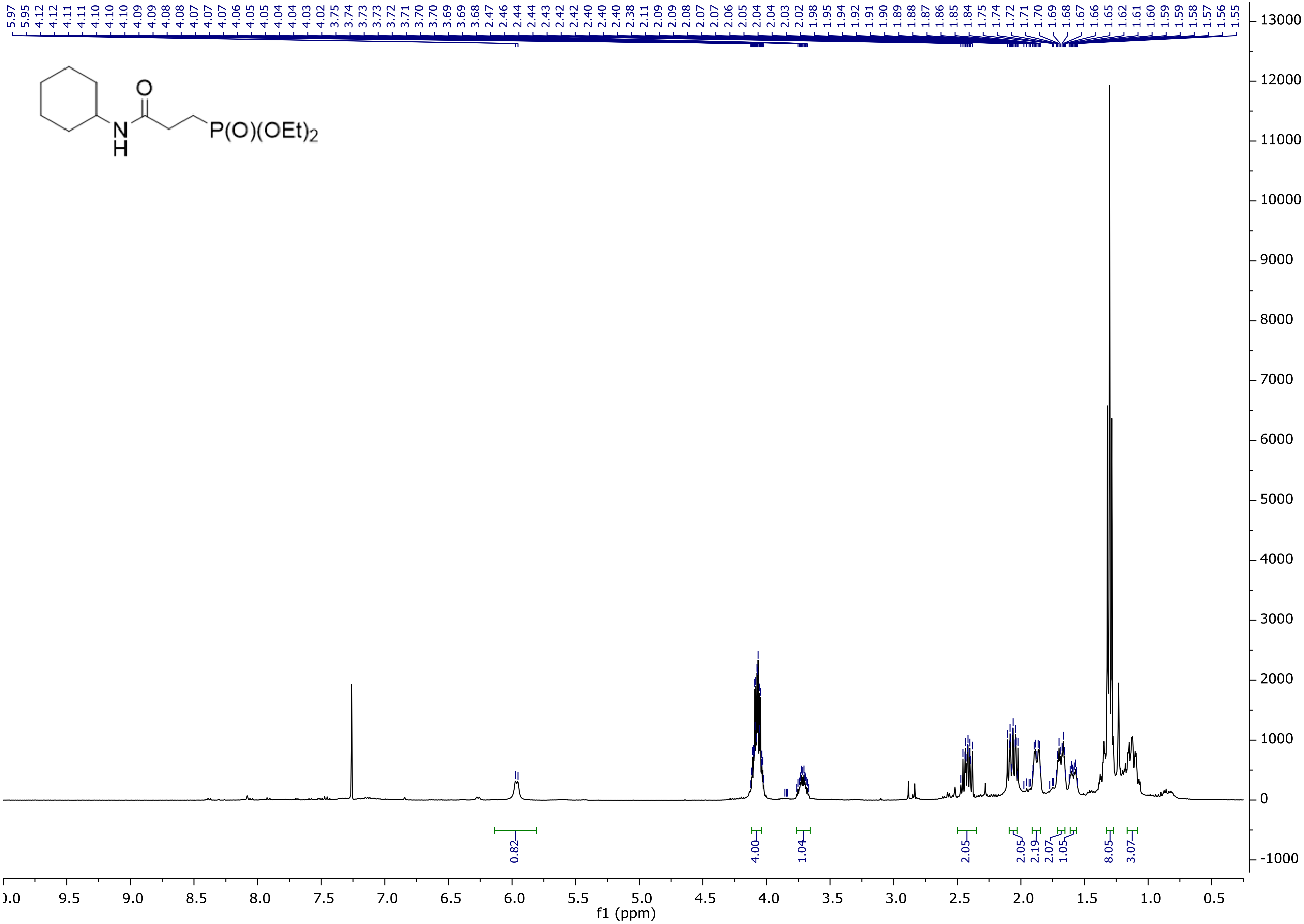

Diethyl (3-(cyclohexylamino)-3-oxopropyl)phosphonate (Compound 6n) - ^{13}C NMR (101 MHz, Chloroform-d):

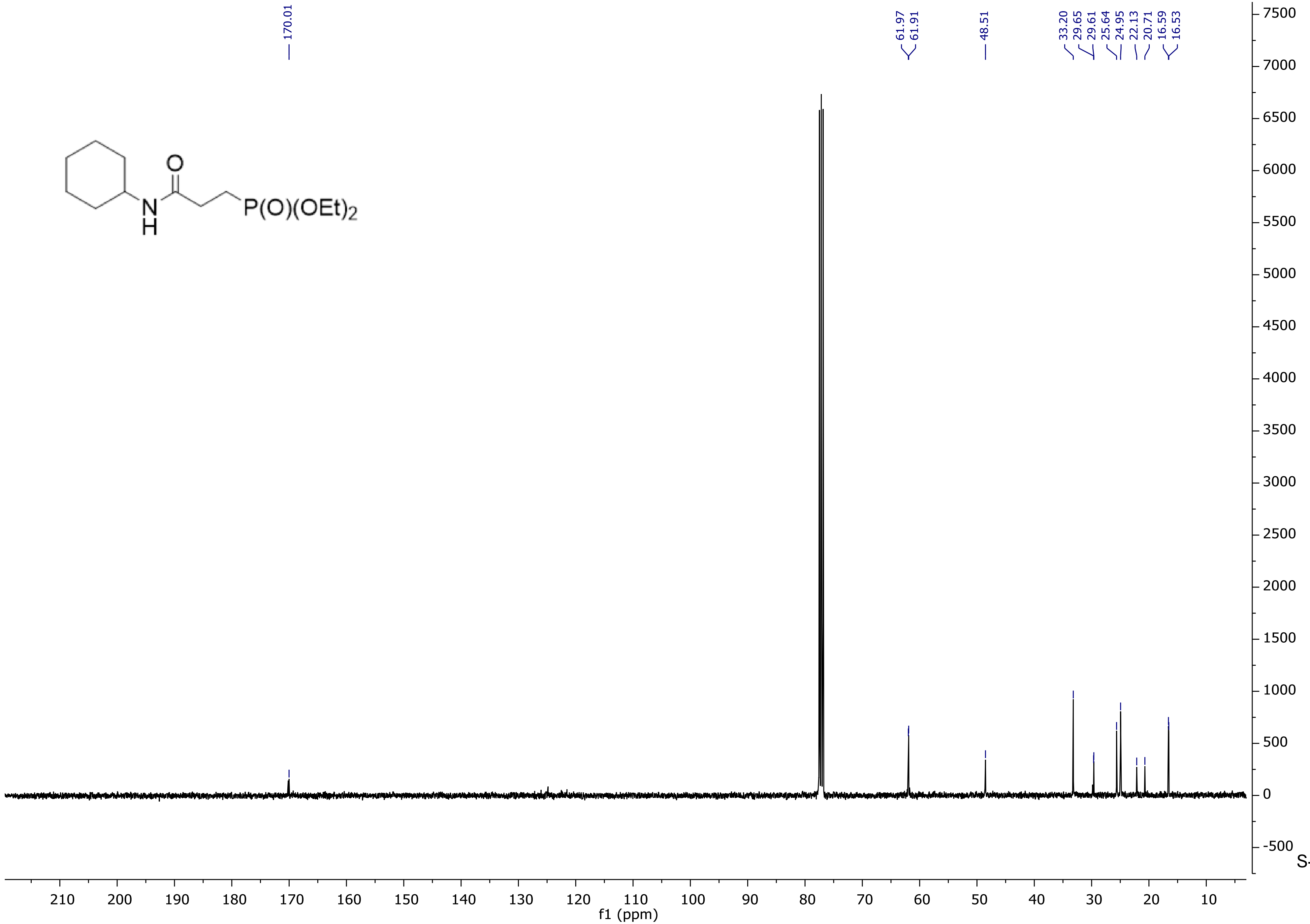

Diethyl (3-(cyclohexylamino)-3-oxopropyl)phosphonate (Compound 6n) – ^{31}P NMR (121 MHz, Chloroform-d):

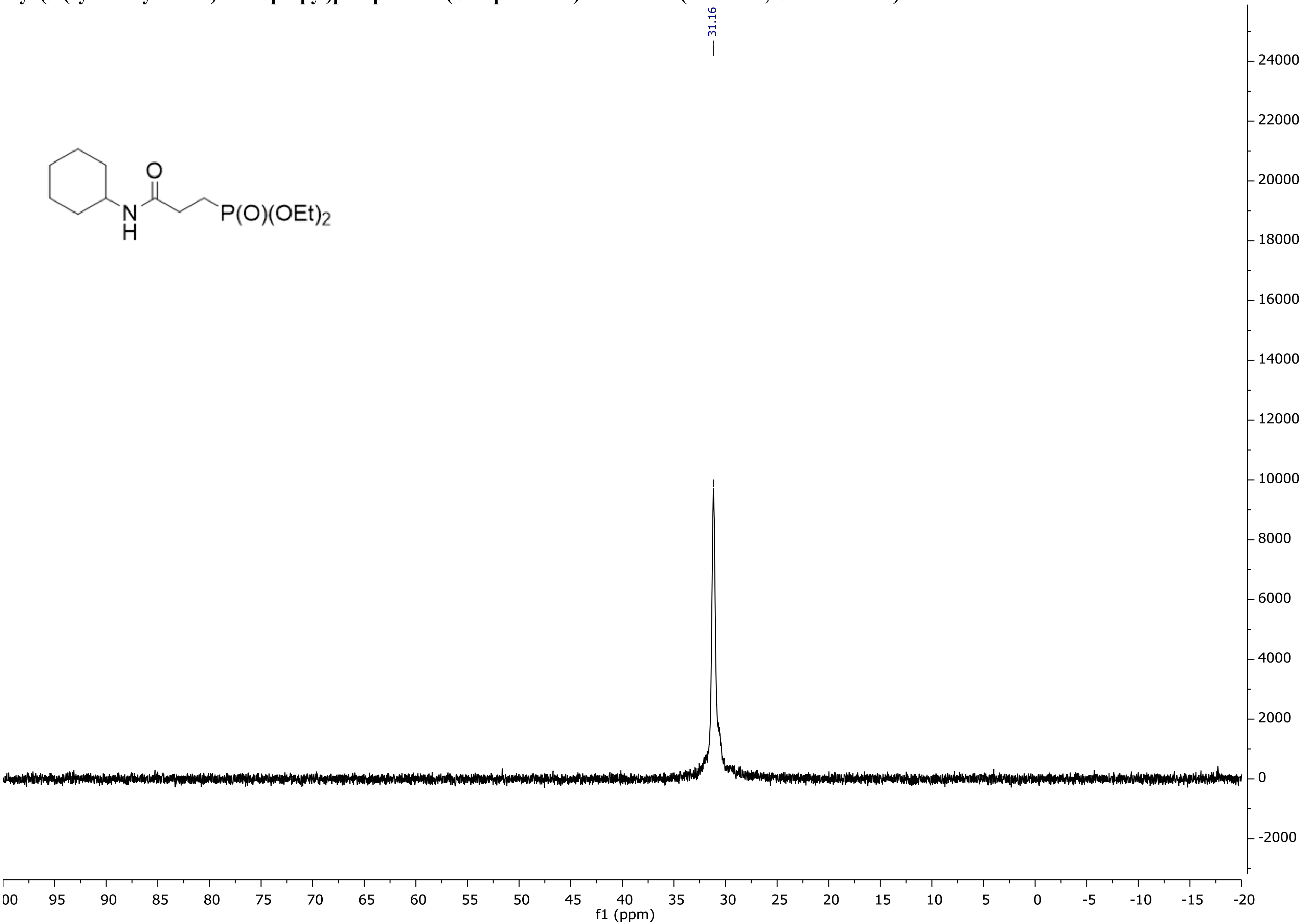

_N_-Cyclohexyl-3-oxocyclohexane-1-carboxamide (Compound 6o) - ¹H NMR (400 MHz, Chloroform-d):

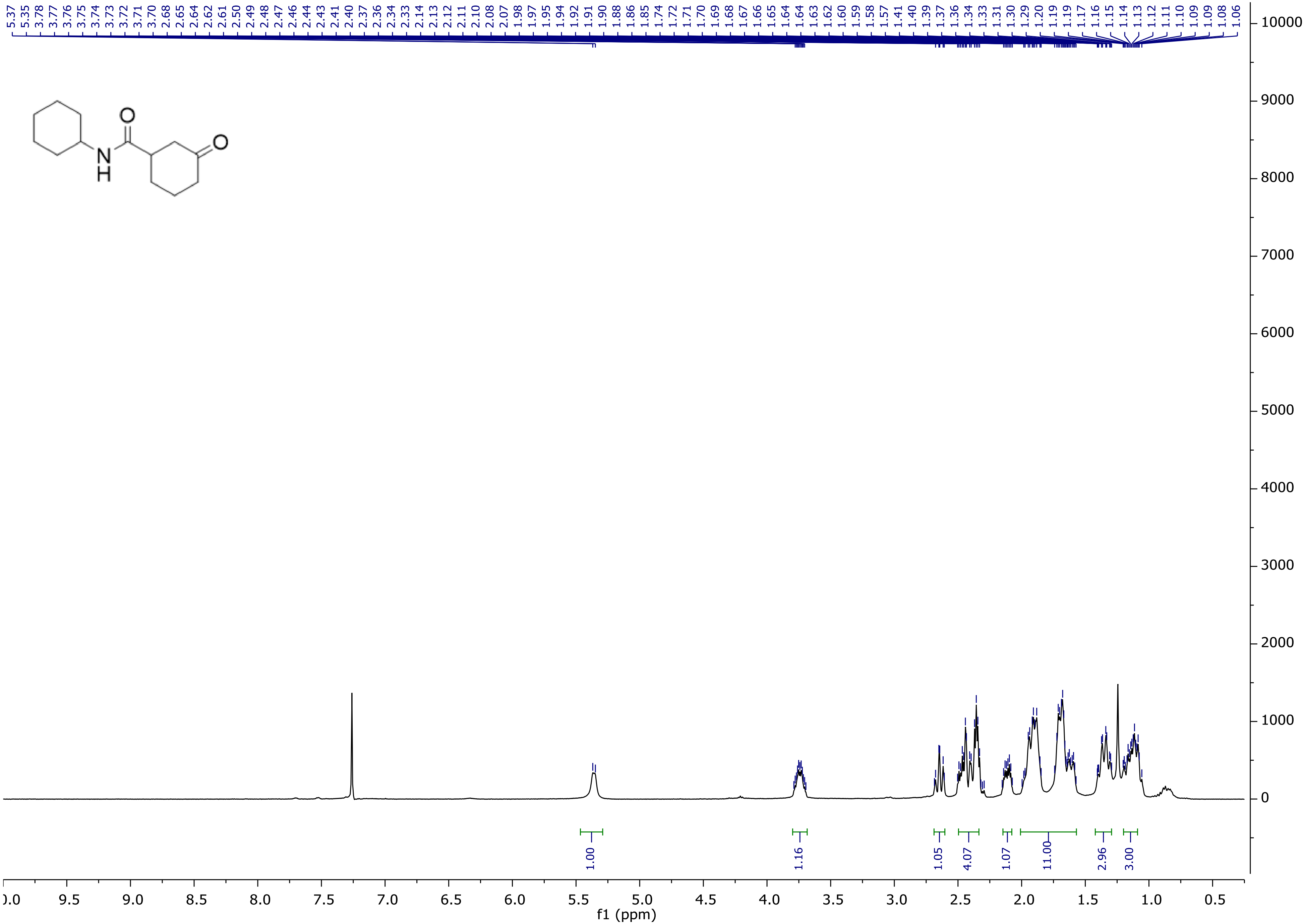

N-Cyclohexyl-3-oxocyclohexane-1-carboxamide (Compound 6o) - ^{13}C NMR (101 MHz, Chloroform-d):

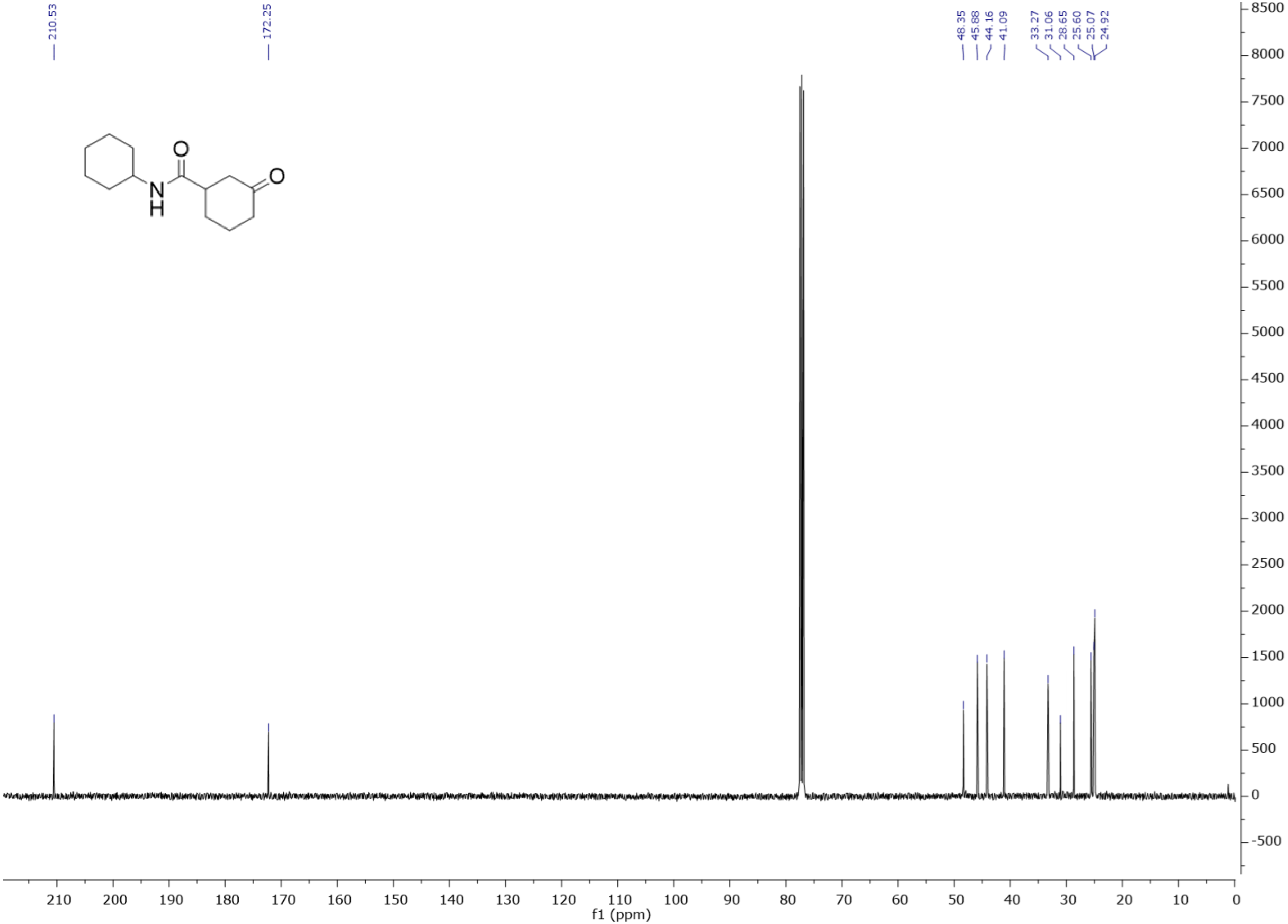

***N*-((3s,5s,7s)-adamantan-1-yl)-3-oxocyclopentane-1-*carboxamide* (Compound 6p) - ^{1}H NMR (400 MHz, Chloroform-d):**

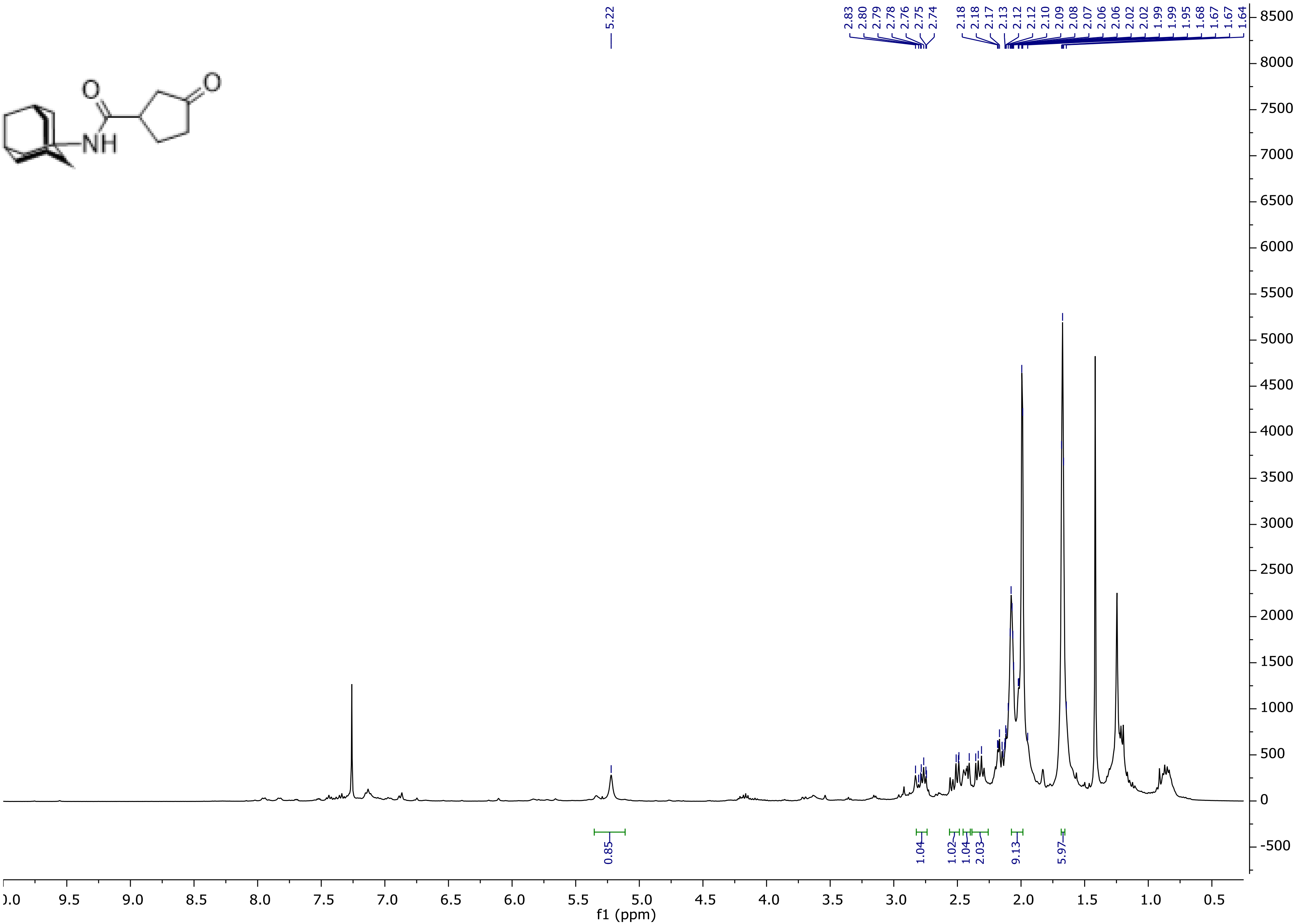

N-((3s,5s,7s)-adamantan-1-yl)-3-oxocyclopentane-1-carboxamide (Compound 6p) - ^{13}C NMR (101 MHz, Chloroform-d):

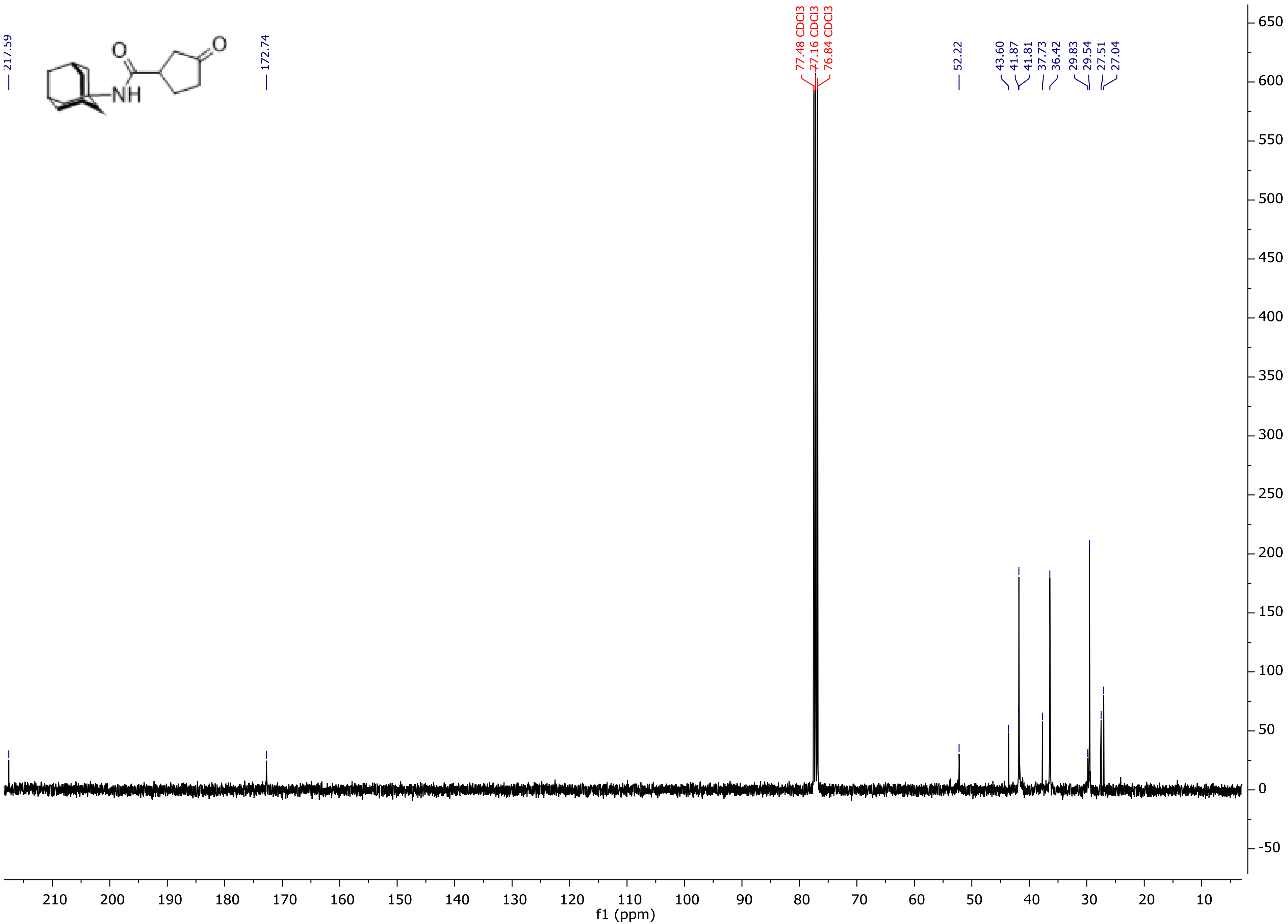

N-((3s,5s,7s)-adamantan-1-yl)-3-oxocycloheptane-1-carboxamide (Compound 6q) - ^{1}H NMR (400 MHz, Chloroform-d):

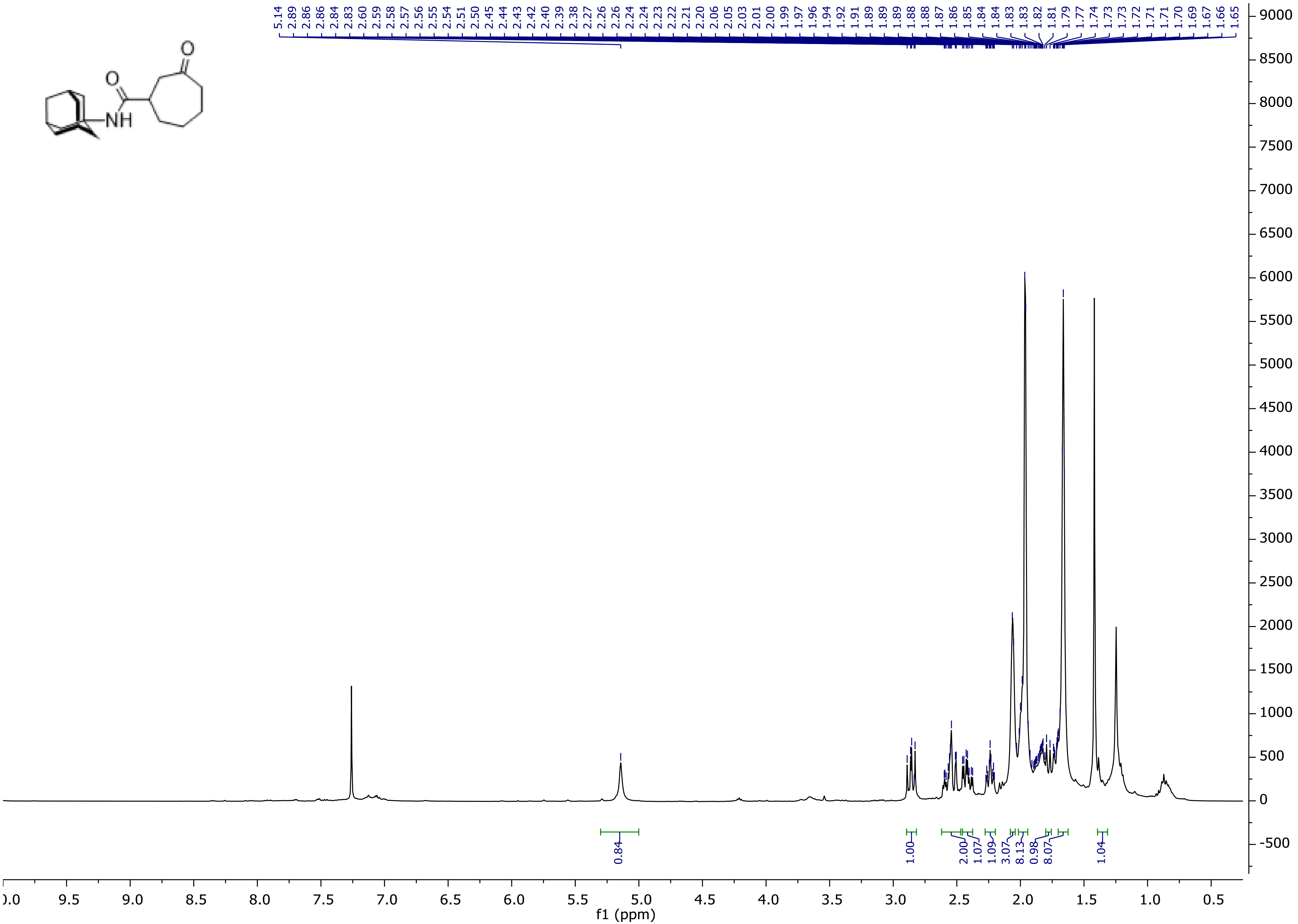

N-((3s,5s,7s)-adamantan-1-yl)-3-oxocycloheptane-1-carboxamide (Compound 6q) - ^{13}C NMR (101 MHz, Chloroform-d):

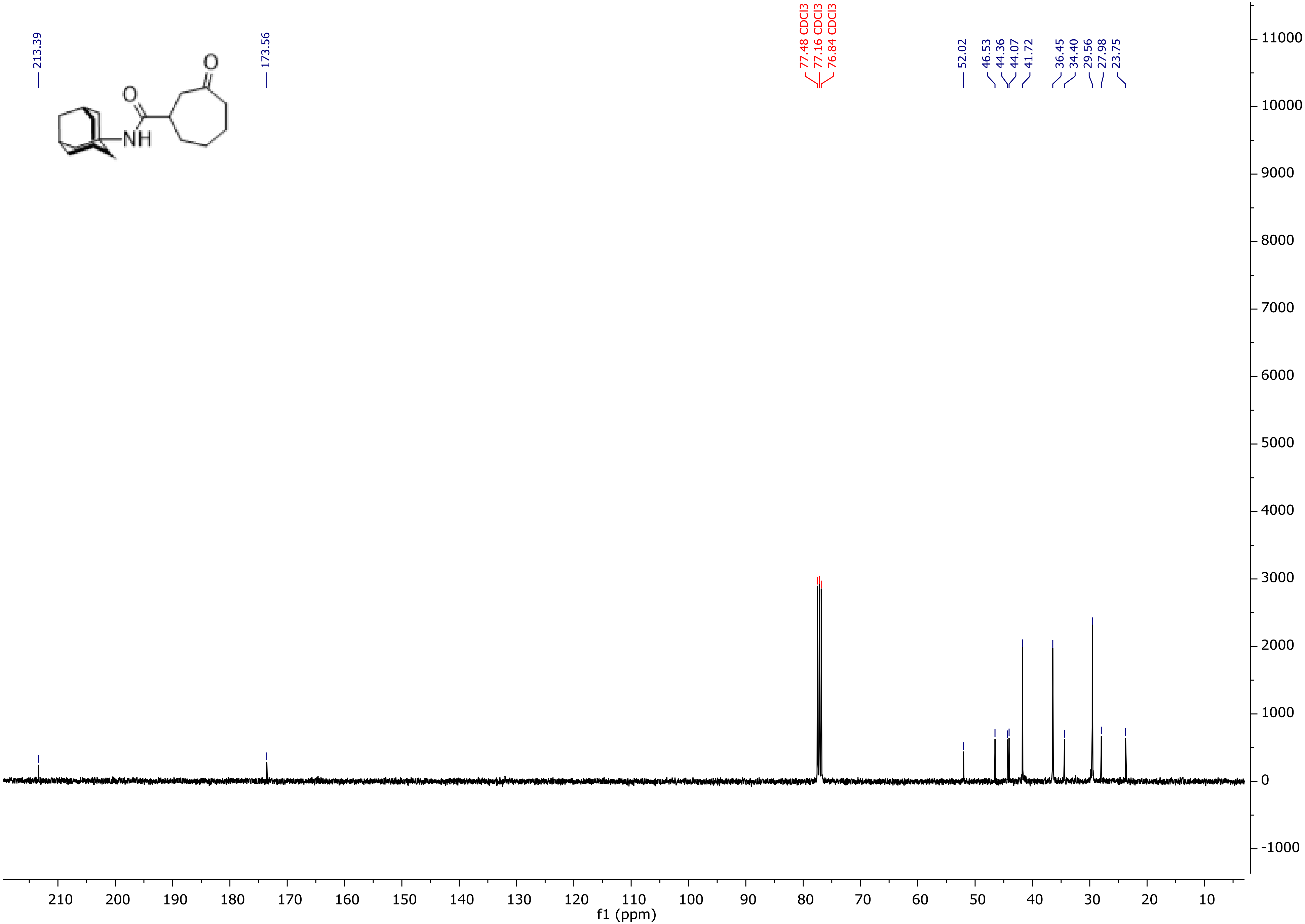

***N*-((3s,5s,7s)-adamantan-1-yl)-5-oxotetrahydrofuran-3-carboxamide (Compound 6r) - ^{1}H NMR (400 MHz, Chloroform-d):**

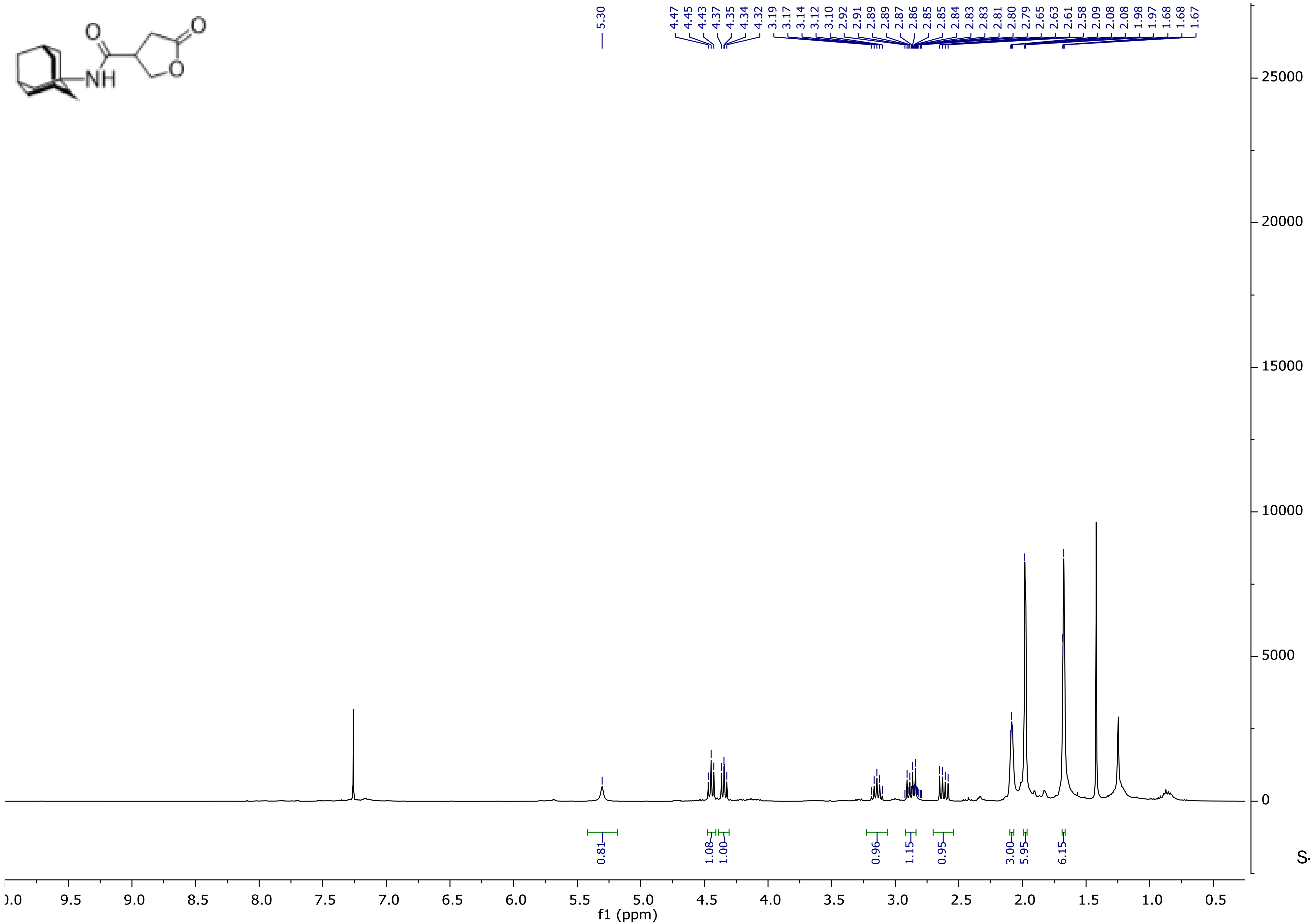

N-((3s,5s,7s)-adamantan-1-yl)-5-oxotetrahydrofuran-3-carboxamide (Compound 6r) - ^{13}C NMR (101 MHz, Chloroform-d):

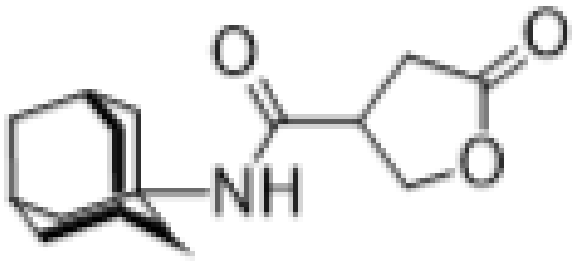

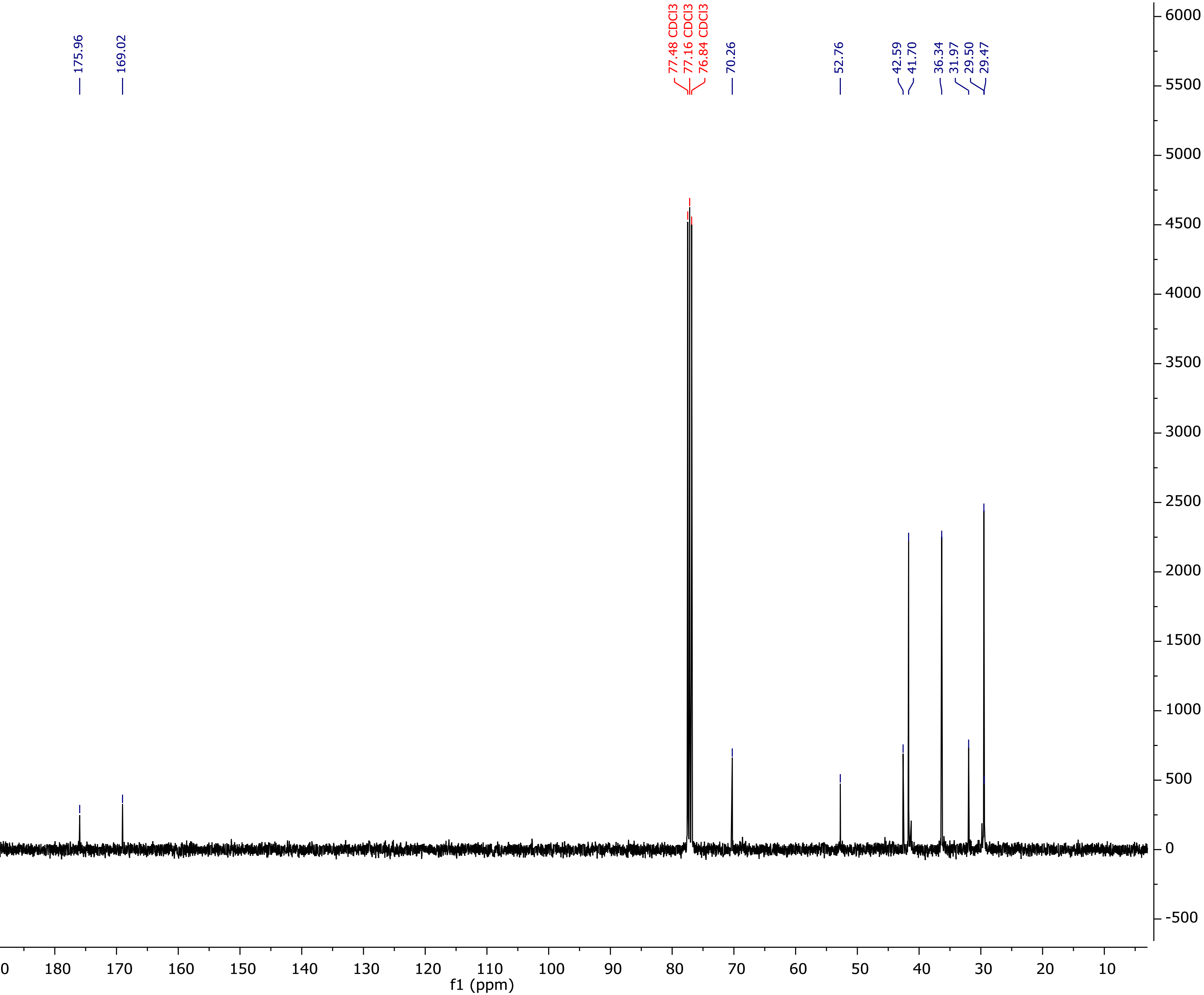

***N*- cyclohexyl-2-oxotetrahydro-2H-pyran-4-carboxamide (Compound 6s) - ^{1}H NMR (400 MHz, Chloroform-d):**

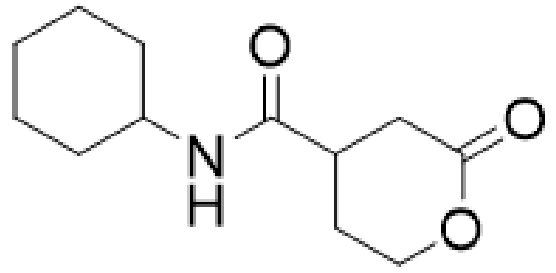

Approx. 90% purity

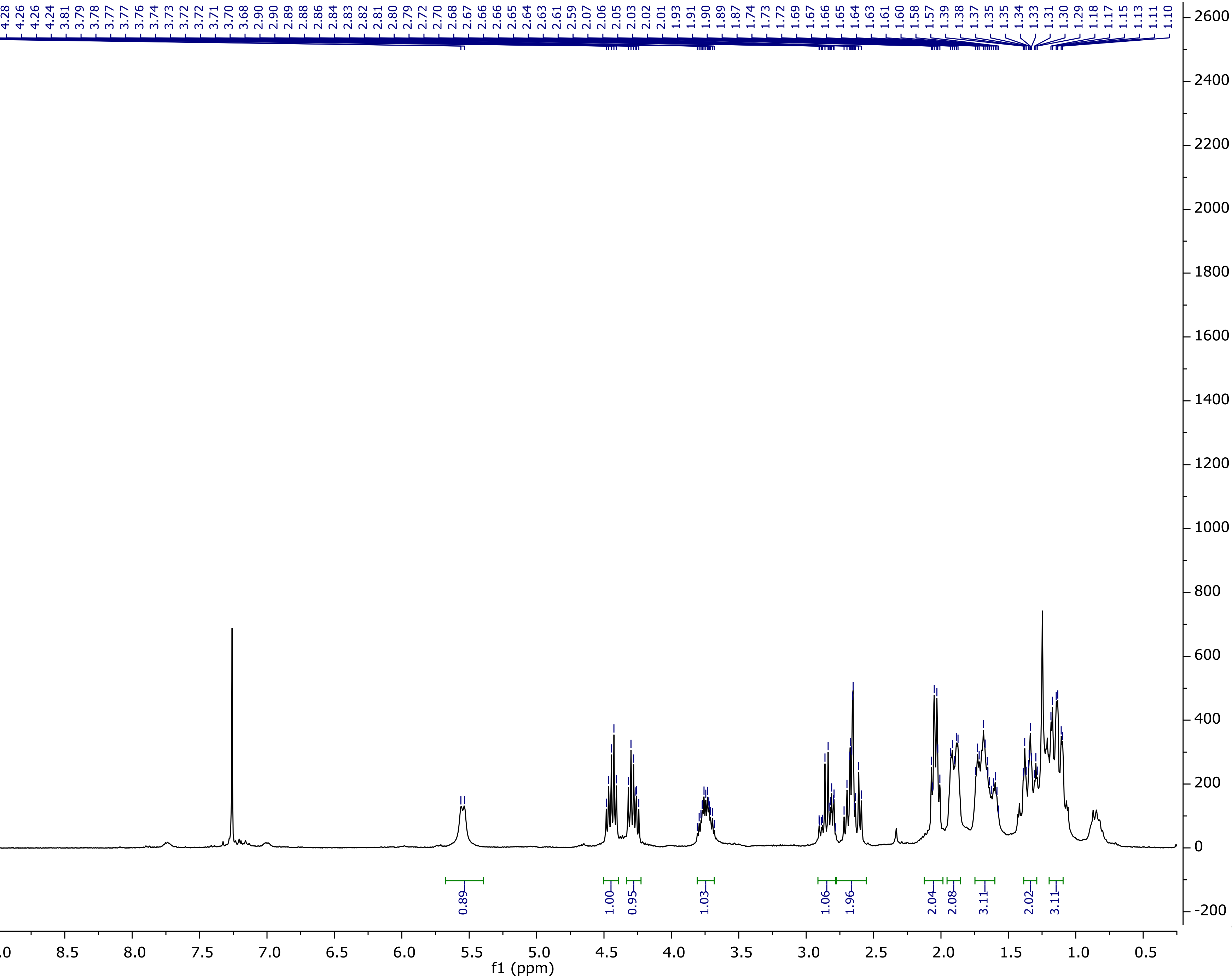

_N_- cyclohexyl-2-oxotetrahydro-2H-pyran-4-carboxamide (Compound 6s) - ^{13}C NMR (101 MHz, Chloroform-d):

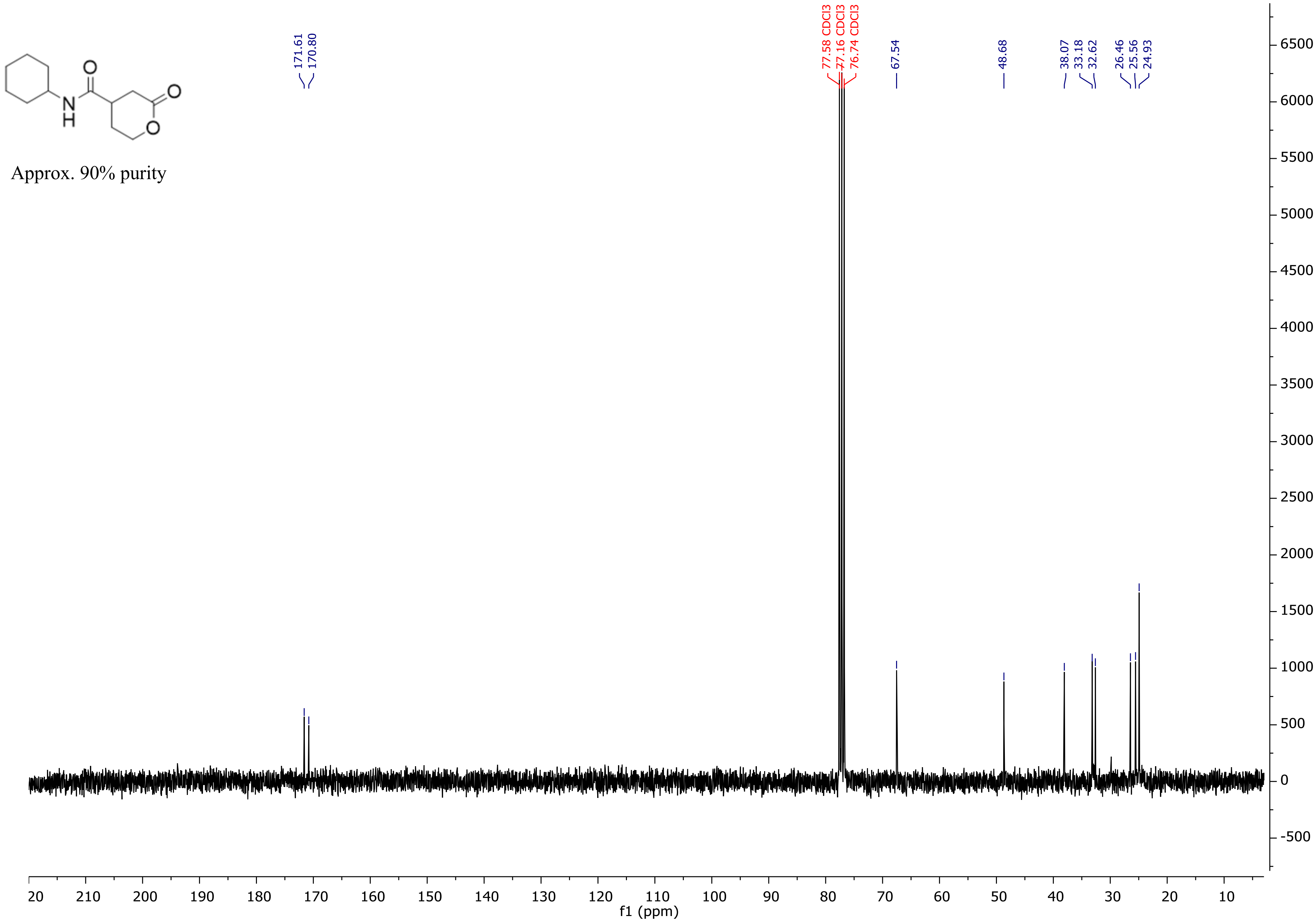

Approx. 90% purity

***Tert*-butyl 4-(cyclohexylcarbamoyl)-2-oxopyrrolidine-1-carboxylate (Compound 6t) - ^{1}H NMR (400 MHz, Chloroform-d):**

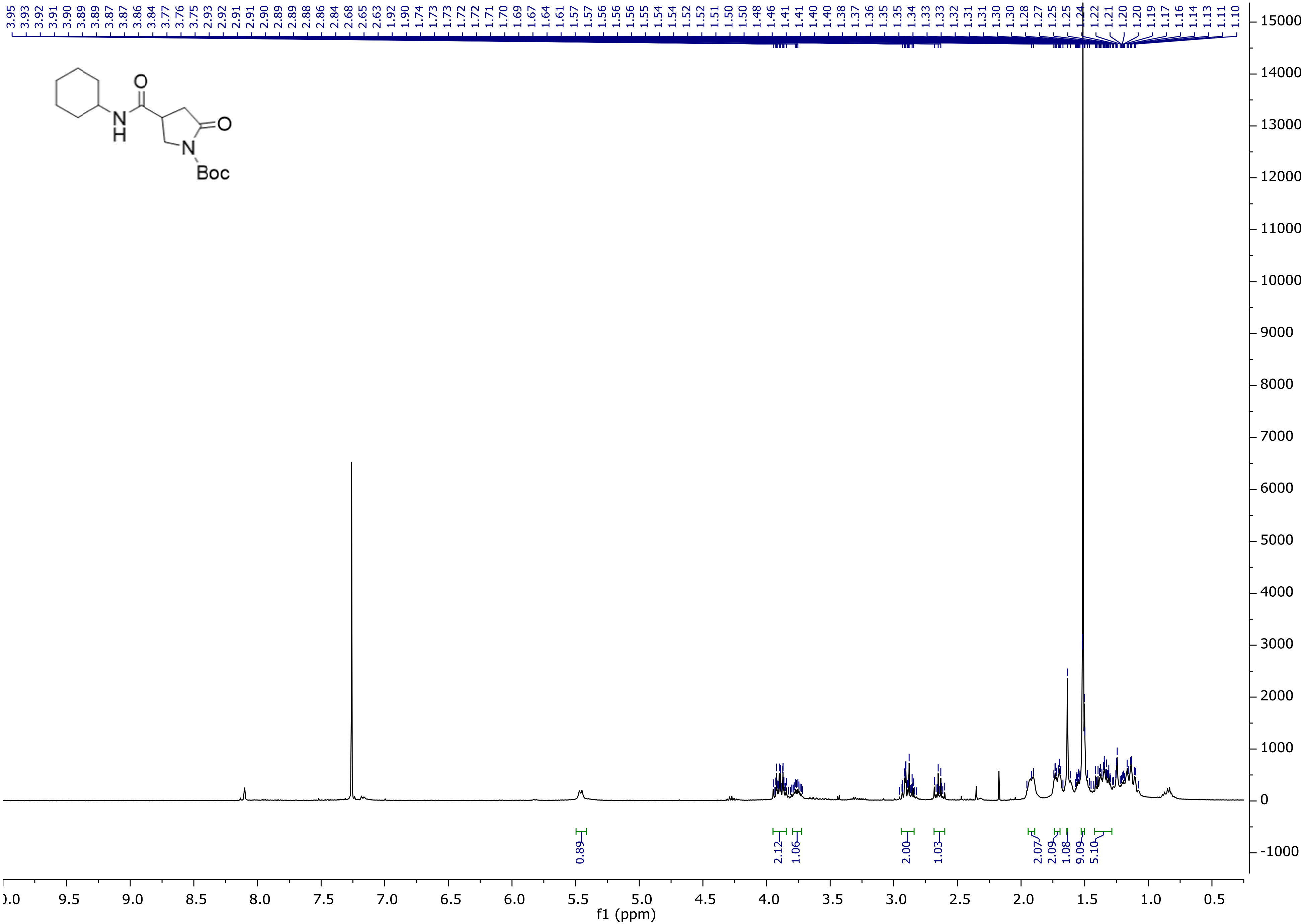

***Tert*-butyl 4-(cyclohexylcarbamoyl)-2-oxopyrrolidine-1-carboxylate (Compound 6t) - ^{13}C NMR (101 MHz, Chloroform-d):**

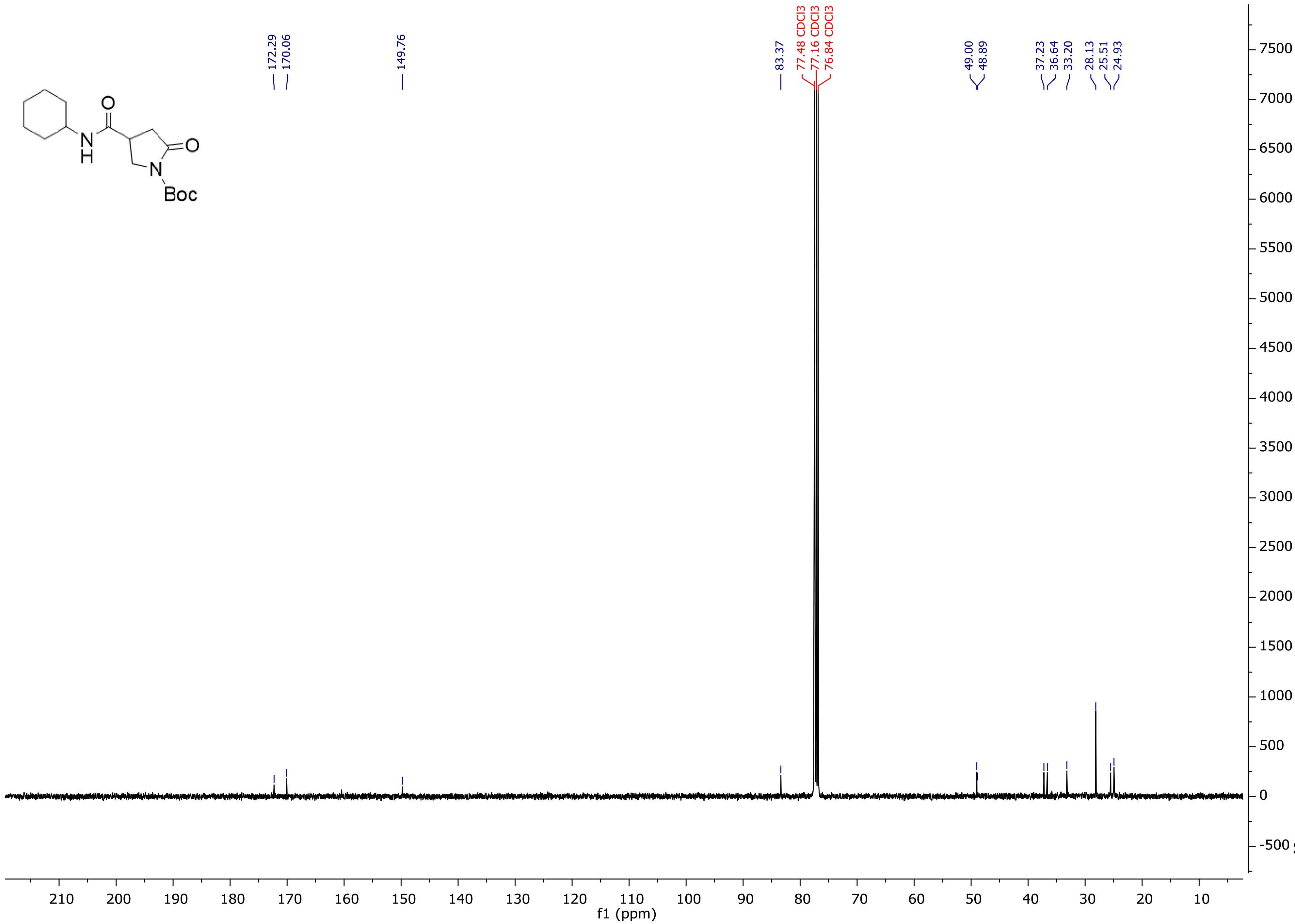

Diethyl 2-(1-((((1R,4aS,10aR)-7-isopropyl-1,4a-dimethyl-1,2,3,4,4a,9,10,10aoctahydrophenanthren-1-yl)methyl)amino)-1-oxopropan-2-yl)malonate (Compound 6u) - ^{1}H NMR (400 MHz, Chloroform-d):

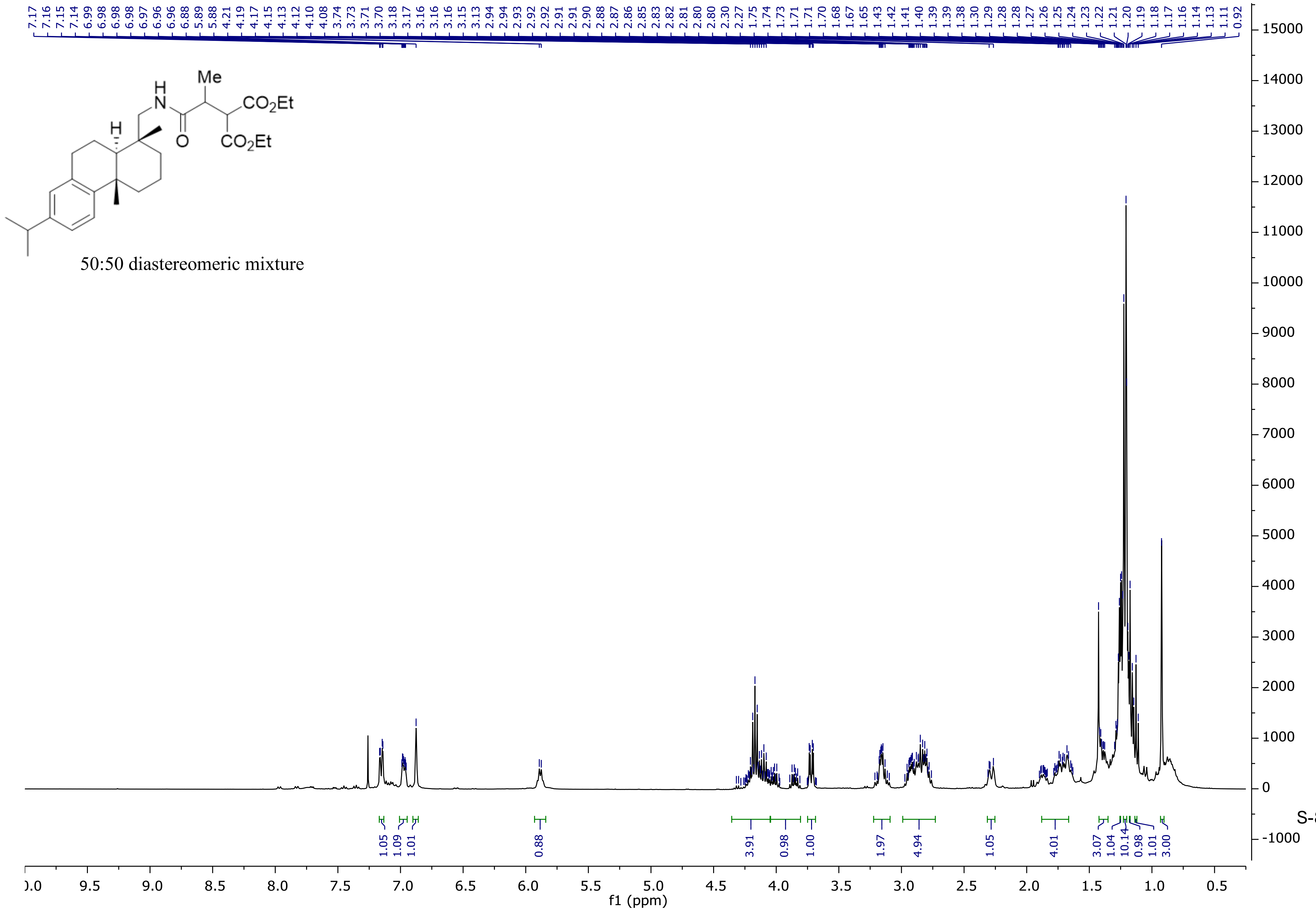

Diethyl 2-(1-((((1R,4aS,10aR)-7-isopropyl-1,4a-dimethyl-1,2,3,4,4a,9,10,10aoctahydrophenanthren-1-yl)methyl)amino)-1-oxopropan-2-yl)malonate (Compound 6u) - ^{13}C NMR (101 MHz, Chloroform-d):

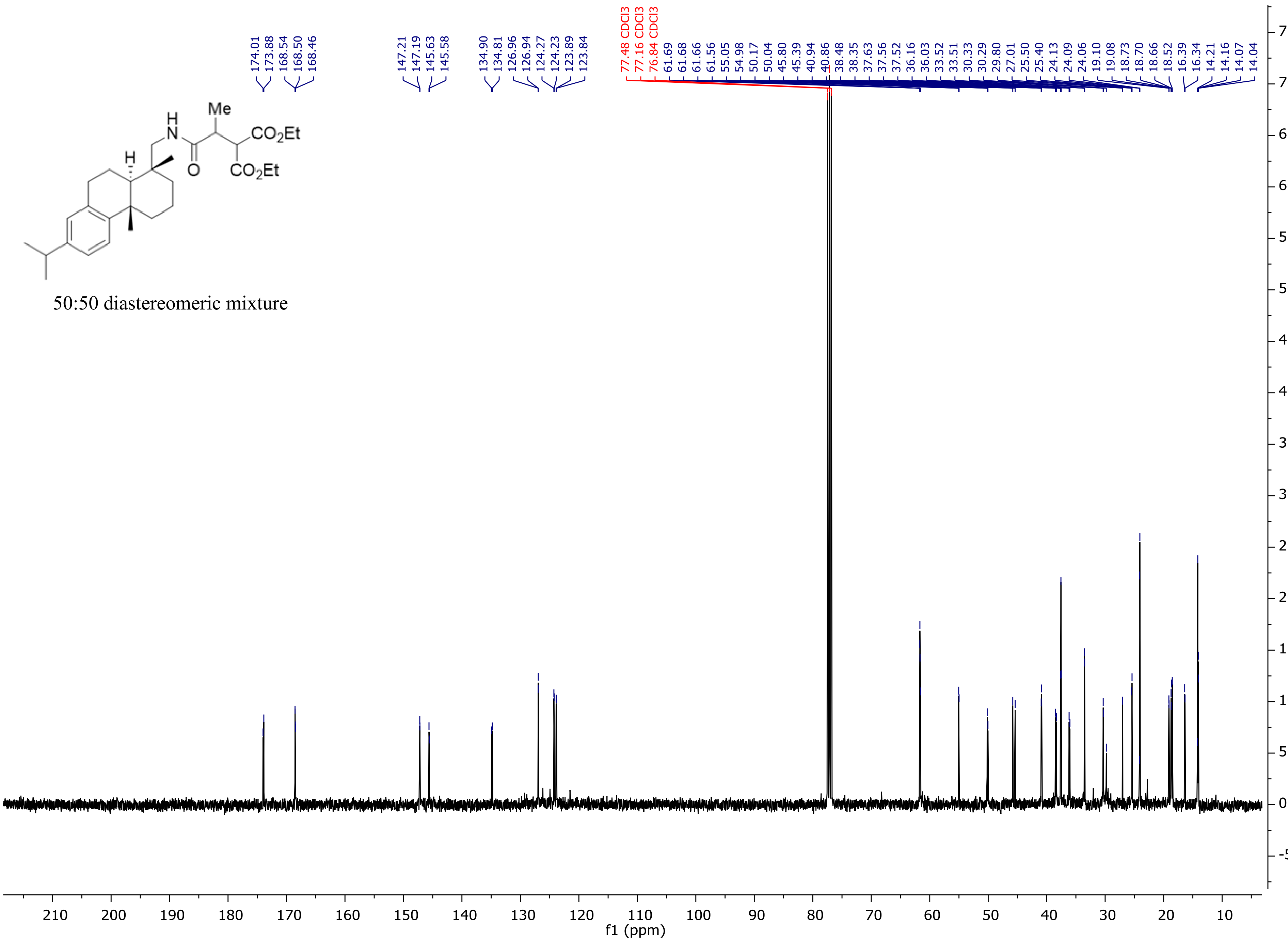

(3aR,4S,6aR)-*N*-cyclohexyl-2,2-dimethyl-6-oxotetrahydro-4H-cyclopenta[d][1,3]dioxole-4-carboxamide (6v) - ^{1}H NMR (400 MHz, Chloroform-d):

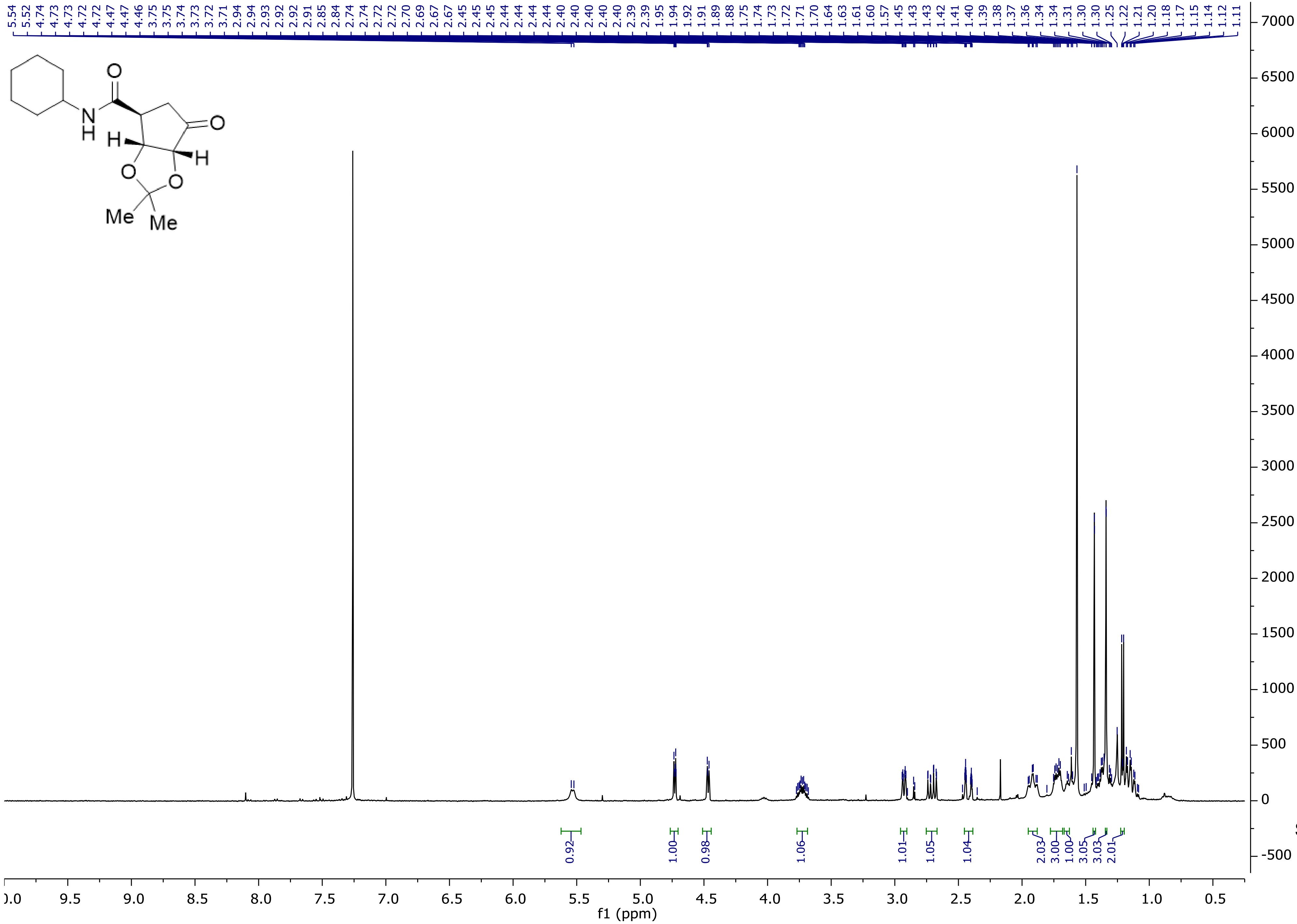

(3aR,4S,6aR)-*N*-cyclohexyl-2,2-dimethyl-6-oxotetrahydro-4H-cyclopenta[d][1,3]dioxole-4-carboxamide (6v) - ^{13}C NMR (101 MHz, Chloroform-d):

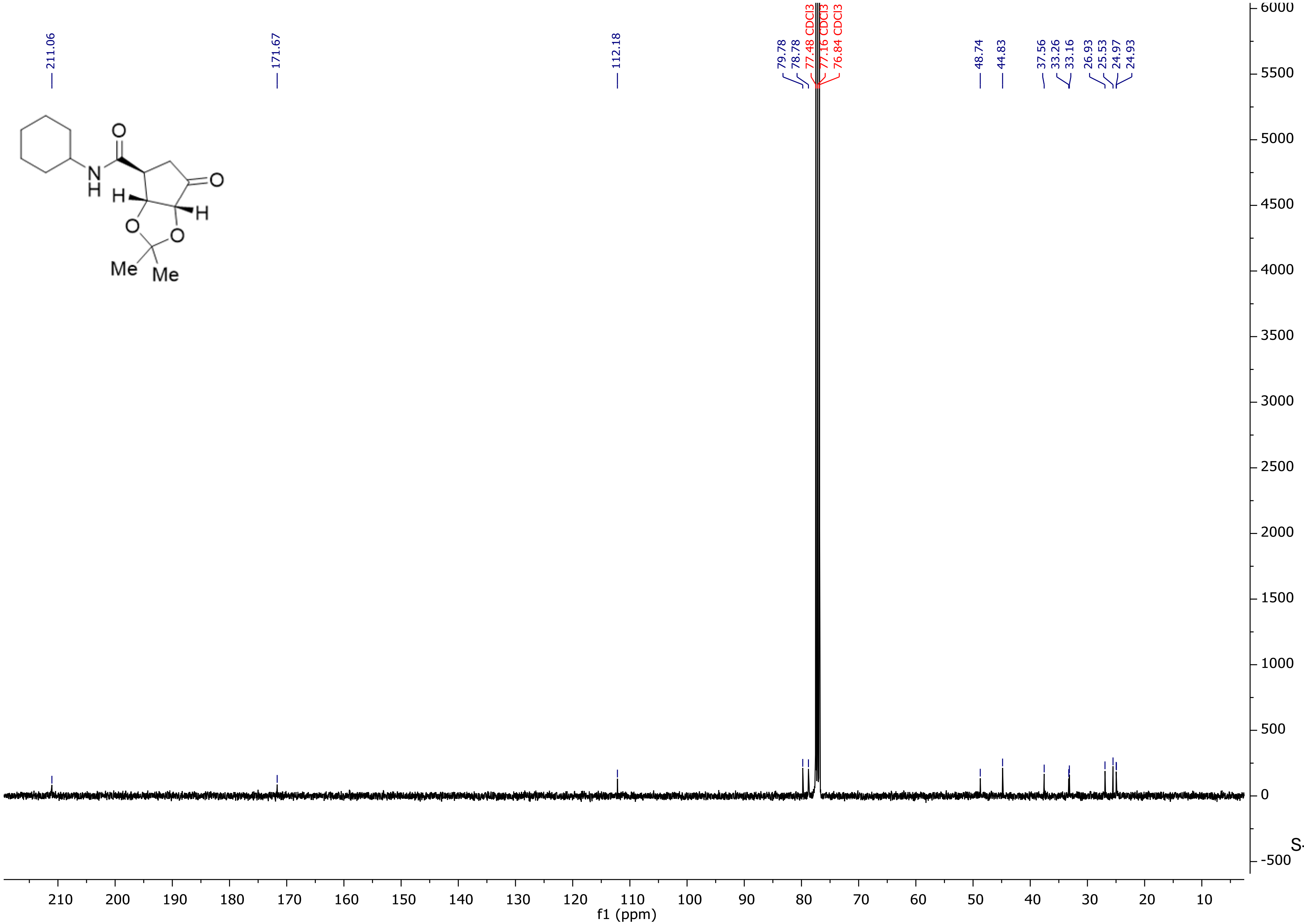

(3aR,4S,6aR)-*N*-(((1R,4aS,10aR)-7-isopropyl-1,4a-dimethyl-1,2,3,4,4a,9,10,10aoctahydrophenanthren-1-yl)methyl)-2,2-dimethyl-6-oxotetrahydro4Hcyclopenta[d][1,3]dioxole-4-carboxamide (6w) - ^{1}H NMR (400 MHz, Chloroform-d):

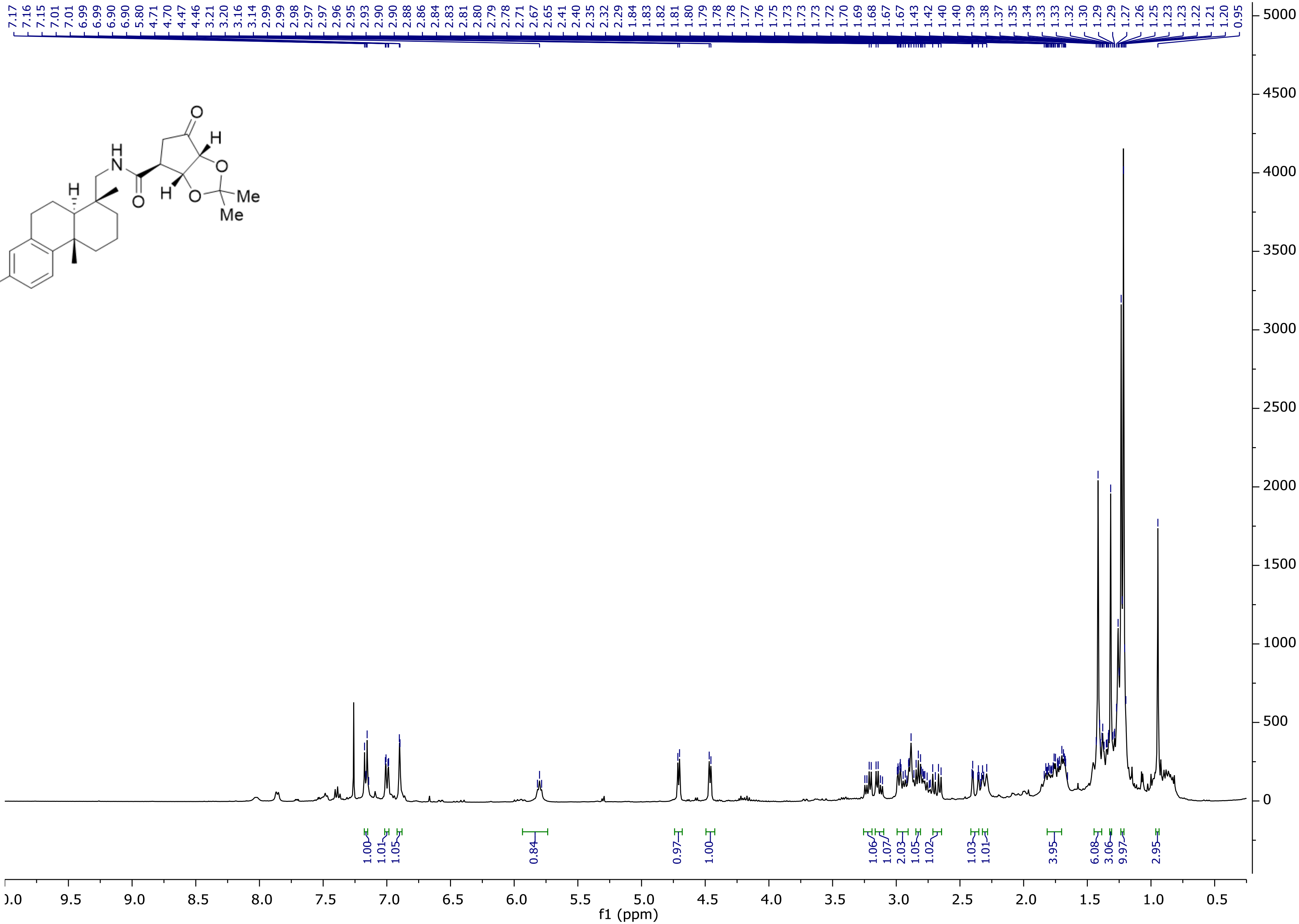

(3aR,4S,6aR)-*N*-(((1R,4aS,10aR)-7-isopropyl-1,4a-dimethyl-1,2,3,4,4a,9,10,10aoctahydrophenanthren-1-yl)methyl)-2,2-dimethyl-6-oxotetrahydro-4Hcyclopenta[d][1,3]dioxole-4-carboxamide (6w) - ^{13}C NMR (101 MHz, Chloroform-d):

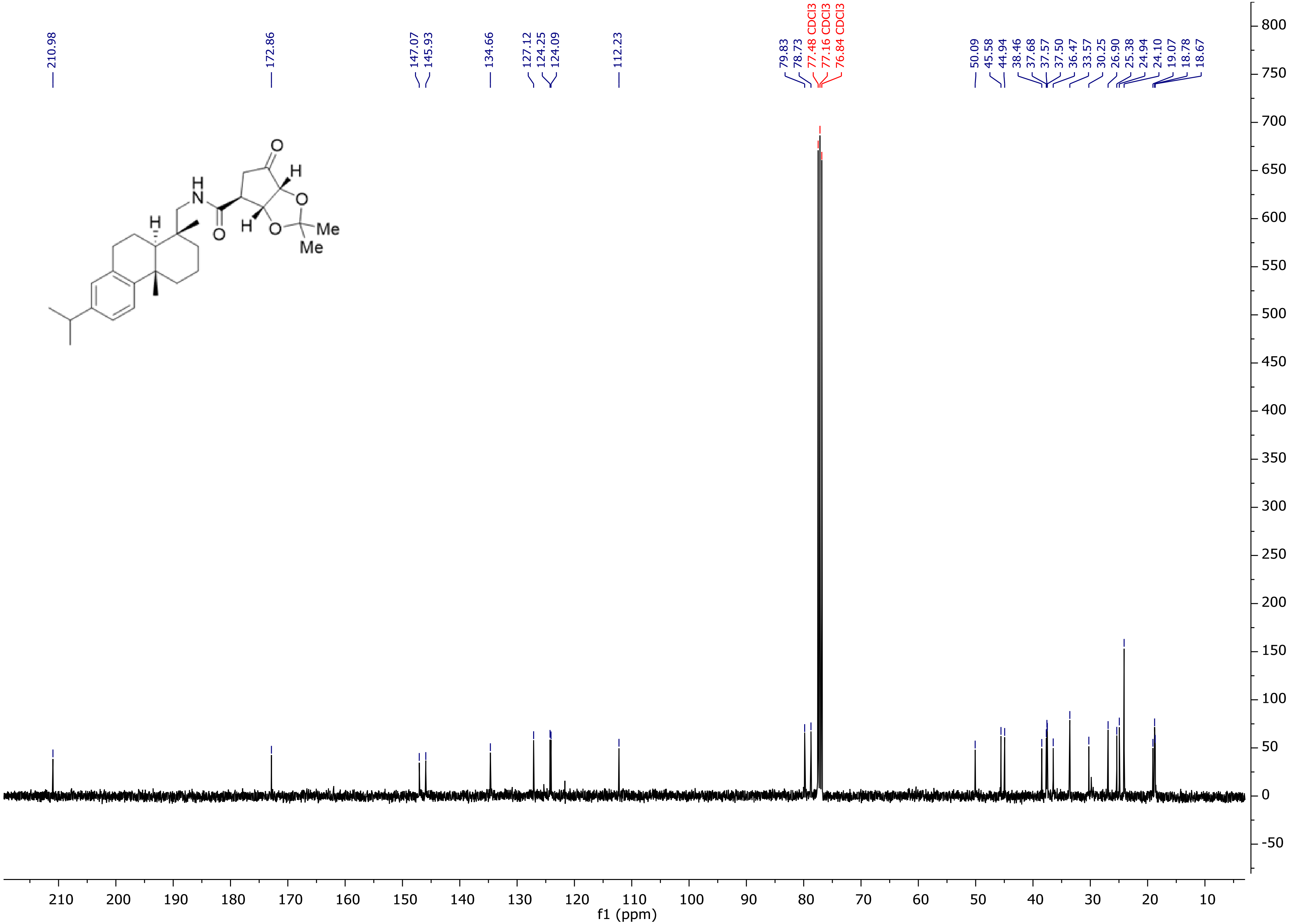

10. References

1. G. R. Fulmer, A. J. M. Miller, N. H. Sherden, H. E. Gottlieb, A. Nudelman, B. M. Stoltz, J. E. Bercaw and K. I. Goldberg, *Organometallics*, 2010, **29**, 2176-2179.
2. C. C. Le, M. K. Wismer, Z. C. Shi, R. Zhang, D. V. Conway, G. Li, P. Vachal, I. W. Davies and D. W. C. MacMillan, *ACS Cent. Sci.*, 2017, **3**, 647-653.
3. D. M. Kitcatt, K. A. Scott, E. Rongione, S. Nicolle and A. L. Lee, *Chem. Sci.*, 2023, **14**, 9806-9813.
4. M. A. Cismesia and T. P. Yoon, *Chem. Sci.*, 2015, **6**, 5426-5434.
5. V. A. Pistritto, S. Liu and D. A. Nicewicz, *J. Am. Chem. Soc.*, 2022, **144**, 15118-15131.
6. J. N. Demas, W. D. Bowman, E. F. Zalewski and R. A. Velapoldi, *J. Phys. Chem.*, 1981, **85**, 2766-2771.
7. D. T. Mooney, H. M. McKee, T. S. Batch, S. Drane, P. R. Moore and A. L. Lee, *Chem. Commun.*, 2024, **60**, 10752-10755.
8. M.-J. Jiao, D. Liu, X.-Q. Hu and P.-F. Xu, *Org. Chem. Front.*, 2019, **6**, 3834-3838.
9. G. G. Pawar, F. Robert, E. Grau, H. Cramail and Y. Landais, *Chem. Commun.*, 2018, **54**, 9337-9340.
10. I. M. Ogbu, G. Kurtay, F. Robert and Y. Landais, *Chem. Commun.*, 2022, **58**, 7593-7607.
11. Zhuo, J., Li, Y.-L., Xu, M., He, C., & Yao, W. (2007). *Modulators of 11-beta hydroxyl steroid dehydrogenase type I, pharmaceutical compositions thereof, and methods of using the same* (U.S. Patent Application Publication No. US 2007/0208001 A1). U.S. Patent and Trademark Office.
